# Mortality in Persons With Autism Spectrum Disorder or Attention-Deficit/Hyperactivity Disorder: A Systematic Review and Meta-analysis

**DOI:** 10.1001/jamapediatrics.2021.6401

**Published:** 2022-02-14

**Authors:** Ferrán Catalá-López, Brian Hutton, Matthew J. Page, Jane A. Driver, Manuel Ridao, Adolfo Alonso-Arroyo, Alfonso Valencia, Diego Macías Saint-Gerons, Rafael Tabarés-Seisdedos

**Affiliations:** 1 Knowledge Synthesis Group, Clinical Epidemiology Program, Ottawa Hospital Research Institute, Ottawa, Ontario, Canada; 2 Department of Medicine, University of Valencia/INCLIVA Health Research Institute and Centro de Investigación en Red de Salud Mental (CIBERSAM), Valencia, Spain; 3 Department of Health Planning and Economics, National School of Public Health, Institute of Health Carlos III, Madrid, Spain; 4 School of Epidemiology and Public Health, University of Ottawa, Ottawa, Ontario, Canada; 5 School of Public Health and Preventive Medicine, Monash University, Melbourne, Australia; 6 Geriatric Research Education and Clinical Center, Veterans Affairs Boston Healthcare System, Boston, Massachusetts; 7 Division of Aging, Department of Medicine, Brigham and Women’s Hospital, Harvard Medical School, Boston, Massachusetts; 8 Department of Medical Oncology, Dana-Farber Cancer Institute, Boston, Massachusetts; 9 Instituto Aragonés de Ciencias de la Salud, Red de Investigación en Servicios de Salud en Enfermedades Crónicas (REDISSEC), Zaragoza, Spain; 10 Department of History of Science and Documentation, University of Valencia, Valencia, Spain; 11 Unidad de Información e Investigación Social y Sanitaria, University of Valencia, Spanish National Research Council, Valencia, Spain; 12 Life Sciences Department, Barcelona Supercomputing Center, Barcelona, Spain

## Abstract

**Importance:**

Autism spectrum disorder (ASD) and attention-deficit/hyperactivity disorder (ADHD) are childhood-onset disorders that may persist into adulthood. Several studies have suggested that they may be associated with an increased risk of mortality; however, the results are inconsistent.

**Objective:**

To assess the risk of mortality among persons with ASD or ADHD and their first-degree relatives.

**Data Sources:**

A search of MEDLINE, Embase, Scopus, Web of Science, and PsycINFO (published from inception to April 1, 2021) was supplemented by searching reference lists of the retrieved articles.

**Study Selection:**

Cohort and case-control studies that reported mortality rate ratios (RRs) in persons with ASD or ADHD and/or their first-degree relatives compared with the general population or those without ASD/ADHD were included.

**Data Extraction and Synthesis:**

Screening, data extraction, and quality assessment were performed by at least 2 researchers independently. A random-effects model was used to meta-analyze individual studies and assessed heterogeneity (*I^2^*).

**Main Outcomes and Measures:**

All-cause mortality in association with ASD or ADHD. Secondary outcome was cause-specific mortality.

**Results:**

Twenty-seven studies were included, with a total of 642 260 individuals. All-cause mortality was found to be higher for persons with ASD (154 238 participants; 12 studies; RR, 2.37; 95% CI, 1.97-2.85; *I^2^*, 89%; moderate confidence) and persons with ADHD (396 488 participants; 8 studies; RR, 2.13; 95% CI, 1.13-4.02; *I^2^*, 98%; low confidence) than for the general population. Among persons with ASD, deaths from natural causes (4 studies; RR, 3.80; 95% CI, 2.06-7.01; *I^2^*, 96%; low confidence) and deaths from unnatural causes were increased (6 studies; RR, 2.50; 95% CI, 1.49-4.18; *I^2^*, 95%; low confidence). Among persons with ADHD, deaths from natural causes were not significantly increased (4 studies; RR, 1.62; 95% CI, 0.89-2.96; *I^2^*, 88%; low confidence), but deaths from unnatural causes were higher than expected (10 studies; RR, 2.81; 95% CI, 1.73-4.55; *I^2^*, 92%; low confidence).

**Conclusions and Relevance:**

This systematic review and meta-analysis found that ASD and ADHD are associated with a significantly increased risk of mortality. Understanding the mechanisms of these associations may lead to targeted strategies to prevent avoidable deaths in high-risk groups. The substantial heterogeneity between studies should be explored further.

## Key Points

**Question:**

Are persons with autism spectrum disorder (ASD) or attention-deficit/hyperactivity disorder (ADHD) at a higher risk of dying compared with the general population?

**Findings:**

In this systematic review and meta-analysis of 27 studies, persons with ASD or ADHD had higher mortality rates than the general population. When causes of death were examined, ASD and ADHD were associated with higher mortality due to unnatural causes (eg, injury, poisoning, and other), and only persons with ASD had an increased risk of mortality from natural causes of death (eg, neurologic, respiratory system, and cancer).

**Meaning:**

Having ASD or ADHD may be associated with higher mortality risks.

## Teaser

This systematic review and meta-analysis evaluates the risk of both all-cause and cause-specific mortality among persons with autism spectrum disorder or attention-deficit/hyperactivity disorder and their first-degree relatives.

## Introduction

Autism spectrum disorder (ASD) and attention-deficit/hyperactivity disorder (ADHD) are common childhood onset neurodevelopmental disorders,^1,2^ with estimated worldwide prevalence of 58.6 million cases among children and young people 20 years and younger.^2^ Although the cause of ASD and ADHD remains largely unknown, a complex interaction of multiple factors is thought to contribute to the development of both conditions, generally persisting into adulthood.^3,4,5^ Both disorders have been found to be associated with psychosocial functional impairments and a range of adverse outcomes in patients and their families.^4,5,6,7,8,9^

Deaths in people with mental disorders have been examined in multiple epidemiologic studies^10,11,12^ suggesting that people with these disorders may experience a significant reduction in life expectancy, with increased mortality rates over the general population. Similarly, several studies^13,14,15,16,17,18,19^ have suggested that persons with ASD or ADHD may be associated with an increased risk of mortality; however, the results are inconsistent. For example, Pickett et al^13^ reported a 2-fold higher mortality rate ratio (RR) of 2.4 (95%, 2.2-2.8) in persons with ASD compared with those without it in the California Development Disability System. Bilder et al^14^ showed a mortality RR of 11.59 (95% CI, 6.24-21.53) in the Utah/University of California, Los Angeles Autism Epidemiologic Study. However, Smith et al^15^ suggested a nonstatistically significant increase in mortality (RR, 1.10; 95% CI, 0.50-2.50) in a large cohort study of school pupils with and without ASD. Conversely, Dalsgaard et al^16^ and Sun et al^17^ suggested increased mortality risk in persons with ADHD in large population-based cohorts from Denmark (fully adjusted RR, 2.07; 95% CI, 1.70-2.50) and Sweden (RR, 3.94; 95% CI, 3.51-4.43), respectively. London and Landes^18^ suggested similar results, with an adjusted RR of 1.78 (95% CI, 1.01-3.12). More recently, a nationwide population-based cohort study in Taiwan^19^ showed that having ADHD was associated with having a marginally increased risk of mortality after full adjustment (fully adjusted hazard ratio, 1.07; 95% CI, 1.00-1.17).

To our knowledge, no systematic review and meta-analysis of epidemiologic studies has specifically evaluated the mortality risk and main causes of death in persons with ASD or ADHD. A systematic review^20^ of studies published until 2010 examined ASD and 2 outcomes (epilepsy and mortality). Only 5 studies investigating mortality associations were located,^20^ so lack of data precluded exploring potential variations in all-cause mortality and quantifying cause-specific mortality risks. Therefore, in our study, we conducted a comprehensive knowledge synthesis to identify and evaluate all available observational studies of mortality associations in people with ASD or ADHD, which provide more complete information, and the most updated epidemiologic evidence than previous studies. Our systematic review and meta-analysis of epidemiologic studies for ASD and ADHD has only become possible in the past few years because several large population-based observational studies^16,17,19,21^ have been published that have reported similar mortality-related outcome measures. In this study, we aimed to evaluate the risk of both all-cause and cause-specific mortality among persons with ASD or ADHD and their first-degree relatives.

## Methods

Our systematic review was reported in accordance with the 2020 Preferred Reporting Items for Systematic Reviews and Meta-analyses (PRISMA) reporting guideline.^22^ We also reported this study in accordance with the guidance provided in the Meta-analysis of Observational Studies in Epidemiology (MOOSE) reporting guideline^23^ (eTables 1 and 2 in the Supplement). We developed and registered a review protocol (PROSPERO registration number: CRD42017059955). Our methods are briefly described here (and explained in more detail in the protocol^24^ and in eTables 1-12 in the Supplement).

### Information Sources and Search Strategy

An information specialist (A.A.-A.) searched MEDLINE (via PubMed), Scopus, Embase, Web of Science Core Collection, and PsycINFO (via ProQuest) to identify all relevant observational studies in humans that examined the risk of mortality among persons with ASD or ADHD, published from inception to April 1, 2021, without language restrictions. The search strategies for all electronic databases are presented in the eTable 5 in the Supplement. References of all relevant primary studies and review articles were also screened to identify additional data sources.

### Eligibility Criteria

To be included, primary studies had to be observational (cohort, case-control) studies of persons with ASD or ADHD according to standard operationalized diagnostic criteria (eg, *International Classification of Diseases, Ninth Revision [ICD-9] *or *Tenth revision [ICD-10]* or third, fourth, or fifth editions of the *Diagnostic and Statistical Manual of Mental Disorders* criteria; ASD, *ICD-9* codes 299.0 and 299.8 and *ICD-10* code F84; ADHD, *ICD-9* codes 314.00 and 314.01 and *ICD-10* code F90) and report the mortality RR for the risk of mortality in people with ASD/ADHD compared with the general population or those without ASD/ADHD, or have enough data (eg, number of cases and sample size; observed and expected cases) to compute these estimates. We excluded studies in which ASD or ADHD were not the exposure of interest and mortality was not reported as the outcome. Studies not presenting study-specific data or sufficient data for an outcome measure to be calculated were also excluded. We excluded case reports, case series, in vitro studies, and animal studies.

### Outcomes

The primary outcome was all-cause mortality (*ICD-10* codes A00-Y98 and *ICD-9* codes 001-E999). Cause-specific mortality rates were evaluated as a secondary outcome of interest (eTable 3 in the Supplement).

### Screening and Selection Process

Two investigators (F.C.-L., D.M.S.-G., and/or M.R.) independently screened all titles and abstracts and retrieved the full text of any article considered definitely or possibly eligible. The same investigators reviewed the full-text articles against the eligibility criteria. Any disagreement between these investigators was resolved by discussion.

### Data Extraction and Quality Assessment

Two investigators (F.C.-L., D.M.S.-G., and/or M.R.) independently extracted relevant information from the included studies: first author, publication year and period of recruitment, country, study design, setting, coverage, mean or median age (or age range), proportion of women participants, ethnicity, comorbidities, social status, diagnostic criteria and ascertainment of outcome, consideration of confounding factors, the number of participants with fatal outcome, and maximally adjusted RR estimates with 95% CIs. When relevant fatal outcome data were not available, we directly contacted the corresponding author of the study to request the information (eTable 7 in the Supplement). Two pairs of investigators (F.C.-L., M.J.P., D.M.S.-G., and M.R.) independently undertook methodologic quality assessment of included studies using the Newcastle-Ottawa scale^25^ and allocated stars for adherence to the prespecified criteria. This scale ranges from 0 (lowest quality) to 9 (highest quality) stars and judges each study regarding selection of study groups, comparability, and ascertainment of the outcome. We considered studies with 0 to 3, 4 to 6, and 7 to 9 stars to represent high, moderate, and low risk of bias, respectively. Discrepant scores were resolved by discussion among investigators.

### Data Analysis

To measure the association between the mortality in people with ADHD or ASD and the mortality in the reference control group, we did meta-analysis using the inverse variance random effects model^26^ (with the DerSimonian-Laird between-study variance estimator) to pool weighted all-cause and specific-cause mortality RR estimates. We explored the contributions from natural and unnatural causes of death. Unnatural deaths were defined based on *ICD* codes (*ICD-10* codes V01-Y98 or *ICD-9* codes E800-999); the remaining deaths were classified as natural. All meta-analyses were conducted for ADHD and ASD separately. We assessed heterogeneity between studies using the *P* value of Cochran Q test^27^ and the *I^2^* statistic (with 95% CIs),^28^ which could reflect either genuine heterogeneity or bias. The *I^2^* statistic ranges between 0% and 100% (with values of 0%-25% and 75%-100% taken to indicate low and considerable heterogeneity, respectively). We also calculated 95% prediction intervals, which further account for heterogeneity between studies and indicate the uncertainty for the effect that would be expected in a new study examining that same association.^29^ To assess the robustness of pooled results and explore possible reasons for heterogeneity, prespecified subgroup analyses^24^ were performed according to sex (male or female), number of comorbidities (including 0, 1, 2, or any), age group at first diagnosis of ASD or ADHD (prior to age 18 years or at 18 years or older), number of participants with ASD or ADHD (<500 vs 500-1000 vs >1000), setting (community, inpatient care, or outpatient care), follow-up (less than 1 year, 1-5 years, >5 years), study quality (low vs moderate vs high risk of bias), and statistical adjustment for potential confounders (yes vs no). Small study effects (the tendency for exposure effects estimated in smaller studies to differ from those estimated in larger studies, which can result from reporting biases, methodologic heterogeneity, epidemiologic heterogeneity, or other factors) was estimated visually by funnel plots or by Begg test and the weighted regression test of Egger. *P* values were determined using the Woolf method. *P* values were 2-tailed, and statistical significance was set at less than .05. We performed all data analyses using Stata version 17 (StataCorp).

### Credibility Assessment

We applied a set of criteria to classify the credibility (or certainty) of the evidence from meta-analysis based on the Global Burden of Disease^30^ and GRADE system^31^ (eTable 10 in the Supplement).

With the publication of this article, the full data set will be freely available online in the Open Science Framework,^32^ a secure online repository for research data. Amendments to the original protocol are listed in the eTable 4 in the Supplement.

## Results

### Extent of Relevant Literature Identified

**Figure 1.**
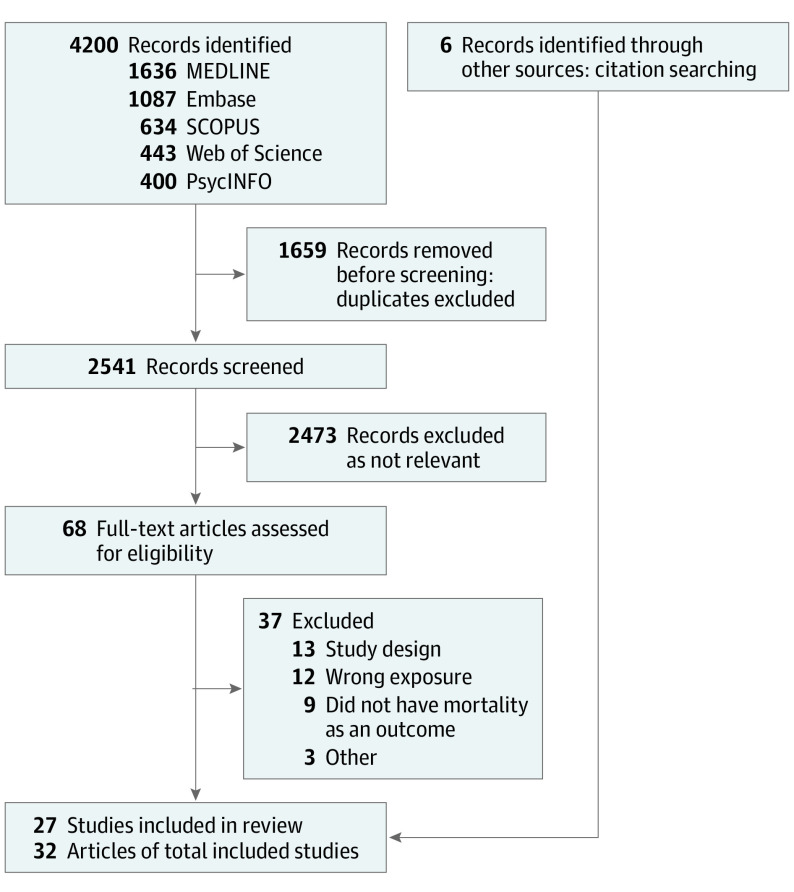
Flow Diagram of Study Selection

We screened 2541 titles and abstracts, followed by 68 full-text articles (Figure 1). Twenty-seven epidemiologic studies published in 32 articles^13,14,15,16,17,18,19,21,33,34,35,36,37,38,39,40,41,42,43,44,45,46,47,48,49,50,51,52,53,54,55,56^ met our inclusion criteria.

### Description of Studies and Participants

Table 1 and eTable 8 in the Supplement summarize the characteristics of the included studies. All studies were published in English from 1988 through 2021. Twenty-three studies^13,14,15,16,17,18,33,34,35,36,37,38,39,40,41,42,44,45,46,47,48,49,50,51,52,53,54,56^ were from North America and Europe (11 in the US,^13,14,18,33,34,35,36,37,41,42,46,50,51,53^ 5 in Denmark,^16,38,39,44,49,52,56^ 3 in Sweden,^17,40,47^ 2 in Finland,^45,54^ and 2 in the UK^15,48^). Seventeen studies were retrospective cohorts,^13,14,15,16,18,21,33,34,35,36,37,38,39,43,44,48,50,52,53,54,55,56^ 8 were prospective cohorts,^17,19,40,41,42,45,46,49^ and 2 were retrospective case-control studies.^47,51^ Fourteen studies^13,14,15,21,34,35,38,39,40,47,48,49,50,53,54,55,56^ included 206 162 participants with ASD, and 12 studies^16,17,18,19,33,36,37,41,42,44,45,46,51,52^ included 433 761 participants with ADHD. The number of participants with ASD ranged from 120 to 35 929, and the number of participants with ADHD ranged from 110 to 275 980. The proportion of female participants ranged from 14% to 100%. Median (range) follow-up was 16 (3-33) years. Twelve studies^15,21,33,36,37,40,42,46,48,50,52,55^ followed up participants to young adult age (mean age, <40 years). Only 3 studies^14,36,37,43^ included first-degree relatives of people with ADHD or ASD diagnosis.

**Table 1.** Summary Characteristics of Included Studies

Source	Study design (country)	Setting; coverage	Study years (follow-up, y)	No. of participants	Sex and age	No. of fatal cases	Main outcome(s)	Causes of death	Comparator
Kuperman et al,^33^ 1988	Retrospective cohort (US)	Inpatient; population-based	1970-1985 (9.1)	110 People with ADHD	20.0% Female; mean age, 12.3 y at admission and 22.1 y at follow-up	1	Specific cause of death	Yes	General population
Pickett et al,^13^ 2006; Shavelle et al,^34^ 2001; Shavelle et al,^35^ 1998	Retrospective cohort (US)	Community; population-based	1983-1997 and 1998-2002 (9.5)	13 111 People with ASD	20.6% Female; most aged ≥4 y at first evaluation	280	All-cause and specific cause of death	Yes	General population
Barkley et al,^36^ 2008; Barkley et al,^37^ 1990	Retrospective cohort (US)	Outpatient; health services	1979-1996 (17)	158 People with ADHD and their parents	9% Female; aged ≥4 y at first evaluation and 21 y at follow-up	3	All-cause and specific cause of death	Yes	Participants without hyperactivity
Mouridsen et al,^38^ 2008; Isager et al,^39^ 1999	Retrospective cohort (Denmark)	Inpatient; population-based	1960-2006 (35.5)	341 People with ASD	24.9% Female; mean age, 7.5 y at admission and 43.4 y at follow-up	26	All-cause and specific cause of death	Yes	General population
Gillberg et al,^40^ 2010	Prospective cohort (Sweden)	Community; population-based	1962-2008 (33.2)	120 People with ASD	29.2% Female; early childhood at admission and mean age, 33.2 y at follow-up	9	All-cause and specific cause of death	Yes	General population
Klein et al,^41^ 2012	Prospective cohort (US)	Community; school-based	1970-NA (33)	207 People with ADHD	All male; mean age, 8.3 y at first evaluation and 41 y at follow-up	15	All-cause of death	No	Participants without ADHD
Barbaresi et al,^42^ 2013	Prospective cohort (US)	Community; population-based	1976-2009 (NA)	367 People with ADHD	28.0% Female; mean age, 10.4 y at diagnosis and 27.0 y at follow-up	7	All-cause and specific cause of death	Yes	Participants without ADHD
Bilder et al,^14^ 2013	Retrospective cohort (US)	Community; population-based	1982-2011 (25)	305 People with ASD and their siblings	25.2% Female; mean age, 10.8 y at diagnosis and 35.8 y at follow-up	29	All-cause and specific cause of death	Yes	General population; siblings
Fairthorne et al,^43^ 2014	Retrospective cohort (Australia)	Community; population-based	1983-2010 (NA)	2041 Mothers of children with ASD	All women; 77% aged 20-34 y with mean age at death of 42 y	24	All-cause and specific cause of death	Yes	Participants without ASD or intellectual disability
Dalsgaard et al,^16^ 2015; Scott et al,^44^ 2017	Retrospective cohort (Denmark)	Community; population-based	1981-2013 (32)	32 061 People with ADHD	26.4% Female; mean age, 12.3 y at diagnosis	107	All-cause and specific cause of death	Yes	General population
Koisaari et al,^45^ 2015	Prospective cohort (Finland)	Community; health services (maternity hospital)	1971-2004 (40)	122 People with ADHD	29.5% Female; aged ≥5 y at first evaluation and mean age of 40 y at follow-up	11	All-cause and specific cause of death	Yes	Participants without ADHD
Hetchtman et al,^46^ 2016	Prospective cohort (US)	Community; school-based	1992-2008 (16)	476 People with ADHD	19.7% Female; mean age, 8.5 y at diagnosis and 24.7 y at follow-up	11	All-cause and specific cause of death	Yes	Participants without ADHD
Hirvikoski et al,^47^ 2016	Retrospective case-cohort/case-control (Sweden)	Mixed; population-based	1987-2009 (NA)	27 122 People with ASD	31.1% Female; mean age, 19.8 y at diagnosis	706	All-cause and specific cause of death	Yes	General population
Hosking et al,^48^ 2016	Retrospective cohort (UK)	Outpatient; population-based	2009-2013 (3)	1532 People with ASD	58.1% Female; mean age, 39.9 y at follow-up	15	All-cause of death	No	General population
London and Landes,^18^ 2016	Retrospective cohort (US)	Community; population-based	2007-2011 (4)	654 People with ADHD	54.4% Female; mean age, 47.6 y at follow-up	13	All-cause and specific cause of death	Yes	General population
Schendel et al,^49^ 2016	Prospective cohort (Denmark)	Community; population-based	1980-2013 (NA)	20 492 People with ASD	22.4% Female; most aged >9 y at diagnosis with median age of 19.0 y at death	68	All-cause and specific cause of death	Yes	General population
Chen et al,^19^ 2019	Prospective cohort (Taiwan)	Mixed; population-based	2000-2013 (14)	275 980 People with ADHD	24.1% Female; mean age, 9.6 y at evaluation	727	All-cause and specific cause of death	Yes	General population
Hwang et al,^21^ 2019	Retrospective cohort (Australia)	Community; population-based	2001-2015 (NA)	35 929 People with ASD	20.5% Female; age ranged, 5-64 y and mean age, 35.0 y at death	244	All-cause and specific cause of death	Yes	General population
Kirby et al,^50^ 2019	Retrospective cohort (US)	Community; population-based	1998-2017 (20)	16 904 People with ASD	24.0% Female; mean age, 18.4 y at follow-up	49	Specific cause of death	Yes	General population
Sun et al,^17^ 2019	Prospective cohort (Sweden)	Community; population-based	1983-2013 (11.1)	86 670 People with ADHD	33.2% Female; mean age, 14.3 y at diagnosis	424	All-cause and specific cause of death	Yes	General population
Yeh et al,^51^ 2019	Retrospective case-control (US)	Mixed; population-based	2000-2013 (NA)	4416 People with ADHD	22.5% Female; most aged 40-64 y at evaluation	67	Specific cause of death	Yes	General population
Fitzgerald et al,^52^ 2019	Retrospective cohort (Denmark)	Community; population-based	1995-2014 (21.5)	32 540 People with ADHD	30.8% Female; mean age, 21.5 at follow-up	35	Specific cause of death	Yes	General population
Akobirshoev et al,^53^ 2020	Retrospective cohort (US)	Inpatient; population-based	2004-2014 (NA)	34 237 People with ASD	24.7% Female; mean age, 33.1 y at evaluation	462	All-cause of death	No	General population
Jokiranta-Olkoniemi et al,^54^ 2021	Retrospective cohort (Finland)	Mixed; population-based	1987-2015 (NA)	4695 People with ASD	20.4% Female; mean age, 8.0 y at diagnosis	53	All-cause and specific cause of death	Yes	Participants without ASD
Huang et al,^55^ 2021	Retrospective cohort (Taiwan)	Mixed; population-based	2000-2015 (8.1)	6599 People with ASD	22.8% Female; mean age, 11.9 y at evaluation	119	All-cause of death	No	General population
Kõlves et al,^56^ 2021	Retrospective cohort (Denmark)	Community; population-based	1995-2016 (NA)	35 020 People with ASD	26.6% Female; most aged 10-29 y	53	Specific cause of death	Yes	General population
Smith et al,^15^ 2021	Retrospective cohort (Scotland, UK)	Community; school-based	2008-2015 (3.9)	9754 People with ASD	14.0% Female; most aged 10-19 y	6	All-cause of death	No	Participants without ASD

Abbreviations: ADHD, attention-deficit/hyperactivity disorder; ASD, autism spectrum disorder; NA, not available.

There were 49 individual study estimates for all-cause mortality in persons with ASD or ADHD and/or their first-degree relatives (eTable 9 in the Supplement). Seventeen studies^14,16,17,18,19,33,38,39,40,47,48,49,51,56^ provided adjusted RR estimates to control for several covariates (eg, age, sex, calendar year, or other). Twenty-one studies^13,14,16,17,18,19,21,33,34,35,36,37,38,39,40,42,43,44,45,46,47,49,50,51,52,54,56^ reported information on 12 cause-specific mortality outcomes. The most studied *ICD-10* categories were external causes of mortality (such as injuries, unintentional incidents, suicides, and poisoning), accounting for 1215 cases (61.2%) in 21 studies^13,14,16,17,19,21,33,34,35,36,37,38,39,40,42,43,44,45,46,47,49,50,51,52,54,56^; followed by diseases of the circulatory system, accounting for 190 cases (9.6%) in 8 studies^14,17,21,38,39,40,43,45,47^ and neoplasms, accounting for 168 cases (8.5%) in 7 studies^13,14,17,21,34,35,40,43,47^ (eFigure 23 in the Supplement). Infectious diseases accounted 8 cases (0.4%) in 4 studies^14,38,39,40,47^ and complications of pregnancy, childbirth, and puerperium only accounted 1 case (0.1%) in 1 study.^43^ Regarding the methodologic quality (eTable 10 in the Supplement), 16 studies^13,14,16,17,19,21,33,34,35,38,39,40,42,44,47,48,49,52,55,56^ (59%) had low risk of bias and 11 studies^15,18,36,37,41,43,45,46,49,51,53,54^ had moderate risk of bias (Newcastle-Ottawa Scale values, 4-9).

### Main Findings From Meta-analyses

**Figure 2.**
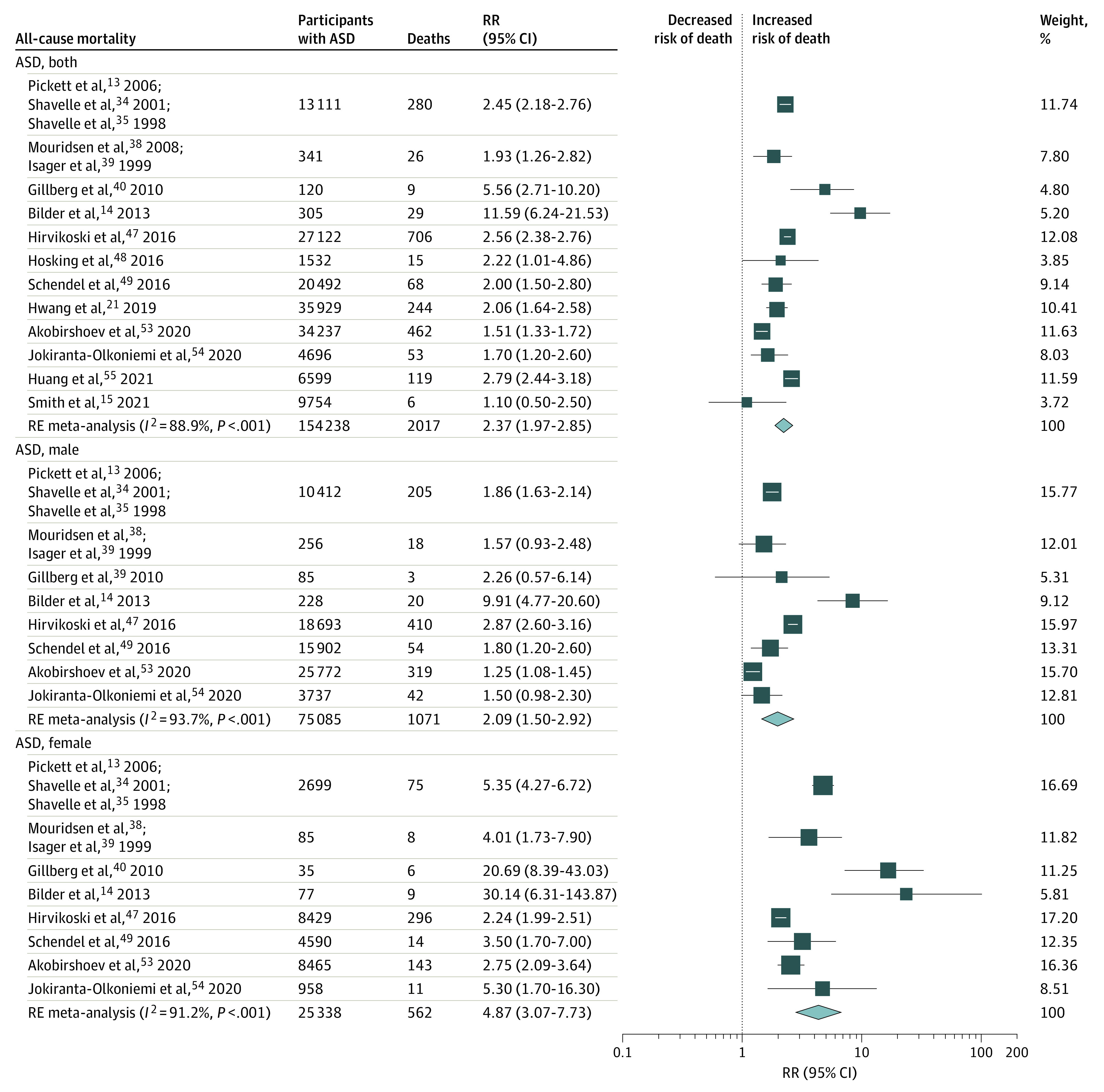
Risk of All-Cause Mortality in People With Autism Spectrum Disorder (ASD) The size of each box indicates the effect of each study by weight assigned using the random-effects (RE) model; diamond, estimated effect size; and width of diamond, the precision of the estimate (95% CI). RR indicates risk ratio.

**Figure 3.**
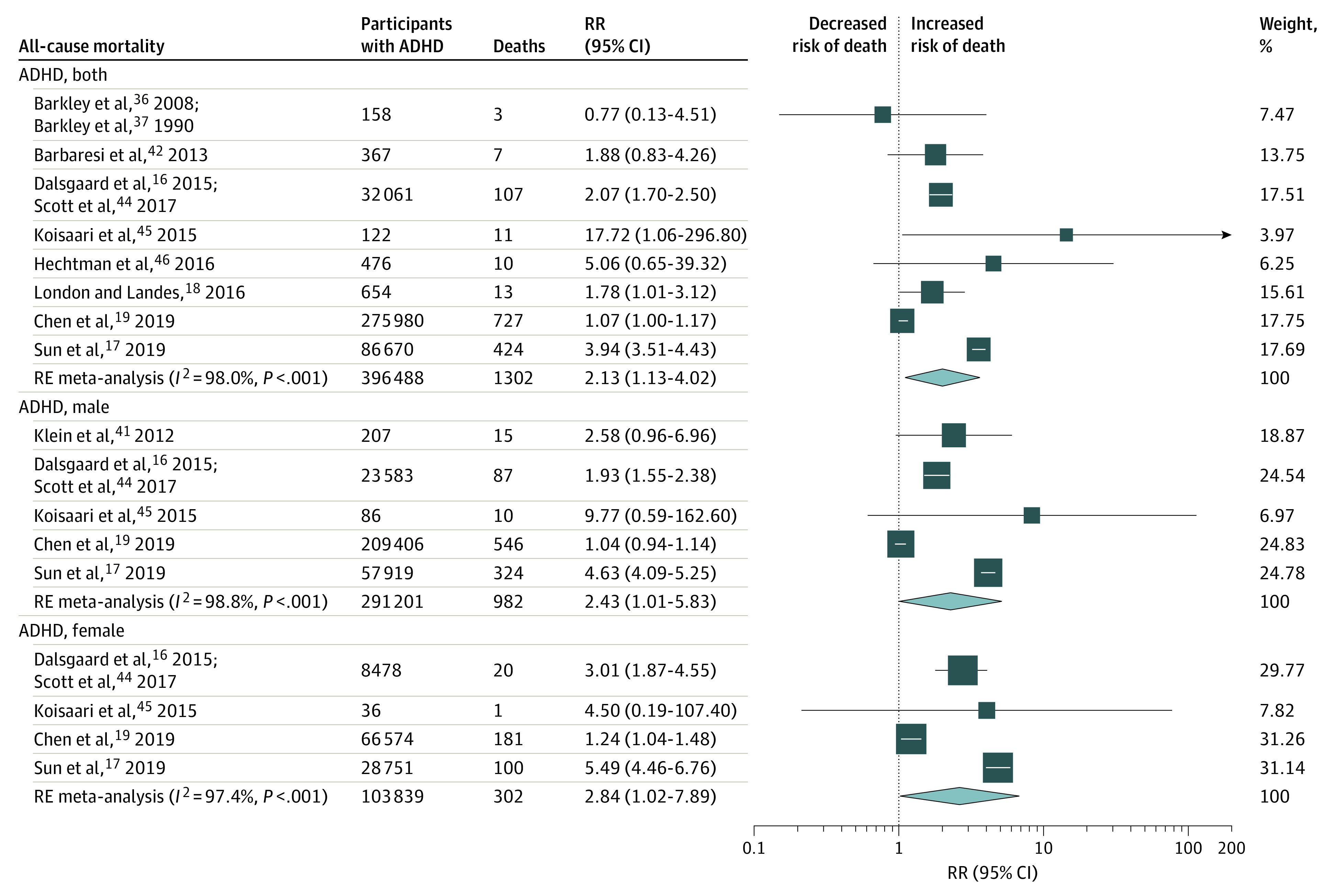
Risk of All-Cause Mortality in People With Attention-Deficit/Hyperactivity Disorder (ADHD) The size of each box indicates the effect of each study by weight assigned using the random-effects (RE) model; diamond, estimated effect size; and width of diamond, the precision of the estimate (95% CI). RR indicates risk ratio.

Our all-cause meta-analyses focused the risk of dying in persons with ASD or ADHD compared with the general population and included 21 studies^13,14,15,16,17,18,19,21,34,35,36,37,38,39,40,41,42,44,45,46,47,48,49,53,54,55^ that contributed 46 data points (Table 2; eFigure 23 and eTable 9 in the Supplement). Thirty-four of 46 all-cause mortality RR estimates (74%) suggested an increased risk of mortality in individuals with ASD or ADHD. Among participants with ASD, all-cause mortality was increased in both sexes combined (12 studies^13,14,15,21,34,35,38,39,40,47,48,49,53,54,55^; RR, 2.37; 95% CI, 1.97-2.85; *I^2^*, 89%; moderate confidence) and separately for male individuals (8 studies^13,14,34,35,38,39,40,47,49,53,54^; RR, 2.09; 95% CI, 1.50-2.92; *I^2^*, 94%; low confidence) and female individuals (8 studies^13,14,34,35,38,39,40,47,49,53,54^; RR, 4.87; 95% CI, 3.07-7.73; *I^2^*, 91%; low confidence). Among persons with ADHD, all-cause mortality was also increased in both sexes combined (8 studies^16,17,18,19,36,37,42,44,45,46^; RR, 2.13; 95% CI, 1.13-4.02; *I^2^*, 98%; low confidence), and separately for male individuals (5 studies^16,17,19,41,44,45^; RR, 2.43; 95% CI, 1.01-5.83; *I^2^*, 99%; low confidence) and female individuals (4 studies^16,17,19,44,45^; RR, 2.84; 95% CI, 1.02-7.89; *I^2^*, 97%; low confidence) (Table 2 and Figure 2 and Figure 3). Meta-analysis for all-cause mortality among first-degree relatives was not possible, with only 3 studies reporting outcome data (1 study^14^ in siblings of children with ASD, 1 study^43^ in mothers of children with ASD, and 1 study^36,37^ in parents of children with ADHD).

**Table 2.** Description of Main Results of Meta-analysis for Association of ASD or ADHD With Risk of Mortality^a^

Outcomes of interest	No.			RR (95% CI)		*P* value for effect estimate	*I^2^* (95% CI)	95% Prediction interval	*P* value for heterogeneity	Confidence
	Studies	Participants with ASD or ADHD	Death cases	Pooled	Largest study					
**Primary outcome, all-cause mortality**										
All-cause mortality, ASD										
Both	12	154 238	2017	2.37 (1.97-2.85)	2.06 (1.64-2.58)	<.001	89 (82-93)	1.25-4.48	<.001	Moderate (probable)
Male	8	75 085	1071	2.09 (1.50-2.92)	1.25 (1.08-1.45)	<.001	94 (90-96)	0.68-6.40	<.001	Low (suggestive)
Female	8	25 338	562	4.87 (3.07-7.73)	2.75 (2.09-3.64)	<.001	91 (85-95)	1.09-21.78	<.001	Low (suggestive)
All-cause mortality, ADHD										
Both	8	396 488	1302	2.13 (1.13-4.02)	1.07 (1.00-1.17)	.02	98 (97-99)	0.28-16.42	<.001	Low (suggestive)
Male	5	291 201	982	2.43 (1.01-5.83)	1.04 (0.94-1.14)	.046	99 (98-99)	0.10-58.40	<.001	Low (suggestive)
Female	4	103 839	302	2.84 (1.02-7.89)	1.24 (1.04-1.48)	.045	97 (96-99)	0.03-277.30	<.001	Low (suggestive)
**Secondary outcomes, cause-specific mortality**										
Natural causes, ASD										
Both	4	65 421	613	3.80 (2.06-7.01)	2.59 (2.36-2.85)	<.001	96 (92-98)	0.22-66.96	<.001	Low (suggestive)
Male	2	22 430	256	2.95 (2.35-3.69)	2.85 (2.50-3.24)	<.001	10 (NA)	NA	.29	Low (suggestive)
Female	2	9387	220	6.57 (0.60-71.91)	2.44 (2.12-2.80)	.123	79 (NA)	NA	.03	Low (not conclusive)
Natural causes, ADHD										
Both	4	394 833	516	1.62 (0.89-2.96)	0.91 (0.80-1.15)	.1	88 (72-95)	0.11-23.10	<.001	Low (not conclusive)
Male	3	290 908	348	1.22 (0.74-2.01)	0.83 (0.72-1.15)	.43	80 (36-94)	0.00-451.06	.007	Low (not conclusive)
Female	3	103 803	138	2.02 (0.91-4.48)	1.03 (0.82-1.30)	.09	91 (77-97)	0.00-39.6x10^3^	<.001	Low (not conclusive)
Unnatural causes, ASD										
Both	6	117 345	318	2.50 (1.49-4.18)	3.75 (2.85-4.92)	.001	95 (92-97)	0.38-16.35	<.001	Low (suggestive)
Male	4	61 031	182	1.94 (0.83-4.49)	3.48 (2.57-4.74)	.12	96 (94-98)	0.03-112.04	<.001	Low (not conclusive)
Female	4	22 712	58	3.61 (1.71-7.60)	2.63 (1.46-4.76)	.001	79 (45-92)	0.15-88.22	.002	Low (suggestive)
Unnatural causes, ADHD										
Both	10	432 900	847	2.81 (1.73-4.55)	1.51 (1.51-1.72)	<.001	92 (88-95)	0.61-12.91	<.001	Low (suggestive)
Male	5	316 851	NA	2.49 (1.36-4.56)	1.45 (1.25-1.67)	.003	97 (96-98)	0.23-26.59	<.001	Low (suggestive)
Female	5	114 816	NA	2.98 (1.46-6.10)	1.82 (1.32-2.51)	.003	92 (85-96)	0.20-44.17	<.001	Low (suggestive)
Neoplasms, ASD both	2	40 233	109	2.05 (1.46-2.87)	1.80 (1.46-2.23)	<.001	53 (NA)	NA	.14	Low (suggestive)
Nervous system, ASD both	4	61 066	102	10.79 (5.42-21.10)	7.49 (5.78-9.72)	<.001	85 (64-94)	0.52-219.57	<.001	Low (suggestive)
Respiratory system, ASD both	2	40 233	62	3.87 (1.80-8.32)	2.68 (1.99-3.62)	.001	86 (NA)	NA	.007	Low (suggestive)
Digestive system, ASD both	2	40 233	40	4.59 (2.29-9.19)	3.31 (2.25-4.87)	<.001	76 (NA)	NA	.0	Low (suggestive)
Congenital malformations, ASD both	2	40 233	37	11.74 (4.49-30.74)	19.10 (11.94-30.55)	<.001	87 (NA)	NA	.005	Low (suggestive)

Abbreviations: ADHD, attention-deficit/hyperactivity disorder; ASD, autism spectrum disorder; NA, not available; RR, risk ratio.

^a^ Outcomes with at least 2 studies for meta-analysis. Predictive intervals were inestimable with less than 3 studies.

Secondary outcome meta-analyses focused on the cause-specific mortality RR in persons with ASD or ADHD compared with the general population and included 17 studies^13,16,17,19,21,33,34,35,36,37,38,39,42,44,45,46,47,49,51,52,54,56^ that contributed 89 data points (Table 2 and eTable 9 and eFigures 1-7 in the Supplement). A total of 67 of 87 cause-specific mortality RR estimates (75%) were increased. Among persons with ASD, deaths from natural causes (4 studies^13,34,35,47,49,54^; RR, 3.80; 95% CI, 2.06-7.01; *I^2^*, 96%; low confidence) and deaths from unnatural causes were increased (6 studies^13,34,35,47,49,50,54,56^; RR, 2.50; 95% CI, 1.49-4.18; *I^2^*, 95%; low confidence). Among persons with ADHD, deaths from natural causes were not significantly increased (4 studies^16,17,19,44,45^; RR, 1.62; 95% CI, 0.89-2.96; *I^2^*, 88%; low confidence), but deaths from unnatural causes were higher than expected (10 studies^16,17,19,33,36,37,42,44,45,46,51,52^; RR, 2.81; 95% CI, 1.73-4.55; *I^2^*, 92%; low confidence).

### Additional Analyses

The full details of the additional analyses are reported in eFigures 1 to 23 in the Supplement. Statistical heterogeneity was not substantially reduced when analyses were stratified by prespecified (exploratory) subgroup analyses such as number of comorbidities, age group at first diagnosis, number of participants (sample size), setting, follow-up period, study quality, and adjustment for potential confounders. Overall, the *I^2^* statistic indicated that data were heterogeneous in many of the pooled analyses, and therefore, these summary measures must be interpreted with appropriate caution. Insufficient data were available to do preplanned subgroup analyses^24^ by diagnostic criteria, ethnicity, geographic region, or year of publication as there were not enough data. We undertook a funnel plot, and Begg and Egger tests of the 12 studies examining all-cause mortality in people with ASD, with results showing no clear evidence of small study effects (eFigure 22 in the Supplement).

## Discussion

This systematic review and meta-analysis comprehensively assess for the first time, to our knowledge, the available evidence regarding the risk of mortality in persons with ASD or ADHD. We included 27 epidemiologic studies in our quantitative evaluation, 16 of which were judged to be at low risk of bias. We found that ASD and ADHD are associated with a significantly increased risk of all-cause mortality. However, the results should be interpreted with caution because there was evidence of heterogeneity between study estimates of the mortality risks. When we examined causes of death, ASD and ADHD were associated with higher mortality risk due to unnatural (external) causes, and only persons with ASD had an increased risk of mortality from natural causes of death, but the evidence was judged as only low confidence. Fewer studies exist examining the risk of mortality among first-degree relatives of persons with ASD or ADHD, to our knowledge.

Several mechanisms, including health determinants and biological pathways, have been suggested as potential factors that might explain the excess premature mortality among children and young persons with ASD or ADHD. However, establishing a causal relationship is difficult because the associations between mortality and childhood-onset developmental disorders are complex. Severe mental and behavioral disorders appear to be associated with reduced life expectancy, both in terms of mortality from external causes and mortality from other medical conditions or diseases.^10,11^ Findings from previous studies and reviews have suggested that children and adults with ASD/ADHD are associated with coexisting mental and neurologic conditions (such as oppositional and conduct disorders, tic disorders, epilepsy, depression, anxiety, and substance use disorders).^3,4,5,57^ As children and young persons with ASD/ADHD age, they often experience emotional and social difficulties.^3,4,5^ Some people also exhibit impulsive forms of behavior with negative impacts on their quality of life. Behaviors such as impulsivity and/or inattention can be contributing factors for injuries and unintentional incidents in children with ASD/ADHD.^58,59^

Previous studies^16,17,49^ have tested potential modifying effects of comorbidity on mortality risk for persons with ASD/ADHD. For example, Dalsgaard et al,^16^ Sun et al,^17^ and Schendel et al^49^ suggested increased mortality RRs in persons with ADHD or ASD with comorbid neurologic or mental conditions (eg, oppositional defiant disorder, conduct disorder, or substance use disorder). However, it should be noted that presence of ASD and/or ADHD with any distinct additional medical condition (the so-called comorbidity or multimorbidity) may be confounded by previous exposures (such as socioeconomic factors, environmental factors, and childhood abuse).^60^ Prevention efforts to reduce mortality in persons with ASD or ADHD may need to address the conditions that appear to mediate causes of death. While potentially preventable, reducing excess premature mortality (eg, due to external causes) can be challenging. For example, systematic screening would be advisable in health services and social care, and preventive education can be feasible in almost all circumstances. However, some persons with ADHD/ASD are often from socioeconomically disadvantaged groups/areas and are more likely to be exposed to environmental risk factors (eg, substance misuse, violence) than other populations.^61,62,63^ Similarly, studies^64,65^ have shown that persons with ADHD or ASD may be less likely to receive timely diagnosis and prompt care. All these factors are possible complications of ASD/ADHD, so the implication for practice is that health care professionals should recognize their importance.

### Strengths and Limitations

This study had several strengths and limitations. We used established methods to leverage a large number of records identified from each database searched and contacted study authors to identify relevant outcome data. However, our search strategy could have omitted abstracts that did not state all-cause or cause-specific mortality as an included outcome. Observational studies in this area are increasingly conducted within large routinely collected electronic health databases, sometimes with hundreds of thousands of health care users. These studies generate precise estimates of exposure effects, which sometimes may be inaccurate because of potential residual confounding. For example, electronic health records data may have limitations when used for research, including limited information on lifestyle and socioeconomic determinants of health. In this systematic review, there was heterogeneity in the included studies in terms of the design, settings, populations, and outcomes. Some of the included studies have a restricted age of follow-up (eg, individuals younger than 40 years). We used study-level data instead of individual participant data, so the small number of studies (and events) for most cause-specific mortality estimates limited the subgroup analyses that could be conducted to account for potential sources of heterogeneity. Furthermore, our results might have limited generalizability because the studies were mostly conducted in Western countries. Although we adopted reproducible definitions, our meta-analyses were challenged by the reporting of events in studies. None of the primary studies examined and reported all the fatal outcomes of interest. Also, incomplete reporting is common in the literature and limited our possibilities to optimally synthesize the data for some specific causes of death. Future studies should adopt rigorous protocols when studying the effects of ASD/ADHD on health, which should clearly define all outcomes and exposures, how the study population was selected, how data were collected, and that all relevant potential confounders should be accounted for. Reporting guidelines, such as the Strengthening the Reporting of Observational Studies in Epidemiology (STROBE) reporting guideline,^66^ and the Reporting of Studies Conducted Using Observational Routinely-Collected health Data (RECORD) statement,^67^ should be rigorously implemented for individual study reports. Finally, small study effects were not quantitatively assessed for most of the outcomes because there were inadequate numbers of included studies to properly assess a funnel plot or use more sophisticated methods.

## Conclusions

We found suggestive evidence that ASD and ADHD are associated with a significantly increased risk of mortality. Understanding the mechanisms of these associations may lead to targeted strategies to prevent avoidable deaths in high-risk groups of children and young people as an approach to improve public health. For example, clinicians and health care professionals can be encouraged to routinely collect information on behavioral, medical conditions, and health outcomes related to ASD/ADHD, emphasizing the need to recognize and address modifiable vulnerability factors and prevent delays in health care provision. Additionally, we hope that these estimates can shed some light for future studies related to examining mortality-related health estimates in persons with ASD or ADHD.

## Notes

Supplement. eTable 1. PRISMA 2020 checklist eTable 2. MOOSE checklist eTable 3. Coding and classification of causes of death eTable 4. Methods clarifications/modifications from the protocol eTable 5. Search strategy eTable 6. List of excluded studies eTable 7. List of studies with authors contacted for additional clarifications or data eTable 8. Main characteristics of included studies eTable 9. Main results of individual studies eTable 10. Methodological quality assessment of included studies (Newcastle-Ottawa Scale eTable 11. Grading certainty (or credibility) of evidence for summary estimates eTable 12. Summary statistics of risk of cancer in people with anorexia nervosa eFigure 1. Secondary outcome. Unnatural causes of death among ASD/ADHD people eFigure 2. Secondary outcome. Natural causes of death among ASD/ADHD people eFigure 3. Secondary outcome. Deaths caused by nervous system disorders among ASD people eFigure 4. Secondary outcome. Deaths caused by neoplasms among ASD people eFigure 5. Secondary outcome. Deaths caused by respiratory system diseases among ASD people eFigure 6. Secondary outcome. Deaths caused by digestive system diseases among ASD people eFigure 7. Secondary outcome. Deaths caused by congenital malformations among ASD people eFigure 8. Subgroup analyses. ASD comorbidity and all-cause mortality among ASD people eFigure 9. Subgroup analyses. ADHD comorbidity and all-cause mortality among ADHD people eFigure 10. Subgroup analyses. Age group at first diagnosis and all-cause mortality among ASD people eFigure 11. Subgroup analyses. Age group at first diagnosis and all-cause mortality among ADHD people eFigure 12. Subgroup analyses. Cohort/sample size and and all-cause mortality among ASD people eFigure 13. Subgroup analyses. Cohort/sample size and and all-cause mortality among ADHD people eFigure 14. Subgroup analyses. Setting and all-cause mortality among ASD people eFigure 15. Subgroup analyses. Setting and all-cause mortality among ADHD people eFigure 16. Subgroup analyses. Follow-up (in years) and all-cause mortality among ASD people eFigure 17. Subgroup analyses. Follow-up (in years) and all-cause mortality among ADHD people eFigure 18. Subgroup analyses. Study-quality/risk of bias and all-cause mortality among ASD people eFigure 19. Subgroup analyses. Study-quality/risk of bias and all-cause mortality among ADHD people eFigure 20. Subgroup analyses. Adjustment for potential confounders and all-cause mortality among ASD people eFigure 21. Subgroup analyses. Adjustment for potential confounders and all-cause mortality among ADHD people eFigure 22. Funnel plot for primary outcome of all-cause mortality in people with ASD (12 studies) eFigure 23. Treemap summarizing the amount of data according to specific causes of death
