# Supplementary material for: Mortality in Persons With Autism Spectrum Disorder or Attention-Deficit/Hyperactivity Disorder: A Systematic Review and Meta-analysis
Source: JAMA Pediatr. 2022 Feb 14;176(4):e216401. doi: 10.1001/jamapediatrics.2021.6401 (PMC8845021; doi:10.1001/jamapediatrics.2021.6401)
Supplement: Supplement. — eTable 1. PRISMA 2020 checklist eTable 2. MOOSE checklist eTable 3. Coding and classification of causes of death eTable 4. Methods clarifications/modifications from the protocol eTable 5. Search strategy eTable 6. List of excluded studies eTable 7. List of studies with authors contacted for additional clarifications or data eTable 8. Main characteristics of included studies eTable 9. Main results of individual studies eTable 10. Methodological quality assessment of included studies (Newcastle-Ottawa Scale eTable 11. Grading certainty (or credibility) of evidence for summary estimates eTable 12. Summary statistics of risk of cancer in people with anorexia nervosa eFigure 1. Secondary outcome. Unnatural causes of death among ASD/ADHD people eFigure 2. Secondary outcome. Natural causes of death among ASD/ADHD people eFigure 3. Secondary outcome. Deaths caused by nervous system disorders among ASD people eFigure 4. Secondary outcome. Deaths caused by neoplasms among ASD people eFigure 5. Secondary outcome. Deaths caused by respiratory system diseases among ASD people eFigure 6. Secondary outcome. Deaths caused by digestive system diseases among ASD people eFigure 7. Secondary outcome. Deaths caused by congenital malformations among ASD people eFigure 8. Subgroup analyses. ASD comorbidity and all-cause mortality among ASD people eFigure 9. Subgroup analyses. ADHD comorbidity and all-cause mortality among ADHD people eFigure 10. Subgroup analyses. Age group at first diagnosis and all-cause mortality among ASD people eFigure 11. Subgroup analyses. Age group at first diagnosis and all-cause mortality among ADHD people eFigure 12. Subgroup analyses. Cohort/sample size and and all-cause mortality among ASD people eFigure 13. Subgroup analyses. Cohort/sample size and and all-cause mortality among ADHD people eFigure 14. Subgroup analyses. Setting and all-cause mortality among ASD people eFigure 15. Subgroup analyses. Setting and all-cause mortality among ADHD people eFigure 16 [file jamapediatr-e216401-s001.pdf]

Supplemental Online Content

Catalá-López F, Hutton B, Page MJ, et al. Mortality in persons with autism spectrum disorder or attention-deficit/hyperactivity disorder: a systematic review and meta-analysis. *JAMA Pediatr.* Published online February 14, 2022. doi:10.1001/jamapediatrics.2021.6401

eTable 1. PRISMA 2020 checklist

eTable 2. MOOSE checklist

eTable 3. Coding and classification of causes of death

eTable 4. Methods clarifications/modifications from the protocol

eTable 5. Search strategy

eTable 6. List of excluded studies

eTable 7. List of studies where authors were contacted for additional clarifications or data

eTable 8. Main characteristics of included studies

eTable 9. Main results of individual studies

eTable 10. Methodological quality assessment of included studies (Newcastle-Ottawa Scale)

eTable 11. Grading certainty (or credibility) of evidence for summary estimates.

eTable 12. Summary statistics of risk of mortality in people with ASD/ADHD people

eFigure 1. Secondary outcome. Unnatural causes of death among ASD/ADHD people

eFigure 2. Secondary outcome. Natural causes of death among ASD/ADHD people

eFigure 3. Secondary outcome. Deaths caused by nervous system disorders among ASD people

eFigure 4. Secondary outcome. Deaths caused by neoplasms among ASD people

eFigure 5. Secondary outcome. Deaths caused by respiratory system diseases among ASD people

eFigure 6. Secondary outcome. Deaths caused by digestive system disorders among ASD people

eFigure 7. Secondary outcome. Deaths caused by congenital malformations among ASD people

eFigure 8. Subgroup analyses. ASD comorbidity and all-cause mortality among ASD people

eFigure 9. Subgroup analyses. ADHD comorbidity and all-cause mortality among ADHD people

© 2022 Catalá-López F et al. *JAMA Pediatrics*

eFigure 10. Subgroup analyses. Age group at first diagnosis and all-cause mortality among ASD people

eFigure 11. Subgroup analyses. Age group at first diagnosis and all-cause mortality among ADHD people

eFigure 12. Subgroup analyses. Cohort/sample size and all-cause mortality among ASD people

eFigure 13. Subgroup analyses. Cohort/sample size and all-cause mortality among ADHD people

eFigure 14. Subgroup analyses. Setting and all-cause mortality among ASD people

eFigure 15. Subgroup analyses. Setting and all-cause mortality among ADHD people

eFigure 16. Subgroup analyses. Follow-up (in years) and all-cause mortality among ASD people

eFigure 17. Subgroup analyses. Follow-up (in years) and all-cause mortality among ADHD people

eFigure 18. Subgroup analyses. Study quality/risk of bias and all-cause mortality among ASD people

eFigure 19. Subgroup analyses. Study quality/risk of bias and all-cause mortality among ADHD people

eFigure 20. Subgroup analyses. Adjustment for potential confounders and all-cause mortality among ASD people

eFigure 21. Subgroup analyses. Adjustment for potential confounders and all-cause mortality among ADHD people

eFigure 22. Funnel plot for primary outcome of all-cause mortality in people with ASD

eFigure 23. Treemap summarizing the amount of data according to specific causes of death

This supplemental material has been provided by the authors to give readers additional information about their work.

© 2022 Catalá-López F et al. *JAMA Pediatrics*

eTable 1. PRISMA 2020 checklist.

Section and Topic	Item #	Checklist item	Location where item is reported
**TITLE**			
Title	1	Identify the report as a systematic review.	1
**ABSTRACT**			
Abstract	2	See the PRISMA 2020 for Abstracts checklist.	3 and 4
**INTRODUCTION**			
Rationale	3	Describe the rationale for the review in the context of existing knowledge.	7
Objectives	4	Provide an explicit statement of the objective(s) or question(s) the review addresses.	8
**METHODS**			
Eligibility criteria	5	Specify the inclusion and exclusion criteria for the review and how studies were grouped for the syntheses.	9
Information sources	6	Specify all databases, registers, websites, organisations, reference lists and other sources searched or consulted to identify studies. Specify the date when each source was last searched or consulted.	8 and 9
Search strategy	7	Present the full search strategies for all databases, registers and websites, including any filters and limits used.	8 and 9, and eTable 5
Selection process	8	Specify the methods used to decide whether a study met the inclusion criteria of the review, including how many reviewers screened each record and each report retrieved, whether they worked independently, and if applicable, details of automation tools used in the process.	10
Data collection process	9	Specify the methods used to collect data from reports, including how many reviewers collected data from each report, whether they worked independently, any processes for obtaining or confirming data from study investigators, and if applicable, details of automation tools used in the process.	10
Data items	10a	List and define all outcomes for which data were sought. Specify whether all results that were compatible with each outcome domain in each study were sought (e.g. for all measures, time points, analyses), and if not, the methods used to decide which results to collect.	10 and 11
	10b	List and define all other variables for which data were sought (e.g. participant and intervention characteristics, funding sources). Describe any assumptions made about any missing or unclear information.	10 and 11
Study risk of bias assessment	11	Specify the methods used to assess risk of bias in the included studies, including details of the tool(s) used, how many reviewers assessed each study and whether they worked independently, and if applicable, details of automation tools used in the process.	10
Effect measures	12	Specify for each outcome the effect measure(s) (e.g. risk ratio, mean difference) used in the synthesis or presentation of results.	11
Synthesis methods	13a	Describe the processes used to decide which studies were eligible for each synthesis (e.g. tabulating the study intervention characteristics and comparing against the planned groups for each synthesis (item #5)).	11
	13b	Describe any methods required to prepare the data for presentation or synthesis, such as handling of missing summary statistics, or data conversions.	11, and eTables 8
	13c	Describe any methods used to tabulate or visually display results of individual studies and syntheses.	11, and Fig. 2, 3 and 4, and eFigures 1-22
	13d	Describe any methods used to synthesize results and provide a rationale for the choice(s). If meta-analysis was performed, describe the model(s), method(s) to identify the presence and extent of statistical heterogeneity, and software package(s) used.	11
	13e	Describe any methods used to explore possible causes of heterogeneity among study results (e.g. subgroup analysis, meta-regression).	11
	13f	Describe any sensitivity analyses conducted to assess robustness of the synthesized results.	11

© 2022 Catalá-López F et al. *JAMA Pediatrics*

Section and Topic	Item #	Checklist item	Location where item is reported
Reporting bias assessment	14	Describe any methods used to assess risk of bias due to missing results in a synthesis (arising from reporting biases).	11
Certainty assessment	15	Describe any methods used to assess certainty (or confidence) in the body of evidence for an outcome.	12
**RESULTS**			
Study selection	16a	Describe the results of the search and selection process, from the number of records identified in the search to the number of studies included in the review, ideally using a flow diagram.	13 and 14, and Fig. 1
	16b	Cite studies that might appear to meet the inclusion criteria, but which were excluded, and explain why they were excluded.	eTable 6
Study characteristics	17	Cite each included study and present its characteristics.	13, Table 1, and eTable 8 and 9
Risk of bias in studies	18	Present assessments of risk of bias for each included study.	eTable 10
Results of individual studies	19	For all outcomes, present, for each study: (a) summary statistics for each group (where appropriate) and (b) an effect estimate and its precision (e.g. confidence/credible interval), ideally using structured tables or plots.	Figures 3 and 4, and eFigures
Results of syntheses	20a	For each synthesis, briefly summarise the characteristics and risk of bias among contributing studies.	14, Table 2, and eTable 12
	20b	Present results of all statistical syntheses conducted. If meta-analysis was done, present for each the summary estimate and its precision (e.g. confidence/credible interval) and measures of statistical heterogeneity. If comparing groups, describe the direction of the effect.	14 and 15, Table 2, Figures 3 and 4, and eFigures 1-21
	20c	Present results of all investigations of possible causes of heterogeneity among study results.	15, and eFigures 1-21
	20d	Present results of all sensitivity analyses conducted to assess the robustness of the synthesized results.	15, and eFigures
Reporting biases	21	Present assessments of risk of bias due to missing results (arising from reporting biases) for each synthesis assessed.	eFigure 22
Certainty of evidence	22	Present assessments of certainty (or confidence) in the body of evidence for each outcome assessed.	eTable 10
**DISCUSSION**			
Discussion	23a	Provide a general interpretation of the results in the context of other evidence.	15 and 16
	23b	Discuss any limitations of the evidence included in the review.	17 and 18
	23c	Discuss any limitations of the review processes used.	18
	23d	Discuss implications of the results for practice, policy, and future research.	18 and 19
**OTHER INFORMATION**			
Registration and protocol	24a	Provide registration information for the review, including register name and registration number, or state that the review was not registered.	8
	24b	Indicate where the review protocol can be accessed, or state that a protocol was not prepared.	8
	24c	Describe and explain any amendments to information provided at registration or in the protocol.	eTable 4
Support	25	Describe sources of financial or non-financial support for the review, and the role of the funders or sponsors in the review.	19 and 20
Competing interests	26	Declare any competing interests of review authors.	20 (electronic forms)
Availability of data, code and other materials	27	Report which of the following are publicly available and where they can be found: template data collection forms; data extracted from included studies; data used for all analyses; analytic code; any other materials used in the review.	12

© 2022 Catalá-López F et al. *JAMA Pediatrics*

Page MJ, McKenzie JE, Bossuyt PM, Boutron I, Hoffmann TC, Mulrow CD, et al. The PRISMA 2020 statement: an updated guideline for reporting systematic reviews. BMJ 2021;372:n71. doi: 10.1136/bmj.n71. For more information, visit: http://www.prisma-statement.org/

eTable 2. MOOSE checklist.

Section/Topic	MOOSE checklist Item(s)	Reported on Page #
**BACKGROUND**		
Reporting of background should include	Problem definition. Hypothesis statement. Description of study outcome(s). Type of exposure or intervention used. Type of study designs used. Study population.	7
**SEARCH**		
Reporting of search strategy should include	Qualifications of searchers (eg, librarians and investigators). Search strategy, including time period included in the synthesis and keywords. Effort to include all available studies, including contact with authors. Databases and registries searched. Search software used, name and version, including special features used (eg, explosion). Use of hand searching (eg, reference lists of obtained articles). List of citations located and those excluded, including justification. Method of addressing articles published in languages other than English. Method of handling abstracts and unpublished studies. Description of any contact with authors.	9, and eTable 5
**METHODS**		
Reporting of methods should include	Description of relevance or appropriateness of studies assembled for assessing the hypothesis to be tested. Rationale for the selection and coding of data (eg, sound clinical principles or convenience). Documentation of how data were classified and coded (eg, multiple raters, blinding, and interrater reliability). Assessment of confounding (eg, comparability of cases and controls in studies where appropriate). Assessment of study quality, including blinding of quality assessors; stratification or regression on possible predictors of study results. Assessment of heterogeneity. Description of statistical methods (eg, complete description of fixed or random effects models, justification of whether the chosen models account for predictors of study results, dose-response models, or cumulative meta-analysis) in sufficient detail to be replicated. Provision of appropriate tables and graphics.	8-12
**RESULTS**		
Reporting of results should include	Graphic summarizing individual study estimates and overall estimate. Table giving descriptive information for each study included. Results of sensitivity testing (eg, subgroup analysis). Indication of statistical uncertainty of findings.	12-15, Tables 1 and 2, Figures 2-4, and eTables 8-11, and eFigures 1-22
**DISCUSSION**		
Reporting of discussion should include	Quantitative assessment of bias (eg, publication bias). Justification for exclusion (eg, exclusion of non–English-language citations). Assessment of quality of included studies.	15-19
**CONCLUSIONS**		
Reporting of conclusions should include	Consideration of alternative explanations for observed results. Generalization of the conclusions (ie, appropriate for the data presented and within the domain of the literature review). Guidelines for future research. Disclosure of funding source.	19, 20

Stroup DF, Berlin JA, Morton SC, Olkin I, Williamson GD, Rennie D, Moher D, Becker BJ, Sipe TA, Thacker SB. Meta-analysis of observational studies in epidemiology: a proposal for reporting. Meta-analysis Of Observational Studies in Epidemiology (MOOSE) group. JAMA. 2000 Apr 19;283(15):2008-12. Review. PubMed PMID: 10789670

© 2022 Catalá-López F et al. *JAMA Pediatrics*

eTable 3. Coding and classification of causes of death.

Causes of death	ICD-10 code	ICD-9 code	ICD-8 code
All causes of death	A00-Y98	001-E999	000-E999
Infectious and parasitic diseases	A00-B99	001-033,34-134,136-139	000-033, 34-139
Neoplasms	C00-D48	140-175, 179-239	140-239
Diseases of the blood and blood-forming organs	D50-D89	279-289	135,280-289
Endocrine, nutritional and metabolic diseases	E00-E90	240-278	240-279
Mental and behavioural disorders	F00-F99	290-319	290-315
Diseases of the nervous system	G00-H93	320-389	320-389
Diseases of the circulatory system	I00-I99	390-459	390-4358
Diseases of the respiratory system	J00-J98	460-519	460-519
Diseases of the digestive system	K00-K92	520-579	520-543, 550-577
Diseases of the skin and subcutaneous tissue	L00-L99	680-709	680-709
Diseases of the musculoskeletal system/connective tissue	M00-M99	710-739	710-738
Diseases of the genitourinary system	N00-N98	580-629	580-629
Complications of pregnancy, childbirth and puerperium	O00-O99	630-676	630-678
Certain conditions originating in the perinatal period	P00-P96	760-779	760-778
Congenital malformations and chromosomal abnormalities	Q00-Q99	740-759	740-759
Symptoms, signs, ill-defined causes	R00-R99	780-799	780-796
External causes of mortality	V01-Y98	E800-E999	E800-E999

© 2022 Catalá-López F et al. *JAMA Pediatrics*

eTable 4. Methods clarifications/modifications from the protocol

The protocol for this meta-analysis has been registered in PROSPERO (No. CRD42017059955), and published in the open-access journal *Systematic Reviews*, available at: https://systematicreviewsjournal.biomedcentral.com/articles/10.1186/s13643-017-0581-9
**Clarification 1: Additional analyses**<br>Page 4 of the published protocol: "*If sufficient studies are identified, we will present subgroup analyses to attempt to explain any potential observed between-study heterogeneity. The potential moderators (covariates) considered will be gender (male or female), diagnostic criteria used (e.g., DSM or ICD and their versions), age group at first diagnosis (children and adolescents [< 18 years old] versus adults [≥ 18 years old]), ethnicity (e.g., white or non-white), number of comorbidities (e.g., 0, 1, 2, or ≥3), medical or neuropsychiatric comorbidity (e.g., presence or absence of a comorbid disorder with ASD or ADHD), index subjects (patients, parents of patients, or siblings of patients), country or geographic region, cohort/sample size (< 500, 500–1000, or > 1000 participants), setting (mixed, inpatient, outpatient, or community/general population), population-based (yes or no), follow-up period (0–1, > 1–5, or > 5 years), year of publication as a proxy of changes in clinical practice over time (before 2000 or in 2000 and after), study quality (low or high-moderate quality), and effect measure adjusted for potential confounders (age, sex, or other).*"<br><br>Clarification: Pre-planned subgroup analyses (such as diagnostic criteria, ethnicity, index subjects, country or geographic region, population-based(Y/N) and year of publication) could not be carried out for pooled analyses as there were not enough data for establishing well-balanced subgroups. For the primary outcome of all-cause mortality among ASD people, we conducted the following pre-planned (exploratory) subgroup analyses: number of comorbities, age group at first diagnosis, cohort/sample size, setting, follow-up period, study quality and adjustment for potential confounders. For the primary outcome of all-cause mortality among ASD people, we conducted the following pre-planned (exploratory) subgroup analyses: number of comorbities.<br><br>Page 4 of the published protocol: "*To further assess the consistency of evidence over time, we will perform cumulative meta-analysis in the order of publication year* (…)".<br><br>Clarification: Given the number of individual study estimates available per outcome, cumulative meta-analysis was considered not feasible.
**Clarification 2: Additional analysis**<br>Page 5 of the published protocol: "*Small study effects will be investigated by visual inspection of funnel plots (where appropriate) and with Egger's test and Begg's test, with the results considered to indicate potential small study effects when* P *< 0.10.*"<br><br>Clarification: Given the number of individual study estimates available per outcome (less than 10 studies), small study effects/publication bias assessment (e.g. funnel plots, Begg and Egger test) was considered not feasible.<br>We only assessed small study effects/publication bias using for the primary outcome of all-cause mortality in ASD people (12 studies): Begg test (P = 0.681), and Egger test (P = 0.248). See funnel plot in eFig16.

© 2022 Catalá-López F et al. *JAMA Pediatrics*

eTable 5. Search strategy.

eTable 5a. Key terms for **PubMed/MEDLINE** search.

Search	Query	Items
#1	(((("Autistic Disorder"[Mesh] AND "Autism Spectrum Disorder"[Mesh]) OR "Asperger Syndrome"[Mesh]) OR "Attention Deficit Disorder with Hyperactivity"[Mesh]) OR "Child Development Disorders, Pervasive"[Mesh]) OR "Hyperkinesis"[Mesh]	69199
#2	autism OR autistic OR pervasive developmental disorder* OR Asperger OR attention deficit disorder with hyperactivity OR attention deficit/hyperactivity disorder OR adhd OR inattent* OR impulsivity OR hyperkinesis OR hyperkinetic*	169702
#3	epidemiology OR epidemiologic* OR cohort stud* OR longitudinal stud* OR case-control stud*	4651206
#4	mortality OR death* OR survival* OR fatal*	2831878
#5	#1 OR #2	170011
#6	#3 AND #4 AND #5	**1636**

eTable 5b. Key terms for **SCOPUS** search.

Search	Query	Items
#1	TITLE-ABS-KEY (autism* OR autistic*)	89961
#2	TITLE-ABS-KEY ("pervasive development* disorder*")	3901
#3	TITLE-ABS-KEY (impulsivity)	18325
#4	TITLE-ABS-KEY (inattent*)	12688
#5	TITLE-ABS-KEY (hyperkine*)	10970
#6	TITLE-ABS-KEY (asperger)	6277
#7	(TITLE-ABS-KEY ("attention deficit and hyperactivity disorder*" OR "attention deficit disorder* and hyperactivity" OR "attention deficit disorder* hyperactivity" OR "attention deficit disorder* with hyperactivity" OR "attention deficit hyperactivity disorder*") OR TITLE-ABS-KEY ("attention deficit hyperactivity" OR "attention deficit with hyperactivity disorder*" OR "deficit hyperactivity disorder*" OR adhd OR ("attention deficit" AND "Hyperactivity disorder*")))	50857
#8	(TITLE-ABS-KEY (autism* OR autistic*)) OR (TITLE-ABS-KEY ("pervasive development* disorder*")) OR (TITLE-ABS-KEY (impulsivity)) OR (TITLE-ABS-KEY (inattent*)) OR (TITLE-ABS-KEY (hyperkine*)) OR (TITLE-ABS-KEY (asperger)) OR ((TITLE-ABS-KEY ("attention deficit and hyperactivity disorder*" OR "attention deficit disorder* and hyperactivity" OR "attention deficit disorder* hyperactivity" OR "attention deficit disorder* with hyperactivity" OR "attention deficit hyperactivity disorder*") OR TITLE-ABS-KEY ("attention deficit hyperactivity" OR "attention deficit with hyperactivity disorder*" OR "deficit hyperactivity disorder*" OR adhd OR ("attention deficit" AND "Hyperactivity disorder*"))))	166989
#9	TITLE-ABS-KEY (epidemiology OR epidemiologic* OR "cohort stud*" OR "longitudinal stud*" OR "case-control stud*")	1689432
#10	TITLE-ABS-KEY (mortality OR death* OR survival* OR fatal*)	3929799
#11	((TITLE-ABS-KEY (autism* OR autistic*)) OR (TITLE-ABS-KEY ("pervasive development* disorder*")) OR (TITLE-ABS-KEY (impulsivity)) OR (TITLE-ABS-KEY (inattent*)) OR (TITLE-ABS-KEY (hyperkine*)) OR (TITLE-ABS-KEY (asperger)) OR ((TITLE-ABS-KEY ("attention deficit and hyperactivity disorder*" OR "attention deficit disorder* and hyperactivity" OR "attention deficit disorder* hyperactivity" OR "attention deficit disorder* with hyperactivity" OR "attention deficit hyperactivity disorder*") OR TITLE-ABS-KEY ("attention deficit hyperactivity" OR "attention deficit with hyperactivity disorder*" OR "deficit hyperactivity disorder*" OR adhd OR ("attention deficit" AND "Hyperactivity disorder*"))))) AND (TITLE-ABS-KEY (epidemiology OR epidemiologic* OR "cohort stud*" OR "longitudinal stud*" OR "case-control stud*")) AND (TITLE-ABS-KEY (mortality OR death* OR survival* OR fatal*))	**634**

eTable 5c. Key terms for **Embase** search.

Search	Query	Items
#1	autism* OR autistic*	88351
#2	'pervasive development* disorder*'	3923
#3	impulsivity	19121
#4	inattent*	10970
#5	hyperkine*	10651
#6	asperger	5864
#7	'attention deficit and hyperactivity disorder*' OR 'attention deficit disorder* and hyperactivity' OR 'attention deficit disorder* hyperactivity' OR 'attention deficit disorder* with hyperactivity' OR 'attention deficit hyperactivity disorder*' OR 'attention deficit hyperactivity' OR 'attention deficit with hyperactivity disorder*' OR 'deficit hyperactivity disorder*' OR adhd OR ('attention deficit' AND 'hyperactivity disorder*')	48651
#8	#1 OR #2 OR #3 OR #4 OR #5 OR #6 OR #7	159745
#9	epidemiology OR epidemiologic* OR 'cohort stud*' OR 'longitudinal stud*' OR 'case-control stud*'	27222542
#10	mortality OR death* OR survival* OR fatal*	4116473
#11	#8 AND #9 AND #10	**1087**

© 2022 Catalá-López F et al. *JAMA Pediatrics*

eTable 5d. Key terms for **Web of Science** Core collection search.

Search	Query	Items
#1	TOPIC: (Autism* or Autistic*)	72294
#2	TOPIC: ("pervasive development* disorder*")	4599
#3	TOPIC: (impulsivity)	19643
#4	TOPIC: (Inattent*)	9758
#5	TOPIC: (hyperkine*)	3953
#6	TOPIC: (Asperger)	6446
#7	TOPIC: ("attention deficit and hyperactivity disorder*" or "attention deficit disorder* and hyperactivity" or "attention deficit disorder* hyperactivity" or "attention deficit disorder* with hyperactivity" or "attention deficit hyperactivity disorder*" or "attention deficit hyperactivity" or "attention deficit with hyperactivity disorder*" or "deficit hyperactivity disorder*" or adhd or ("attention deficit" and "Hyperactivity disorder*"))	49034
#8	#7 OR #6 OR #5 OR #4 OR #3 OR #2 OR #1	144324
#9	TOPIC: (epidemiology or epidemiologic* or "cohort stud*" or "longitudinal stud*" or "case-control stud*")	903816
#10	TOPIC: (mortality or death* or survival* or fatal*)	2731829
#11	#10 AND #9 AND #8	**443**

eTable 5e. Key terms for **PsycINFO** search.

Search	Query	Items
S1	Autism* or Autistic*	83493
S2	"pervasive development* disorder*"	3500
S3	impulsivity	17648
S4	Inattent*	8833
S5	hyperkine*	7730
S6	Asperger	4646
S7	"attention deficit and hyperactivity disorder*" or "attention deficit disorder* and hyperactivity" or "attention deficit disorder* hyperactivity" or "attention deficit disorder* with hyperactivity" or "attention deficit hyperactivity disorder*" or "attention deficit hyperactivity" or "attention deficit with hyperactivity disorder*" or "deficit hyperactivity disorder*" or adhd or ("attention deficit" and "Hyperactivity disorder*")	40328
S8	S1 OR S2 OR S3 OR S4 OR S6 OR S7	128749
S9	epidemiology or epidemiologic* or "cohort stud*" or "longitudinal stud*" or "case-control stud*"	382731
S10	mortality or death* or survival* or fatal*	181733
S11	S8 AND S9 AND S10	**400**

© 2022 Catalá-López F et al. *JAMA Pediatrics*

eTable 6. List of excluded studies.

Reference	Cause
Kanner L. Follow-up study of eleven autistic children originally reported in 1943. J Autism Child Schizophr. 1971 Apr-Jun;1(2):119-45. doi: 10.1007/BF01537953. PMID: 5172388.	Wrong study design
Kobayashi R, Murata T, Yoshinaga K. A follow-up study of 201 children with autism in Kyushu and Yamaguchi areas, Japan. J Autism Dev Disord. 1992;22(3):395-411. doi: 10.1007/BF01048242. PMID: 1383189.	Wrong study design
Brent DA, Johnson B, Bartle S, Bridge J, Rather C, Matta J, Connolly J, Constantine D. Personality disorder, tendency to impulsive violence, and suicidal behavior in adolescents. J Am Acad Child Adolesc Psychiatry. 1993;32(1):69-75. doi: 10.1097/00004583-199301000-00010. PMID: 8428886.	Did not have ADHD/autism as an exposure
Boyle CA, Decouflé P, Holmgreen P. Contribution of developmental disabilities to childhood mortality in the United States: a multiple-cause-of-death analysis. Paediatr Perinat Epidemiol. 1994;8(4):411-22. doi: 10.1111/j.1365-3016.1994.tb00480.x. PMID: 7532859.	Data not abstractable
Ballaban-Gil K, Rapin I, Tuchman R, Shinnar S. Longitudinal examination of the behavioral, language, and social changes in a population of adolescents and young adults with autistic disorder. Pediatr Neurol. 1996;15(3):217-23. doi: 10.1016/s0887-8994(96)00219-6. PMID: 8916159.	Wrong study design
Thomsen PH. A 22- to 25-year follow-up study of former child psychiatric patients: a register-based investigation of the course of psychiatric disorder and mortality in 546 Danish child psychiatric patients. Acta Psychiatr Scand. 1996;94(6):397-403. doi: 10.1111/j.1600-0447.1996.tb09880.x. PMID: 9020989.	Did not have ADHD/autism as an exposure
Daley KC. Updates on attention deficit hyperactivity disorder, child abuse and neglect, and sudden infant death syndrome. Curr Opin Pediatr. 2003;15(2):216-25. doi: 10.1097/00008480-200304000-00014. PMID: 12640282.	Wrong study design
Cohen A, Asor E, Tirosh E. Predictive factors of early mortality in children with developmental disabilities: a case-comparison analysis. J Child Neurol. 2008 May;23(5):536-42. doi: 10.1177/0883073807309795. PMID: 18184935.	Wrong study design
Sourander A, Klomek AB, Niemelä S, Haavisto A, Gyllenberg D, Helenius H, Sillanmäki L, Ristkari T, Kumpulainen K, Tamminen T, Moilanen I, Piha J, Almqvist F, Gould MS. Childhood predictors of completed and severe suicide attempts: findings from the Finnish 1981 Birth Cohort Study. Arch Gen Psychiatry. 2009;66(4):398-406. doi: 10.1001/archgenpsychiatry.2009.21. PMID: 19349309.	Did not have ADHD/autism as an exposure
McCarthy S, Cranswick N, Potts L, Taylor E, Wong IC. Mortality associated with attention-deficit hyperactivity disorder (ADHD) drug treatment: a retrospective cohort study of children, adolescents and young adults using the general practice research database. Drug Saf. 2009;32(11):1089-96. doi: 10.2165/11317630-000000000-00000. PMID: 19810780.	Did not have ADHD/autism as an exposure
Bussing R, Mason DM, Bell L, Porter P, Garvan C. Adolescent outcomes of childhood attention-deficit/hyperactivity disorder in a diverse community sample. J Am Acad Child Adolesc Psychiatry. 2010;49(6):595-605. doi: 10.1016/j.jaac.2010.03.006. PMID: 20494269.	Did not have mortality as an outcome
Chronis-Tuscano A, Molina BS, Pelham WE, Applegate B, Dahlke A, Overmyer M, Lahey BB. Very early predictors of adolescent depression and suicide attempts in children with attention-deficit/hyperactivity disorder. Arch Gen Psychiatry. 2010 Oct;67(10):1044-51. doi: 10.1001/archgenpsychiatry.2010.127. PMID: 20921120; PMCID: PMC3382065.	Did not have mortality as an outcome
Pickett J, Xiu E, Tuchman R, Dawson G, Lajonchere C. Mortality in individuals with autism, with and without epilepsy. J Child Neurol. 2011;26(8):932-9. doi: 10.1177/0883073811402203. PMID: 21471551.	Wrong study design
Schelleman H, Bilker WB, Strom BL, Kimmel SE, Newcomb C, Guevara JP, Daniel GW, Cziraky MJ, Hennessy S. Cardiovascular events and death in children exposed and unexposed to ADHD agents. Pediatrics. 2011 Jun;127(6):1102-10. doi: 10.1542/peds.2010-3371. PMID: 21576311.	Did not have ADHD/autism as an exposure
Schelleman H, Bilker WB, Kimmel SE, Daniel GW, Newcomb C, Guevara JP, Cziraky MJ, Strom BL, Hennessy S. Methylphenidate and risk of serious cardiovascular events in adults. Am J Psychiatry. 2012 Feb;169(2):178-85. doi: 10.1176/appi.ajp.2011.11010125. PMID: 22318795.	Did not have ADHD/autism as an exposure
Kang JH, Lin HC, Chung SD. Attention-deficit/hyperactivity disorder increased the risk of injury: a population-based follow-up study. Acta Paediatr. 2013 Jun;102(6):640-3. doi: 10.1111/apa.12213. PMID: 23647526.	Did not have mortality as an outcome
Khan A, Faucett J, Morrison S, Brown WA. Comparative mortality risk in adult patients with schizophrenia, depression, bipolar disorder, anxiety disorders, and attention-deficit/hyperactivity disorder participating in psychopharmacology clinical trials. JAMA Psychiatry. 2013;70(10):1091-9. doi: 10.1001/jamapsychiatry.2013.149. PMID: 23986353.	Wrong study design
Chang Z, Lichtenstein P, D'Onofrio BM, Sjölander A, Larsson H. Serious transport accidents in adults with attention-deficit/hyperactivity disorder and the effect of medication: a population-based study. JAMA Psychiatry. 2014 Mar;71(3):319-25. doi: 10.1001/jamapsychiatry.2013.4174. PMID: 24477798.	Data not abstractable
Chang Z, Quinn PD, Hur K, Gibbons RD, Sjölander A, Larsson H, D'Onofrio BM. Association Between Medication Use for Attention-Deficit/Hyperactivity Disorder and Risk of Motor Vehicle Crashes. JAMA Psychiatry. 2017;74(6):597-603. doi: 10.1001/jamapsychiatry.2017.0659. PMID: 28492937.	Did not have mortality as an outcome
Acar E, Dursun OB, Esin İS, Öğütlü H, Özcan H, Mutlu M. Unintentional Injuries in Preschool Age Children: Is There a Correlation With Parenting Style and Parental Attention Deficit and Hyperactivity Symptoms. Medicine (Baltimore). 2015;94(32):e1378. doi: 10.1097/MD.0000000000001378. PMID: 26266395; PMCID: PMC4616671.	Did not have mortality as an outcome
Chiang HL, Liu CJ, Hu YW, Chen SC, Hu LY, Shen CC, Yeh CM, Chen TJ, Gau SS. Risk of cancer in children, adolescents, and young adults with autistic disorder. J Pediatr. 2015 Feb;166(2):418-23.e1. doi: 10.1016/j.jpeds.2014.10.029. PMID: 25453246.	Data not abstractable
Alasaarela L, Hakko H, Riala K, Riipinen P. Association of Self-reported Impulsivity to Nonsuicidal Self-Injury, Suicidality, and Mortality in Adolescent Psychiatric Inpatients. J Nerv Ment Dis. 2017;205(5):340-345. doi: 10.1097/NMD.0000000000000655. PMID: 28141633.	Wrong study design
Curry AE, Metzger KB, Pfeiffer MR, Elliott MR, Winston FK, Power TJ. Motor Vehicle Crash Risk Among Adolescents and Young Adults With Attention-Deficit/Hyperactivity Disorder. JAMA Pediatr. 2017;171(8):756-763. doi: 10.1001/jamapediatrics.2017.0910. PMID: 28604931.	Did not have mortality as an outcome
Bourke J, Nembhard WN, Wong K, Leonard H. Twenty-Five Year Survival of Children with Intellectual Disability in Western Australia. J Pediatr. 2017;188:232-239.e2. doi: 10.1016/j.jpeds.2017.06.008. PMID: 28705655.	Did not have ADHD/autism as an exposure

© 2022 Catalá-López F et al. *JAMA Pediatrics*

Franklin RC, Pearn JH, Peden AE. Drowning fatalities in childhood: the role of pre-existing medical conditions. Arch Dis Child. 2017;102(10):888-893. doi: 10.1136/archdischild-2017-312684. Epub 2017 May 8. PMID: 28483756.	Wrong study design
Laukkala T, Bor R, Budowle B, Sajantila A, Navathe P, Sainio M, Vuorio A. Attention-Deficit/Hyperactivity Disorder and Fatal Accidents in Aviation Medicine. Aerosp Med Hum Perform. 2017;88(9):871-875. doi: 10.3357/AMHP.4919.2017. PMID: 28818147.	Wrong study design
Meier SM, Dalsgaard S, Mortensen PB, Leckman JF, Plessen KJ. Mortality risk in a nationwide cohort of individuals with tic disorders and with tourette syndrome. Mov Disord. 2017 Apr;32(4):605-609. doi: 10.1002/mds.26939. Epub 2017 Mar 24. PMID: 28339122.	Did not have ADHD/autism as an exposure
Ståhlberg O, Boman S, Robertsson C, Kerekes N, Anckarsäter H, Nilsson T. A 3-year follow-up study of Swedish youths committed to juvenile institutions: Frequent occurrence of criminality and health care use regardless of drug abuse. Int J Law Psychiatry. 2017 Jan-Feb;50:52-60. doi: 10.1016/j.ijlp.2016.09.004. Epub 2016 Oct 13. PMID: 27745884.	Wrong study design
Smith DaWalt L, Hong J, Greenberg JS, Mailick MR. Mortality in individuals with autism spectrum disorder: Predictors over a 20-year period. Autism. 2019;23(7):1732-1739. doi: 10.1177/1362361319827412. Epub 2019 Feb 28. PMID: 30818975.	Wrong study design
Anastopoulos AD, DuPaul GJ, Weyandt LL, Morrissey-Kane E, Sommer JL, Rhoads LH, Murphy KR, Gormley MJ, Gudmundsdottir BG. Rates and Patterns of Comorbidity Among First-Year College Students With ADHD. J Clin Child Adolesc Psychol. 2018;47(2):236-247. doi: 10.1080/15374416.2015.1105137. PMID: 26852645.	Did not have mortality as an outcome
Agnafors S, Torgerson J, Rusner M, Kjellström AN. Injuries in children and adolescents with psychiatric disorders. BMC Public Health. 2020;20(1):1273. doi: 10.1186/s12889-020-09283-3. PMID: 32838787.	Did not have mortality as an outcome
Chen VC, Chan HL, Wu SI, Lu ML, Dewey ME, Stewart R, Lee CT. Methylphenidate and mortality in children with attention-deficit hyperactivity disorder: population-based cohort study. Br J Psychiatry. 2020 Jul 14:1-9. doi: 10.1192/bjp.2020.129. PMID: 32662370.	Did not have ADHD/autism as an exposure
Houghton R, de Vries F, Loss G. Psychostimulants/Atomoxetine and Serious Cardiovascular Events in Children with ADHD or Autism Spectrum Disorder. CNS Drugs. 2020 Jan;34(1):93-101. doi: 10.1007/s40263-019-00686-4. PMID: 31768949.	Did not have ADHD/autism as an exposure
Peden AE, Willcox-Pidgeon S. Autism spectrum disorder and unintentional fatal drowning of children and adolescents in Australia: an epidemiological analysis. Arch Dis Child. 2020;105(9):869-874. doi: 10.1136/archdischild-2019-318658. PMID: 32169851.	Wrong study design
Gilmore D, Harris L, Longo A, Hand BN. Health status of Medicare-enrolled autistic older adults with and without co-occurring intellectual disability: An analysis of inpatient and institutional outpatient medical claims. Autism. 2021 Jan;25(1):266-274. doi: 10.1177/1362361320955109. PMID: 32907348.	Did not have mortality as an outcome
Leone M, Kuja-Halkola R, Leval A, D'Onofrio BM, Larsson H, Lichtenstein P, Bergen SE. Association of Youth Depression With Subsequent Somatic Diseases and Premature Death. JAMA Psychiatry. 2021 Mar 1;78(3):302-310. doi: 10.1001/jamapsychiatry.2020.3786. PMID: 33295952.	Did not have ADHD/autism as an exposure
Risnes K, Bilsteen JF, Brown P, Pulakka A, Andersen AN, Opdahl S, Kajantie E, Sandin S. Mortality Among Young Adults Born Preterm and Early Term in 4 Nordic Nations. JAMA Netw Open. 2021 Jan 4;4(1):e2032779. doi: 10.1001/jamanetworkopen.2020.32779. Erratum in: JAMA Netw Open. 2021 Feb 1;4(2):e210068. PMID: 33416885.	Did not have ADHD/autism as an exposure

© 2022 Catalá-López F et al. *JAMA Pediatrics*

eTable 7. List of studies with authors contacted for additional clarifications or data.

Study reference	Contacted author	Number of patients	Response from authors (Yes/No)	Additional data (Yes/No)
Pickett JA, Paculdo DR, Shavelle RM, Strauss DJ. 1998-2002 Update on "Causes of death in autism". J Autism Dev Disord. 2006;36(2):287-8.	Pickett, JA	13111 ASD patients	Yes	No
Mouridsen SE, Brønnum-Hansen H, Rich B, Isager T. Mortality and causes of death in autism spectrum disorders: an update. Autism. 2008;12(4):403-14.	Mouridsen, SE	341 ASD patients	No	No
Gillberg C, Billstedt E, Sundh V, Gillberg IC. Mortality in autism: a prospective longitudinal community-based study. J Autism Dev Disord. 2010;40(3):352-7.	Gillberg, C	120 ASD patients	Yes	No
Bilder D, Botts EL, Smith KR, Pimentel R, Farley M, Viskochil J, McMahon WM, Block H, Ritvo E, Ritvo RA, Coon H. Excess mortality and causes of death in autism spectrum disorders: a follow up of the 1980s Utah/UCLA autism epidemiologic study. J Autism Dev Disord. 2013;43(5):1196-204.	Bilder, D	305 ASD patients	Yes	No
Chang Z, Lichtenstein P, D'Onofrio BM, Sjölander A, Larsson H. Serious transport accidents in adults with attention-deficit/hyperactivity disorder and the effect of medication: a population-based study. JAMA Psychiatry. 2014;71(3):319-25.	Chang, Z	17408 ADHD patients	Yes	No
Dalsgaard S, Østergaard SD, Leckman JF, Mortensen PB, Pedersen MG. Mortality in children, adolescents, and adults with attention deficit hyperactivity disorder: a nationwide cohort study. Lancet. 2015 30;385(9983):2190-6.	Dalsgaard, S	32061 ADHD patients	Yes	Yes
Hirvikoski T, Mittendorfer-Rutz E, Boman M, Larsson H, Lichtenstein P, Bölte S. Premature mortality in autism spectrum disorder. Br J Psychiatry. 2016;208(3):232-8.	Hirvikoski, T	27122 ASD patients	Yes	No
London AS, Landes SD. Attention Deficit Hyperactivity Disorder and adult mortality. Prev Med. 2016;90:8-10.	London, AS	654 ADHD patients	Yes	No
Chen VC, Chan HL, Wu SI, Lee M, Lu ML, Liang HY, Dewey ME, Stewart R, Lee CT. Attention-Deficit/Hyperactivity Disorder and Mortality Risk in Taiwan. JAMA Netw Open. 2019;2(8):e198714.	Lee, CT	275980 ADHD patients	No	No
Hwang YIJ, Srasuebkul P, Foley KR, Arnold S, Trollor JN. Mortality and cause of death of Australians on the autism spectrum. Autism Res. 2019;12(5):806-815.	Hwang, YU	35929 ASD patients	No	No
Sun S, Kuja-Halkola R, Faraone SV, D'Onofrio BM, Dalsgaard S, Chang Z, Larsson H. Association of Psychiatric Comorbidity With the Risk of Premature Death Among Children and Adults With Attention-Deficit/Hyperactivity Disorder. JAMA Psychiatry. 2019 Aug 7. doi: 10.1001/jamapsychiatry.2019.1944.	Sun, S	86670 ADHD patients	Yes	Yes
Smith GS, Fleming M, Kinnear D, Henderson A, Pell JP, Melville C, Cooper SA. Mortality in 787,666 school pupils with and without autism: A cohort study. Autism. 2020 Aug 24:1362361320944037. doi: 10.1177/1362361320944037.	Smith, GS	9754 ASD patients	Yes	No

© 2022 Catalá-López F et al. *JAMA Pediatrics*

eTable 8. Main characteristics of included studies.

Author, year	Study design (country)	Setting; Coverage	Study years (Follow-up, y)	No. of Participants	Characteristics of Participants by Sex; Age; Race/Ethnicity; Social Status; Comorbidities	No. of Fatal cases	Main outcome(s)	Causes of death	Exposure and outcome definitions	Comparator	Endpoint Measure	Adjustment for confounding factors
Kuperman et al,[33] 1988	Retrospective cohort (United States)	Inpatient; population-based	1970-1985 (9.1)	110 people with ADHD	20.0% female; mean age, 12.3 y (at admission) and 22.1 y (at FU); 97% white; NA; CC (11.5% male; 4.8% female)	1	Specific cause of death	Yes	E: ICD-9 codes 314.x (attention deficit disorder)<br>O: ICD-9 codes E800-E999 (external)	General population	SMR	Age, sex and calendar year
Pickett et al,[13] 2006; Shavelle et al,[34] 2001; Shavelle et al,[35]	Retrospective cohort (United States)	Community; population-based	1983-1997; 1998-2002 (9.5)	13111 people with ASD	20.6% female; most aged ≥ 4 y (at first evaluation); 50% white, 16% Hispanic, 13% black, 6% Asian, 15% other; NA; 30% moderate to profound mental retardation, 4.2% seizures	280	All-cause and specific cause of death	Yes	E: ICD-9 codes (autism)<br>O: ICD-9 codes 001-E999 (any cause), 140-239 (neoplasms), 345, 436, 780.3 (seizures), 320-389, excl. 345 (nervous system), 390-459 (circulatory), 460-519 (respiratory), 520-579 (digestive), 740-759 (congenital anomalies), E800-E999 (external)	General population	SMR	Age and sex
Barkley et al,[36] 2008; Barkley et al,[37] 1990	Retrospective cohort (United States)	Outpatient; health services	1979-1996 (17 y)	158 people with ADHD and their parents	9% female; aged ≥ 4 y (at first evaluation) and 21 y (at FU); 94% white, 5% black, 1% Hispanic; NA; 43% CC	3	All-cause and specific cause of death	Yes	E: Interview with patient report, DSM-III-R (hyperactivity)<br>O: deaths (any cause, circulatory, external)	Participants without hyperactivity	RR	None
Mouridsen et al,[38] 2008; Isager et al,[39] 1999	Retrospective cohort (Denmark)	Inpatient; population-based	1960-2006 (35.5 y)	341 people with ASD	24.9% female; mean age, 7.5 y (at admission) and 43.4 (at FU); NA; NA; NA	26	All-cause and specific cause of death	Yes	E: ICD-9, ICD-10 codes (childhood autism, atypical autism, Asperger syndrome, other childhood disintegrative disorders)<br>O: deaths (any cause, infectious, nervous system, circulatory, external)	General population	SMR	Age, sex and calendar year
Gillberg et al,[40] 2010	Prospective cohort (Sweden)	Community; population-based	1962-2008 (33.2 y)	120 people with ASD	29.2% female; early childhood (at admission) and mean age 33.2 y (at FU); NA, NA; 52.5% any medical condition, 38.3% epilepsy	9	All-cause and specific cause of death	Yes	E: DSM-III, DSM-III-R codes (autism, atypical autism)<br>O: deaths (any cause, infectious, neoplasms, nervous system, circulatory, external)	General population	SMR	Age, sex and calendar year
Klein et al,[41] 2012	Prospective cohort (United States)	Community; school-based	1970-NA (33 y)	207 people with ADHD	All male; mean age, 8.3 y (at first evaluation) and 41 y (at FU); 100% white; NA; no CC	15	All-cause of death	No	E: DSM-IV codes (attention deficit hyperactivity disorder, combined type)<br>O: deaths (any cause)	Participants without ADHD	RR	None
Barbaresi et al,[42] 2013	Prospective cohort (United States)	Community; population-based	1976-2009 (NA)	367 people with ADHD	28.0% female; mean age, 10.4 y (at diagnosis) and 27.0 y (at FU); 99% white; maternal education, 18% college graduate; 9% not	7	All-cause and specific cause of death	Yes	E: DSM-IV codes (ADHD)<br>O: deaths (any cause, external)	Participants without ADHD	SMR	Age and sex

© 2022 Catalá-López F et al. *JAMA Pediatrics*

					married; 22% substance abuse, 70% any learning disability							
Bilder et al,[14] 2013	Retrospective cohort (United States)	Community; population-based	1982-2011 (25 y)	305 people with ASD and their siblings	25.2% female; mean age, 10.8 y (at diagnosis) and 35.8 (at FU); NA; NA; 42% severe intellectual disability	29	All-cause and specific cause of death	Yes	E: DSM-III-R, DSM-IV codes (ASD)<br>O: ICD-9, ICD-10 codes (infectious, neoplasms, endocrine/metabolic, mental, nervous system, circulatory, respiratory, congenital anomalies, external)	General population; Siblings	HR	Age, sex, and birth weight
Fairthorne et al,[43] 2014	Retrospective cohort (Australia)	Community; population-based	1983-2010 (NA)	2041 mothers of children with ASD	All women; most aged 20-34 y (77%), mean age of death, 42 y; NA; 23% high socioeconomic status; 69% children with intellectual disability	24	All-cause and specific cause of death	Yes	E: interview with mothers (ASD)<br>O: ICD-9, ICD-10 codes (any cause, neoplasms, circulatory, respiratory, digestive, pregnancy, congenital anomalies, external)	Participants without ASD or intellectual disability	HR	Age
Dalsgaard et al,[16] 2015; Scott et al,[44] 2017	Retrospective cohort (Denmark)	Community; population-based	1981-2013 (32 y)	32061 people with ADHD	26.4% female; mean age, 12.3 y (at diagnosis); NA; NA; 16.9% ODD/CD, 12.3% SUS	107	All-cause and specific cause of death	Yes	E: ICD-8, ICD-10 codes 308.01, F90x or F98.8 (hyperkinetic disorders)<br>O: ICD-8, ICD-10 codes (any cause, natural and unnatural/external)	General population	RR	Age, sex, calendar year, parental history of psychiatric disorders, parental educational and employment
Koisaari et al,[45] 2015	Prospective cohort (Finland)	Community; health services (maternity hospital)	1971-2004 (40 y)	122 people with ADHD	29.5% female; aged ≥ 5 y (at first evaluation), mean age 40 y (at FU); NA; NA; NA	11	All-cause and specific cause of death	Yes	E: DSM-II codes (hyperkinetic reaction of childhood, minimal brain dysfunction), DSM-IV codes (ADHD)<br>O: deaths (any cause, nervous system, circulatory, endocrine, digestive, external)	Participants without ADHD	RR	None
Hetchtman et al,[46] 2016	Prospective cohort (United States)	Community; school-based	1992-2008 (16 y)	476 people with ADHD	19.7% female; mean age 8.5 y (at diagnosis) and 24.7 y (at FU); 61% white, 20% African American, 8% Hispanic; NA; 40% ODD, 33% anxiety disorder, 14% CD, 11% tic disorder, 4% affective disorder, 2% mania/hypomania	11	All-cause and specific cause of death	Yes	E: DSM-IV codes (ADHD, combined type)<br>O: deaths (any cause, external)	Participants without ADHD	RR	Age and sex
Hirvikoski et al,[47] 2016	Retrospective case-cohort/case-control (Sweden)	Mixed; population-based	1987-2009 (NA)	27122 people with ASD	31.1% female; mean age 19.8 y (at diagnosis); NA; NA; NA	706	All-cause and specific cause of death	Yes	E: ICD-9 codes 299x (ASD), ICD-10 codes F84.0 (autism), F84.1 (atypical autism), F84.2 (other childhood disintegrative disorder), F84.5 (Asperger syndrome), F84.8 (other pervasive developmental disorders),	General population	OR	Age, sex and county of residence

© 2022 Catalá-López F et al. *JAMA Pediatrics*

									and F84.9 (pervasive developmental disorders not otherwise specified)<br>O: ICD-9, ICD-10 codes (any cause, infections, neoplasms, endocrine/metabolic, mental, nervous system, circulatory, respiratory, digestive, genitourinary, congenital anomalies, external)			
Hosking et al,[48] 2016	Retrospective cohort (United Kingdom)	Outpatient; population-based	2009-2013 (3 y)	1532 people with ASD	58.1% female; mean age, 39.9 y (at FU); NA; NA; all children with intellectual disability	15	All-cause of death	No	E: electronic health records in primary care – CPRD (intellectual disability patients with ASD)<br>O: ICD-10 codes (any cause)	General population	HR	Age, sex, UK general practice, comorbidities, deprivation, smoking status
London and Landes,[18] 2016	Retrospective cohort (United States)	Community; population-based	2007-2011 (4 y)	654 people with ADHD	54.4% female; mean age, 47.6 y (at FU); NA; 82% white, 11% Hispanic, 14% foreign-born; NA	13	All-cause and specific cause of death	Yes	E: validated diagnosis with patient report (ADHD)<br>O: deaths (any cause)	General population	OR	Age, sex, race/ethnicity, foreign-born
Schendel et al,[49] 2016	Prospective cohort (Denmark)	Community; population-based	1980-2013 (NA)	20492 people with ASD	22.4% female; most aged > 9 y (at diagnosis) median age, 19.0 y (at death); NA; NA; 68% mental/behavioral disorders (not ASD) and 12% neurological disorders: 27% ADHD, 16% intellectual disability, 8% episodic and paroxysmal disorders, 6% epilepsy	68	All-cause and specific cause of death	Yes	E: ICD-8 codes 299.00, 299.01, 299.02, and 299.03 (ASD), ICD-10 codes F84.0 (autism), F84.1 (atypical autism), F84.5 (Asperger syndrome), F84.8 (other pervasive developmental disorders), and F84.9 (pervasive developmental disorders not otherwise specified)<br>O: ICD-8, ICD-10 codes (any cause, natural causes: nervous system, unnatural/external: accidents, self-harm)	General population	HR	Age (birth age, gestational age, parental age), sex, and birth weight
Chen et al,[19] 2019	Prospective cohort (Taiwan)	Mixed; population-based	2000-2013 (14 y)	275980 people with ADHD	24.1% female; mean age, 9.6 y (at evaluation); 100% Asian; 22% level income ≥ NT$ 40,000; 98% anxiety disorder, 3% intellectual disability, 2% autism, 2% congenital anomaly	727	All-cause and specific cause of death	Yes	E: ICD-9 codes 314.x (attention deficit disorder)<br>O: ICD-9 codes (any cause, natural and unnatural/external)	General population	HR	Age, sex, income, comorbidities, outpatient visits
Hwang et al,[21] 2019	Retrospective cohort (Australia)	Community; population-based	2001-2015	35929 people with ASD	20.5% female; age ranged 5-64 y, mean age, 35.0 y (at death); NA; NA; 44.8% intellectual disability	244	All-cause and specific cause of death	Yes	E: ICD-10 codes F84.0 (autism), F84.1 (atypical autism), F84.5 (Asperger syndrome), F84.8 (other pervasive developmental disorders), and F84.9 (pervasive developmental disorders not otherwise specified)	General population	SMR	Age and sex

© 2022 Catalá-López F et al. *JAMA Pediatrics*

									E: ICD-10 codes (any cause, neoplasms, nervous system, circulatory, external)			
Kirby et al,[50] 2019	Retrospective cohort (United States)	Community; population-based	1998-2017 (20 y)	16904 people with ASD	24.0% female; mean age, 18.4 (at FU); NA; 72% white, 8% Hispanic; NA	49	Specific cause of death	Yes	E: ICD-9 codes 299.x, ICD-10 codes F84.x (ASD)<br>O: deaths (external: suicide death)	General population	RR	Sex
Sun et al,[17] 2019	Prospective cohort (Sweden)	Community; population-based	1983-2013 (11.1 y)	86670 people with ADHD	33.2% female; mean age, 14.3 y (at diagnosis); NA; parental education, 10% postgraduate, 34% postsecondary, 51% upper secondary; parental employment, 90% employed; 19% anxiety disorder, 19% depression, 18% ASD, 7% intellectual disability, 7% conduct disorder, 4% personality disorder	424	All-cause and specific cause of death	Yes	E: ICD-10 codes F90x or the first prescription of ADHD medication (attention deficit hyperactivity disorder)<br>O: ICD-10 codes (any causes, natural and unnatural/external)	General population	HR	Age (birth age, maternal age), sex, parental educational level, parental employment status, and birth weight
Yeh et al,[51] 2019	Retrospective case-control (United States)	Mixed; population-based	2000-2013 (NA)	4416 people with ADHD	22.5% female; most aged 40-64 y (at evaluation); NA; 10% low income, 62% < 25% college graduate	67	Specific cause of death	Yes	E: ICD-9 codes 314.x (attention deficit disorder)<br>O: ICD-10 codes X60-X84, Y87.0 (external: suicide)	General population	OR	Age, sex, insurance type, poverty level, and education
Fitzgerald et al,[52] 2019	Retrospective cohort (Denmark)	Community; population-based	1995-2014 (21.5 y)	32540 people with ADHD	30.8% female; mean age, 21.5 (at follow-up); NA; 0.7% anxiety, 0.4% affective disorders, 0.4% ODD/CD, 0.4% SUS substance use disorder, 0.1% ASD, and other (less than 0.1%)	35	Specific cause of death	Yes	E: ICD-8, ICD-10 codes 308.01, F90x or F98.8 (hyperkinetic disorders)<br>O: ICD-8, ICD-10 codes (external causes: suicide)	General population	IRR	Age, sex, calendar year
Akobirshoev et al,[53] 2020	Retrospective cohort (United States)	Inpatient; population-based	2004-2014 (NA)	34237 people with ASD	24.7% female; mean age, 33.1 y (at evaluation); 65% white, 11% black, 5% Hispanic; 26% private insurance, 54% psychiatric comorbidities, 21% epilepsy	462	All-cause of death	No	E: ICD-9 codes 299.0x, 299.8x, or 299.9x (ASD)<br>O: in-hospital deaths (any cause)	General population	OR	Age, sex, race/ethnicity, income, comorbidities, hospital
Jokiranta-Olkoniemi et al,[54] 2020	Retrospective cohort (Finland)	Mixed; population-based	1987-2015 (NA)	4695 people with ASD	20.4% female; mean age 8.0 y (at diagnosis); NA; NA; 78% comorbid psychiatric disorder: 24% affective and anxiety disorders, 13% intellectual disability, 3% SUD	53	All-cause and specific cause of death	Yes	E: ICD-10 codes F84.0 (autism), F84.5 (Asperger syndrome), F84.8 (other pervasive developmental disorders), and F84.9 (pervasive developmental disorders not otherwise specified)<br>O: ICD-8, ICD-9, ICD-10 codes (any cause, natural, accidents, suicides)	Participants without ASD	HR	Sex, maternal socioeconomic status, comorbid psychiatric disorders, death in the family

© 2022 Catalá-López F et al. *JAMA Pediatrics*

Huang et al,[55] 2021	Retrospective cohort (Taiwan)	Mixed; population-based	2000-2015 (8.1 y)	6599 people with ASD	22.8% female; mean age, 11.9 y (at evaluation); 100% Asian; 10% level income ≥ NT$ 35,000; 30% anxiety disorder, 23% ADHD, 22% intellectual disability, 17% tic disorder, 10% epilepsy	119	All-cause of death	No	E: ICD-9 codes 299 (autism spectrum disorder)<br>O: ICD-9 codes (any cause)	General population	HR	Age, sex, income, comorbidities, hospitalizations, education, behavioral psychotherapy, FU period
Kõlves et al,[56] 2021	Retrospective cohort (Denmark)	Community; population-based	1995-2016 (NA)	35020 people with ASD	26.6% female; most aged 10-29; NA; 72% any other psychiatric disorder; 33% ADHD, 16% affective disorders, 15% depression, 15% intellectual disability, and other	53	Specific cause of death	Yes	E: ICD-8 codes 299.00 to 299.01, ICD-10 codes F84.0 to F84.1 and F84.5 to F84.9 (ASD)<br>O: suicide deaths	General population	IRR	Age, sex, calendar year
Smith et al,[15] 2021	Retrospective cohort (Scotland, United Kingdom)	Community; school-based	2008-2015 (3.9 y)	9754 people with ASD	14.0% female; most aged 10-19; 92% white, 2% Asian, 1% mixed; 24.0% most deprived according to SIMD; 43% curriculum adaptation disability, 27% communication adaptation disability, 7% physical adaptation disability	6	All-cause of death	No	E: record linkage in educational data (autism)<br>O: ICD-10 codes (any causes)	Participants without ASD	SMR	Age and sex

CD: conduct disorder; CPRD: Clinical Practice Research Datalink; DSM: Diagnostic and Statistical Manual of Mental Disorders; SIMD: Scottish Index of Multiple Deprivation; E: exposure; ICD: International Classification of Disease; NA: not available; NT$: New Taiwan dollars (1 US$ = 32.1 NT$ in 2010); ODD: oppositional defiant disorder; O: outcome; HR = hazard ratio; IRR = incidence rate ratio; OR = odds ratio; RR = rate ratio; SMR = standardized mortality ratio; SUD: substance use

© 2022 Catalá-López F et al. *JAMA Pediatrics*

eTable 9. Main results of individual studies.

Author, year	Study design (country)<br>Setting, coverage	Number of participants (sex)	Endpoint measure	Adjustment for confounding factors	Point estimates (95% confidence intervals)
Kuperman et al,[33] 1988	Retrospective cohort (United States);<br>Inpatient, population-based	110 patients with ADHD (20% girls and women)	SMR	Age, sex and calendar year	Cause-specific mortality:<br>External causes (both): 0.79 (0.02-4.42)[a]
Pickett et al,[13] 2006; Shavelle et al,[34] 2001; Shavelle et al,[35]	Retrospective cohort (United States);<br>Community, population-based	13111 patients with ASD (21% girls and women)	SMR	Age and sex	All-cause mortality:<br>All-cause (both, 1983-2002): 2.45 (2.18-2.76)[a]<br>All-cause (men,1983-2002): 1.86 (1.63-2.14)[a]<br>All-cause (women,1983-2002): 5.35 (4.27-6.72)[a]<br>All-cause (both, 1998-2002): 2.60 (2.07-3.22)<br>All-cause (men,1998-2002): 2.30 (1.78-2.92)<br>All-cause (women,1998-2002): 5.33 (3.16-8.48)<br>All-cause (both,1983-1997): 2.40 (2.09-2.75)<br>All-cause (men,1983-1997): 1.70 (1.44-2.00)<br>All-cause (women,1983-1997): 5.36 (4.12-6.87)<br>Cause specific mortality (1983-1997):<br>Neoplasms (both): 2.58 (1.67-3.97)[a]<br>Nervous system and sense organs (both): 16.76 (11.14-25.21)[a]<br>Respiratory (both): 5.86 (3.62-9.48)[a]<br>Digestive (both): 6.74 (3.87-11.74)[a]<br>Congenital anomalies (both): 7.16 (4.35-11.78)[a]<br>External causes (both): 4.50 (3.56-5.69)[a]<br>Natural causes (both): 6.73 (5.46-8.30)[a]
Barkley et al,[36] 2008; Barkley et al,[37] 1990	Retrospective cohort (United States);<br>Outpatient, health services	158 patients with ADHD (9% girls and women), and their parents	RR	None	All-cause mortality:<br>All-cause (both): 0.77 (0.13-4.51)[a]<br>All-cause (fathers): 2.56 (0.12-52.75)[a]<br>All-cause (mothers): 1.54 (0.06-37.32)[a]<br>Cause specific mortality:<br>External causes (both): 1.54 (0.16-14.55)[a]<br>External causes (fathers): 2.56 (0.12-52.75)[a]<br>External causes (mothers): 1.54 (0.06-37.32)[a]
Mouridsen et al,[38] 2008; Isager et al,[39] 1999	Retrospective cohort (Denmark);<br>Inpatient, population-based	341 patients with ASD (25% girls and women)	SMR	Age, sex and calendar year	All-cause mortality:<br>All-cause (both): 1.93 (1.26-2.82)<br>All-cause (men): 1.57 (0.93-2.48)<br>All-cause (women): 4.01 (1.73-7.90)<br>Cause specific mortality:<br>Nervous system and sense organs (both) - epilepsy: 35.0 (9.5-89.6)
Gillberg et al,[40] 2010	Prospective cohort (Sweden);<br>Community, population-based	120 patients with ASD (29% girls and women)	SMR	Age, sex and calendar year	All-cause mortality:<br>All-cause (both): 5.56 (2.71-10.20)<br>All-cause (men): 2.26 (0.57-6.14)<br>All-cause (women): 20.69 (8.39-43.03)

© 2022 Catalá-López F et al. *JAMA Pediatrics*

Klein et al,[41] 2012	Prospective cohort (United States); Community, school-based	207 patients with ADHD (0% girls and women)	RR	None	All-cause mortality:<br>All-cause (men): 2.58 (0.96-6.96)[a]
Barbaresi et al,[42] 2013	Prospective cohort (United States); Community, population-based	367 patients with ADHD (28% girls and women)	SMR	Age and sex	All-cause mortality:<br>All-cause (both): 1.88 (0.83-4.26)<br>Cause specific mortality:<br>External causes (both): 2.78 (1.12-6.90)[a]
Bilder et al,[14] 2013	Retrospective cohort (United States); Community, population-based	305 patients with ASD (25% girls and women)	HR	Age (maternal, gestational, at mother's death, at father's death), sex, and birth weight	All-cause mortality:<br>All-cause (both): 11.59 (6.24-21.53)<br>All-cause (men): 9.91 (4.77-20.60)<br>All-cause (women): 30.14 (6.31-143.87)<br>All-cause (siblings): 1.19 (0.28-4.98)[a]
Fairthorne et al,[43] 2014	Retrospective cohort (Australia); Community, population-based	2041 mothers of children with ASD (100% girls and women)	HR	Age	All-cause mortality:<br>All-cause (mother): 1.60 (1.06-2.40)[a]<br>Cause specific mortality:<br>Neoplasms (mother): 1.54 (0.80-2.90)
Dalsgaard et al,[16] 2015; Scott et al,[44] 2017	Retrospective cohort (Denmark); Community, population-based	32061 patients with ADHD (26% girls and women)	RR	Age, sex, calendar year, parental history of psychiatric disorders, maternal/paternal age, parental educational and employment status	All-cause mortality:<br>All-cause (both): 2.07 (1.70-2.50)<br>All-cause (men): 1.93 (1.55-2.38)<br>All-cause (women): 3.01 (1.87-4.55)<br>Cause specific mortality:<br>Natural causes (both): 1.70 (1.11-2.47)<br>Natural causes (men): 1.38 (0.82-2.16)<br>Natural causes (women): 3.33 (1.51-6.24)<br>External causes (both): 2.40 (1.81-3.13)<br>External causes (men): 2.29 (1.69-3.04)<br>External causes (women): 3.51 (1.50-6.85)
Koisaari et al,[45] 2015	Prospective cohort (Finland); Community, health services	122 patients with ADHD (29% girls and women)	RR	None	All-cause mortality:<br>All-cause (both): 17.72 (1.06-296.8)[a]<br>All-cause (men): 9.77 (0.59-162.6)[a]<br>All-cause (women): 4.50 (0.19-107.4)[a]<br>Cause specific mortality:<br>Endocrine, nutritional and metabolic (both): 2.29 (0.09-55.64)[a]<br>Nervous system (both): 2.29 (0.09-55.64)[a]<br>Circulatory (both): 3.85 (0.19-79.28)[a]<br>Digestive (both): 2.29 (0.09-55.64)[a]<br>External causes (both): 10.02 (0.57-175.5)[a]<br>Natural causes (both): 2.64 (0.54-12.83)[a]
Hetchtman et al,[46] 2016	Prospective cohort (United States); Community, school-based	476 patients with ADHD (20% girls and women)	RR	Age and sex	All-cause mortality:<br>All-cause (both): 5.06 (0.65-39.32)[a]<br>Cause specific mortality:<br>External causes (both): 5.06 (0.65-39.32)[a]
Hirvikoski et al,[47] 2016	Retrospective case-cohort (Sweden); Mixed, population-based	27122 patients with ASD (31% girls and women)	OR	Age, sex and county of residence	All-cause mortality:<br>All-cause (both): 2.56 (2.38-2.76)

© 2022 Catalá-López F et al. *JAMA Pediatrics*

					All-cause (men): 2.87 (2.60-3.16)<br>All-cause (women): 2.24 (1.99-2.51)<br>Cause specific mortality:<br>Infectious (both): 1.83 (0.75-4.30)<br>Neoplasms (both): 1.80 (1.46-2.23)<br>Neoplasms (men): 1.79 (1.34-2.38)<br>Neoplasms (women): 1.83 (1.33-2.50)<br>Endocrine, nutritional and metabolic (both): 3.70 (2.34-5.87)<br>Endocrine, nutritional and metabolic (men): 2.11 (0.94-4.73)<br>Endocrine, nutritional and metabolic (women): 5.70 (3.25-9.99)<br>Mental and behavioral (both): 2.80 (1.94-4.03)<br>Mental and behavioral (men): 3.31 (1.85-5.92)<br>Mental and behavioral (women): 2.53 (1.58-4.05)<br>Nervous system (both): 7.49 (5.78-9.72)<br>Nervous system (men): 10.19 (7.27-14.29)<br>Nervous system (women): 5.29 (3.50-7.99)<br>Circulatory (both): 1.49 (1.27-1.75)<br>Circulatory (men): 2.02 (1.64-2.49)<br>Circulatory (women): 1.10 (0.86-1.40)<br>Respiratory (both): 2.68 (1.99-3.62)<br>Respiratory (men): 2.16 (1.36-3.42)<br>Respiratory (women): 3.24 (2.18-4.79)<br>Digestive (both): 3.31 (2.25-4.87)<br>Digestive (men): 3.81 (2.31-6.29)<br>Digestive (women): 2.78 (1.52-5.07)<br>Genitourinary (both): 3.82 (2.13-6.84)<br>Congenital anomalies (both): 19.10 (11.94-30.55)<br>Congenital anomalies (men): 11.07 (5.30-23.13)<br>Congenital anomalies (women): 33.86 (18.04-63.56)<br>External causes (both): 4.99 (4.13-6.04)[a]<br>External causes (men): 4.27 (3.39-5.39)[a]<br>External causes (women): 7.65 (5.48-10.68)[a]<br>Natural causes (both): 2.59 (2.36-2.85)[a]<br>Natural causes (men): 2.85 (2.50-3.24)[a]<br>Natural causes (women): 2.44 (2.12-2.80)[a]
Hosking et al,[48] 2016	Retrospective cohort (United Kingdom); Outpatient, population-based	1532 patients with ASD (58% girls and women)	HR	Age, sex, UK general practice, comorbidities, deprivation, smoking status	All-cause mortality:<br>All-cause (both): 2.22 (1.01-4.86)
London and Landes,[18] 2016	Prospective cohort (United States); Community, population-based	654 patients with ADHD (54% girls and women)	OR	Age, sex, race/ethnicity, foreign-born	All-cause mortality:<br>All-cause (both): 1.78 (1.01-3.12)

© 2022 Catalá-López F et al. *JAMA Pediatrics*

Schendel et al,[49] 2016	Prospective cohort (Denmark); Community, population-based	20492 patients with ASD (22.4% girls and women)	HR	Age (birth age, gestational age, parental age), sex, and birth weight	All-cause mortality:<br>All-cause (both): 2.00 (1.50-2.80)<br>All-cause (men): 1.80 (1.20-2.60)<br>All-cause (women): 3.50 (1.70-7.00)<br>Cause specific mortality:<br>Natural causes (both): 2.20 (1.50-3.20)<br>Nervous system (both):4.10 (2.00-8.80)<br>External causes (both): 2.00 (1.40-2.80)
Chen et al,[19] 2019	Prospective cohort (Taiwan); Mixed, population-based	275980 patients with ADHD (24% girls and women)	HR	Age, sex, income, comorbidities, outpatient visits	All-cause mortality:<br>All-cause (both): 1.07 (1.00-1.17)<br>All-cause (men): 1.04 (0.94-1.14)<br>All-cause (women): 1.24 (1.04-1.48)<br>Cause specific mortality:<br>Natural causes (both): 0.91 (0.80-1.15)<br>Natural causes (men): 0.83 (0.72-1.15)<br>Natural causes (women): 1.03 (0.82-1.30)<br>External causes (both): 1.51 (1.32-1.72)[a]<br>External causes (men): 1.45 (1.25-1.67)[a]<br>External causes (women): 1.82 (1.32-2.51)[a]
Hwang et al,[21] 2019	Retrospective cohort (Australia); Community, population-based	35929 patients with ASD (20% girls and women)	SMR	Age and sex	All-cause mortality:<br>All-cause (both): 2.06 (1.64-2.58)
Kirby et al,[50] 2019	Retrospective cohort (United States); Community, population-based	16094 patients with ASD (24% girls and women)	RR	Sex	Cause specific mortality:<br>External causes (both): 1.18 (0.89-1.56)[a]<br>External causes (men): 0.83 (0.61-1.12)[a]<br>External causes (women): 3.42 (1.63-7.20)
Sun et al,[17] 2019	Prospective cohort (Sweden); Community, population-based	86670 patients with ADHD (33% girls and women)	HR	Age (birth age, maternal age), sex, parental educational level, parental employment status, and birth weight	All-cause mortality:<br>All-cause (both): 3.94 (3.51-4.43)<br>All-cause (men): 4.63 (4.09-5.25)[a,b]<br>All-cause (women): 5.49 (4.46-6.76)[a,b]<br>Cause specific mortality:<br>Natural causes (both): 2.47 (1.66-3.68)<br>Natural causes (men): 1.76 (1.33-3.32)[b]<br>Natural causes (women): 2.70 (1.81-4.04)[b]<br>External causes (both): 6.48 (5.12-8.21)<br>External causes (men): 5.01 (4.40-5.71)[b]<br>External causes (women): 7.11 (5.58-9.06)[b]
Yeh et al,[51] 2019	Retrospective case-control (United States); Mixed, population-based	4416 patients with ADHD (22% girls and women)	OR	Age, sex, insurance type, poverty level, and education	Cause specific mortality:<br>External causes (both): 2.37 (1.79-3.15)<br>External causes (men): 1.92 (1.38-2.67)<br>External causes (women): 3.14 (1.80-5.50)
Fitzgerald et al,[52] 2019	Retrospective cohort (Denmark); Community, population-based	32540 patients with ADHD (31% girls and women)	IRR	Age, sex and calendar year	Cause specific mortality:<br>External causes (both): 3.20 (2.29-4.47)<br>External causes (men): 2.98 (2.06-4.30)

© 2022 Catalá-López F et al. *JAMA Pediatrics*

					External causes (women): 1.47 (0.66-3.27)
Akobirshoev et al,[53] 2020	Retrospective cohort (United States); Inpatient, population-based	34237 patients with ASD (25% girls and women)	OR	Age, sex, race/ethnicity, income, comorbidities, hospital (bed size and region)	All-cause mortality:<br>All-cause (both): 1.51 (1.33-1.72)<br>All-cause (men): 1.25 (1.08-1.45)<br>All-cause (women): 2.75 (2.09-3.64)
Jokiranta-Olkoniemi et al,[54] 2020	Retrospective cohort (Sweden); Mixed; population-based	4695 patients with ASD (20% girls and women)	HR	Sex, maternal socioeconomic status, comorbid psychiatric disorders, psychiatric disorders among family members, death in the family	All-cause mortality:<br>All-cause (both): 1.70 (1.20-2.60)<br>All-cause (men): 1.50 (0.9-2.30)<br>All-cause (women): 5.30 (1.70-16.30)<br>Cause specific mortality):<br>Natural (both): 6.10 (2.70-13.50)<br>Natural (men): 4.40 (2.00-9.80)<br>Natural (women): 29.60 (3.20-275.10)<br>External causes (both): 1.08 (0.65-1.78)[a]<br>External causes (men): 1.09 (0.65-1.83)[a]<br>External causes (women): 1.04 (0.17-6.27)[a]
Huang et al,[55] 2021	Retrospective cohort (Taiwan); Mixed; population-based	6599 patients with ASD (23% girls and women)	HR	Age, sex, income, comorbidities, hospitalizations, education, behavioral psychotherapy, follow-up period	All-cause mortality:<br>All-cause (both): 2.79 (2.44-3.18)[a]
Kõlves et al,[56] 2021	Retrospective cohort (Denmark); Community, population-based	35020 patients with ASD (27% girls and women)	IRR	Age, sex and calendar year	Cause specific mortality:<br>External causes (both): 3.75 (2.85-4.92)<br>External causes (men): 3.48 (2.57-4.74)<br>External causes (women): 2.63 (1.46-4.76)
Smith et al,[15] 2021	Retrospective cohort (Scotland); Community, school-based	9754 patients with ASD (14% girls and women)	SMR	Age and sex	All-cause mortality:<br>All-cause (both): 1.10 (0.50-2.50)

[a] Results were calculated based on the original data using a fixed-effect model (as defined in our study protocol). [b]Additional data provided by study authors. HR = hazard ratio; IRR = incidence rate ratio; OR = odds ratio; RR = rate ratio; SMR = standardized mortality ratio.

© 2022 Catalá-López F et al. *JAMA Pediatrics*

eTable 10. Methodological quality assessment of included studies (Newcastle-Ottawa Scale)

NEWCASTLE - OTTAWA QUALITY ASSESSMENT SCALE CASE CONTROL STUDIES

Note: A study can be awarded a maximum of one star for each numbered item within the Selection and Exposure categories. A maximum of two stars can be given for Comparability.

Selection

1) Is the case definition adequate?
   - a) yes, with independent validation *
   - b) yes, e.g. record linkage or based on self-reports
   - c) no description

2) Representativeness of the cases
   - a) consecutive or obviously representative series of cases *
   - b) potential for selection biases or not stated

3) Selection of Controls
   - a) community controls *
   - b) hospital controls
   - c) no description

4) Definition of Controls
   - a) no history of disease (endpoint) *
   - b) no description of source

Comparability

1) Comparability of cases and controls on the basis of the design or analysis
   - a) study controls for age and sex *
   - b) study controls for any additional factor (e.g. medication, smoking, comorbidities) *

Exposure

1) Ascertainment of exposure
   - a) secure record (eg surgical records) *
   - b) structured interview where blind to case/control status *
   - c) interview not blinded to case/control status
   - d) written self-report or medical record only
   - e) no description

2) Same method of ascertainment for cases and controls
   - a) yes *
   - b) no

3) Non-Response rate
   - a) same rate for both groups *
   - b) non-respondents described
   - c) rate different and no designation

NEWCASTLE - OTTAWA QUALITY ASSESSMENT SCALE COHORT STUDIES

Note: A study can be awarded a maximum of one star for each numbered item within the Selection and Outcome categories. A maximum of two stars can be given for Comparability

Selection

1) Representativeness of the exposed cohort
   - a) truly representative of the average in the community *
   - b) somewhat representative of the average in the community *
   - c) selected group of users (e.g. nurses, volunteers)
   - d) no description of the derivation of the cohort

2) Selection of the non-exposed cohort
   - a) drawn from the same community as the exposed cohort *
   - b) drawn from a different source
   - c) no description of the derivation of the non-exposed cohort

3) Ascertainment of exposure
   - a) secure record (e.g. surgical records) *
   - b) structured interview *
   - c) written self-report
   - d) no description

4) Demonstration that outcome of interest was not present at start of study
   - a) yes *
   - b) no

Comparability

1) Comparability of cohorts on the basis of the design or analysis
   - a) study controls for age and sex *
   - b) study controls for any additional factor (e.g. medication, smoking, comorbidities…) *

Outcome

1) Assessment of outcome
   - a) independent blind assessment *
   - b) record linkage *
   - c) self-report
   - d) no description

2) Was follow-up long enough for outcomes to occur
   - a) yes *
   - b) no

3) Adequacy of follow up of cohorts
   - a) complete follow up - all subjects accounted for *
   - b) subjects lost to follow up unlikely to introduce bias *
   - c) follow up rate < 40% (select an adequate %) and/or no description of those lost
   - d) no statement

© 2022 Catalá-López F et al. *JAMA Pediatrics*

eTable 10 (cont). Methodological quality assessment of included studies (Newcastle-Ottawa Scale)

Author, year	1. Selection				2. Comparability	3. Outcome			Total score	Quality assessment – risk of bias rating
	**1.1**	**1.2**	**1.3**	**1.4**	**2.1**	**3.1**	**3.2**	**3.3**		
Kuperman et al,[33] 1988	*	*	*	*	*	*	*		7	Low risk
Pickett et al,[13] 2006; Shavelle et al,[34] 2001; Shavelle et al,[35]	*	*	*	*	*	*	*		7	Low risk
Barkley et al,[36] 2008; Barkley et al,[37] 1990	*		*	*			*		4	Moderate risk
Mouridsen et al,[38] 2008; Isager et al,[39] 1999	*	*	*	*	*	*	*		7	Low risk
Gillberg et al,[40] 2010	*	*	*	*	*	*	*		7	Low risk
Klein et al,[41] 2012		*	*	*			*		4	Moderate risk
Barbaresi et al,[42] 2013	*	*	*	*	*	*	*	*	8	Low risk
Bilder et al,[14] 2013	*	*	*	*	**	*	*		8	Low risk
Fairthorne et al,[43] 2014	*	*			*	*			4	Moderate risk
Dalsgaard et al,[16] 2015; Scott et al,[44] 2017	*	*	*	*	**	*	*	*	9	Low risk
Koisaari et al,[45] 2015	*	*	*	*			*		5	Moderate risk
Hetchtman et al,[46] 2016	*	*	*	*	*		*		6	Moderate risk
Hirvikoski et al,[47] 2016	*	*	*		*	*	*	*	7	Low risk
Hosking et al,[48] 2016		*	*	*	**	*	*		7	Low risk
London and Landes,[18] 2016		*		*	**	*	*		6	Moderate risk
Schendel et al,[49] 2016	*	*	*	*	**	*	*	*	9	Low risk
Chen et al,[19] 2019	*	*	*	*	**	*	*	*	9	Low risk
Hwang et al,[21] 2019	*	*	*	*	*	*	*		7	Low risk
Kirby et al,[50] 2019	*	*	*	*		*	*		6	Moderate risk
Sun et al,[17] 2019	*	*	*	*	**	*	*	*	9	Low risk
Yeh et al,[51] 2019		*			**	*	*		5	Moderate risk
Fitzgerald et al,[52] 2019	*	*	*	*	*	*	*	*	8	Low risk
Akobirshoev et al,[53] 2020			*	*	**	*			5	Moderate risk
Jokiranta-Olkoniemi et al,[54] 2020	*	*	*	*	*		*		6	Moderate risk
Huang et al,[55] 2021	*	*	*	*	**	*	*	*	9	Low risk
Kõlves et al,[56] 2021	*	*	*	*	*	*	*	*	8	Low risk
Smith et al,[15] 2021	*	*	*	*	*	*			6	Moderate risk

Note: Risk of bias rating: 0-3 high risk of bias (low quality), 4-6 moderate risk of bias (moderate quality), 7-9 low risk of bias (high quality).

© 2022 Catalá-López F et al. *JAMA Pediatrics*

© 2022 Catalá-López F et al. *JAMA Pediatrics*

eTable 11. Grading certainty (or credibility) of evidence for summary estimates

<table>
<tr><td>Global Burden of Disease (GBD) criteria and GRADE system emphasize certainty (or credibility) of evidence based on randomized controlled data. For many risks in the biomedical literature (such as disease comorbidity), we will never have randomized controlled trials, and to restrict the assessment of exposures (or risks factors) to only those with trial evidence would lead us to ignore some of the most important determinants of health. Randomized controlled trials are unavailable for our research question. It is for this reason that we have used modified criteria of "convincing/high certainty" or "probable/moderate certainty" evidence for causality. If well designed and reported observational cohort studies form the evidence base the certainty rating starts with high/convincing evidence. If well designed and reported case-control studies form the evidence base the rating starts with moderate/probable evidence. Because the GRADE system does not yet have a scale for assessing non-interventional observational studies, we used a modified version to describe the validity and trustability of the evidence we presented in each meta-analysis. In brief, we rated the evidence as "convincing or high" when we are highly confident that the true effect lies close to that estimated. For example, evidence is judged as "convincing or high" if all of the following apply:
• There are multiple cohort studies included in the analyses with no major limitations (low risk of bias according to NOS scale)
• Summary effect estimate P value < 10^{-6}
• The summary estimate has a narrow confidence interval (95% predictive interval excluding null value)</td></tr>
<tr><td>We rated the evidence as "probable or moderate" when we consider the true effect is likely to be close to the estimate of the effect, but there is a possibility that it is substantially different. For example, evidence might be judged as "probable or moderate" if any of the following applies:
• There are only few studies and some have limitations but not major flaws (low/moderate risk of bias according to NOS scale)
• Summary effect estimate P value < 10^{-3}
• The 95% predictive interval is wide</td></tr>
<tr><td>Finally, we rated the evidence to be "low (limited, not conclusive)" when the true effect may be substantillay different from the estimate of its effect. "Low/limited-suggestive evidence" represents too limited evidence to conclude on a probable or convincing causal association, but where there is evidence suggestive of a direction of effect. "Low/limited-not conclusive evidence" consists of information that is so limited that no firm conclusion can be made for several reasons (e.g., the evidence might be limited by the amount of evidence in terms of the number of studies available, by inconsistency of direction of effect, by poor quality of studies, or by any combination of these factors). For example, evidence might be judged as "low quality" if any of the following apply:
• There is only one study or studies have major methological flaws (high risk of bias according to NOS scale)
• Summary effect estimate P value < 0.05
• There is considerable variation between study results
• The confidence interval of the summary estimate of the effect is very wide (95% predictive interval including null value or 95% predictive interval was inestimable e.g. less than 3 studies)
• Small study effects (sometimes called "publication bias")</td></tr>
</table>

© 2022 Catalá-López F et al. *JAMA Pediatrics*

Certainty of the evidence and reasons for each outcome of interest.

Outcomes of interest	Certainty of the evidence and reason
**Primary outcome of all cancer**	
All-cause mortality, ASD both	◯◯◯<br>Moderate confidence (probable)<br>Downgraded: (-1) heterogeneity
All-cause mortality, ASD male	◯◯<br>Low confidence (limited-suggestive)<br>Downgraded: (-1) heterogeneity; (-1) 95% prediction interval wide
All-cause mortality, ASD female	◯ ◯<br>Low confidence (limited-suggestive)<br>Downgraded: (-1) heterogeneity; (-1) 95% prediction interval wide
All-cause mortality, ADHD both	◯◯<br>Low confidence (limited-suggestive)<br>Downgraded: (-1) heterogeneity; (-1) 95% prediction interval wide
All-cause mortality, ADHD male	◯◯<br>Low confidence (limited-suggestive)<br>Downgraded: (-1) heterogeneity; (-1) 95% prediction interval wide
All-cause mortality, ADHD female	◯◯<br>Low confidence (limited-suggestive)<br>Downgraded: (-1) heterogeneity; (-1) 95% prediction interval wide
All-cause mortality, ASD first-degree relative (mother)	◯<br>Very low confidence (very uncertain)<br>Downgraded: (-1) only one study, (-2) 95% prediction interval inestimable
All-cause mortality, ASD first-degree relative (siblings)	◯<br>Very low confidence (very uncertain)<br>Downgraded: (-1) only one study, (-2) 95% prediction interval inestimable
All-cause mortality, ADHD first-degree relative (mother)	◯<br>Very low confidence (very uncertain)<br>Downgraded: (-1) only one study, (-2) 95% prediction interval inestimable
All-cause mortality, ADHD first-degree relative (father)	◯<br>Very low confidence (very uncertain)<br>Downgraded: (-1) only one study, (-2) 95% prediction interval inestimable
**Secondary outcome of site-specific cancer**	
Natural causes, ASD both	◯ ◯<br>Low confidence (limited-suggestive)<br>Downgraded: (-1) heterogeneity; (-1) 95% prediction interval wide
Natural causes, ASD male	◯◯<br>Low confidence (limited-suggestive)<br>Downgraded: (-2) 95% prediction interval inestimable
Natural causes, ASD female	◯◯<br>Low confidence (not conclusive)<br>Downgraded: (-1) heterogeneity; (-2) 95% prediction interval inestimable
Natural causes, ADHD both	◯ ◯<br>Low confidence (limited-suggestive)<br>Downgraded: (-1) heterogeneity; (-1) 95% prediction interval wide
Natural causes, ADHD male	◯◯<br>Low confidence (limited-suggestive)<br>Downgraded: (-1) heterogeneity; (-1) 95% prediction interval wide
Natural causes, ADHD female	◯◯<br>Low confidence (limited-suggestive)<br>Downgraded: (-1) heterogeneity; (-1) 95% prediction interval wide
Unnatural causes, ASD both	◯ ◯<br>Low confidence (limited-suggestive)<br>Downgraded: (-1) heterogeneity; (-1) 95% prediction interval wide
Unnatural causes, ASD male	◯ ◯<br>Low confidence (limited-suggestive)<br>Downgraded: (-1) heterogeneity; (-1) 95% prediction interval wide
Unnatural causes, ASD female	◯ ◯<br>Low confidence (limited-suggestive)<br>Downgraded: (-1) heterogeneity; (-1) 95% prediction interval wide
Unnatural causes, ADHD both	◯ ◯<br>Low confidence (limited-suggestive)<br>Downgraded: (-1) heterogeneity; (-1) 95% prediction interval wide
Unnatural causes, ADHD male	◯ ◯<br>Low confidence (limited-suggestive)<br>Downgraded: (-1) heterogeneity; (-1) 95% prediction interval wide
Unnatural causes, ADHD female	◯ ◯<br>Low confidence (limited-suggestive)

© 2022 Catalá-López F et al. *JAMA Pediatrics*

Outcomes of interest	Certainty of the evidence and reason
	Downgraded: (-1) heterogeneity; (-1) 95% prediction interval wide
Infections, ASD both	◯<br>Very low confidence (very uncertain)<br>Downgraded: (-1) only one study, (-2) 95% prediction interval inestimable
Neoplasms, ASD both	◯◯<br>Low confidence (limited-suggestive)<br>Downgraded: (-1) heterogeneity; (-1) 95% prediction interval very wide
Neoplasms, ASD male	◯<br>Very low confidence (very uncertain)<br>Downgraded: (-1) only one study, (-2) 95% prediction interval inestimable
Neoplasms, ASD female	◯<br>Very low confidence (very uncertain)<br>Downgraded: (-1) only one study, (-2) 95% prediction interval inestimable
Neoplasms, ASD first-degree relative (mother)	◯<br>Very low confidence (very uncertain)<br>Downgraded: (-1) only one study, (-2) 95% prediction interval inestimable
Endocrine, ASD both	◯<br>Very low confidence (very uncertain)<br>Downgraded: (-1) only one study, (-2) 95% prediction interval inestimable
Endocrine, ASD male	◯<br>Very low confidence (very uncertain)<br>Downgraded: (-1) only one study, (-2) 95% prediction interval inestimable
Endocrine, ASD female	◯<br>Very low confidence (very uncertain)<br>Downgraded: (-1) only one study, (-2) 95% prediction interval inestimable
Mental and behavioural disorders, ASD both	◯<br>Very low confidence (very uncertain)<br>Downgraded: (-1) only one study, (-2) 95% prediction interval inestimable
Mental and behavioural disorders, ASD male	◯<br>Very low confidence (very uncertain)<br>Downgraded: (-1) only one study, (-2) 95% prediction interval inestimable
Mental and behavioural disorders, ASD female	◯<br>Very low confidence (very uncertain)<br>Downgraded: (-1) only one study, (-2) 95% prediction interval inestimable
Nervous system, ASD both	◯◯<br>Low confidence (limited-suggestive)<br>Downgraded: (-1) heterogeneity; (-1) 95% prediction interval wide
Nervous system, ASD male	◯<br>Very low confidence (very uncertain)<br>Downgraded: (-1) only one study, (-2) 95% prediction interval inestimable
Nervous system, ASD female	◯<br>Very low confidence (very uncertain)<br>Downgraded: (-1) only one study, (-2) 95% prediction interval inestimable
Circulatory system, ASD both	◯<br>Very low confidence (very uncertain)<br>Downgraded: (-1) only one study, (-2) 95% prediction interval inestimable
Circulatory system, ASD male	◯<br>Very low confidence (very uncertain)<br>Downgraded: (-1) only one study, (-2) 95% prediction interval inestimable
Circulatory system, ASD female	◯<br>Very low confidence (very uncertain)<br>Downgraded: (-1) only one study, (-2) 95% prediction interval inestimable
Respiratory system, ASD both	◯◯<br>Low confidence (limited-suggestive)<br>Downgraded: (-1) heterogeneity; (-1) 95% prediction interval wide
Respiratory system, ASD male	◯<br>Very low confidence (very uncertain)<br>Downgraded: (-1) only one study, (-2) 95% prediction interval inestimable
Respiratory system, ASD female	◯<br>Very low confidence (very uncertain)<br>Downgraded: (-1) only one study, (-2) 95% prediction interval inestimable
Digestive system, ASD both	◯◯<br>Low confidence (limited-suggestive)<br>Downgraded: (-1) heterogeneity; (-1) 95% prediction interval very wide
Digestive system, ASD male	◯<br>Very low confidence (very uncertain)<br>Downgraded: (-1) only one study, (-2) 95% prediction interval inestimable
Digestive system, ASD female	◯<br>Very low confidence (very uncertain)

© 2022 Catalá-López F et al. *JAMA Pediatrics*

Outcomes of interest	Certainty of the evidence and reason
	Downgraded: (-1) only one study, (-2) 95% prediction interval inestimable
Genitourinary system, ASD both	◯<br>Very low confidence (very uncertain)<br>Downgraded: (-1) only one study, (-2) 95% prediction interval inestimable
Congenital malformations, ASD both	◯◯<br>Low confidence (limited-suggestive)<br>Downgraded: (-1) heterogeneity; (-1) 95% prediction interval wide
Congenital malformations, ASD male	◯<br>Very low confidence (very uncertain)<br>Downgraded: (-1) only one study, (-2) 95% prediction interval inestimable
Congenital malformations, ASD female	◯<br>Very low confidence (very uncertain)<br>Downgraded: (-1) only one study, (-2) 95% prediction interval inestimable
External causes, ASD first-degree relative (mother)	◯<br>Very low confidence (limited-not conclusive)<br>Downgraded: (-1) only one study, (-2) 95% prediction interval inestimable
External causes, ASD first-degree relative (father)	◯<br>Very low confidence (limited-not conclusive)<br>Downgraded: (-1) only one study, (-2) 95% prediction interval inestimable

© 2022 Catalá-López F et al. *JAMA Pediatrics*

eTable 12. Summary statistics of risk of mortality in people with ASD/ADHD people.

Outcomes of interest	No of studies	Participants with ASD or ADHD, No.	Death cases, No.	Pooled RR (95% CI)	RR largest study (95% CI)	P value for Effect Estimate	I^2 (95% CI)	95% prediction interval	P value for heterogeneity	Confidence
**Primary outcome, all-cause mortality**										
All-cause mortality, ASD both	12	154238	2017	2.37 (1.97-2.85)	2.06 (1.64-2.58)	<.001	89 (82-93)	1.25-4.48	< .001	Moderate (probable)
All-cause mortality, ASD male	8	75085	1071	2.09 (1.50-2.92)	1.25 (1.08-1.45)	<.001	94 (90-96)	0.68-6.40	< .001	Low (limited-suggestive)
All-cause mortality, ASD female	8	25338	562	4.87 (3.07-7.73)	2.75 (2.09-3.64)	<.001	91 (85-95)	1.09-21.78	< .001	Low (limited-suggestive)
All-cause mortality, ADHD both	8	396488	1302	2.13 (1.13-4.02)	1.07 (1.00-1.17)	.020	98 (97-99)	0.28-16.42	< .001	Low (limited-suggestive)
All-cause mortality, ADHD male	5	291201	982	2.43 (1.01-5.83)	1.04 (0.94-1.14)	.046	99 (98-99)	0.10-58.40	< .001	Low (limited-suggestive)
All-cause mortality, ADHD female	4	103839	302	2.84 (1.02-7.89)	1.24 (1.04-1.48)	.045	97 (96-99)	0.03-277.30	< .001	Low (limited-suggestive)
All-cause mortality, ASD first-degree relative (mother)	1	2041	24	NA	1.60 (1.06-2.40)	.024	NA	NA	NA	Very low (very uncertain)
All-cause mortality, ASD first-degree relative (siblings)	1	173	2	NA	1.19 (0.28-4.98)	.813	NA	NA	NA	Very low (very uncertain)
All-cause mortality, ADHD first-degree relative (mother)	1	158	2	NA	2.56 (0.12-52.75)	.545	NA	NA	NA	Very low (very uncertain)
All-cause mortality, ADHD first-degree relative (father)	1	158	1	NA	1.54 (0.06-37.32)	.792	NA	NA	NA	Very low (very uncertain)
**Secondary outcomes, cause-specific mortality**										
Natural causes, ASD both	4	65421	613	3.80 (2.06-7.01)	2.59 (2.36-2.85)	<.001	96 (92-98)	0.22-66.96	< .001	Low (limited-suggestive)
Natural causes, ASD male	2	22430	256	2.95 (2.35-3.69)	2.85 (2.50-3.24)	<.001	10 (NA)	NA	.290	Low (limited-suggestive)
Natural causes, ASD female	2	9387	220	6.57 (0.60-71.91)	2.44 (2.12-2.80)	.123	79 (NA)	NA	.028	Low (limited-not conclusive)
Natural causes, ADHD both	4	394833	516	1.62 (0.89-2.96)	0.91 (0.80-1.15)	.117	88 (72-95)	0.11-23.10	< .001	Low (limited-not conclusive)
Natural causes, ADHD male	3	290908	348	1.22 (0.74-2.01)	0.83 (0.72-1.15)	.428	80 (36-94)	0.00-451.06	.007	Low (limited-not conclusive)
Natural causes, ADHD female	3	103803	138	2.02 (0.91-4.48)	1.03 (0.82-1.30)	.085	91 (77-97)	$0.00\text{-}39.6\times10^{3}$	< .001	Low (limited-not conclusive)
Unnatural causes, ASD both	6	117345	318	2.50 (1.49-4.18)	3.75 (2.85-4.92)	.001	95 (92-97)	0.38-16.35	< .001	Low (limited-suggestive)
Unnatural causes, ASD male	4	61031	182	1.94 (0.83-4.49)	3.48 (2.57-4.74)	.124	96 (94-98)	0.03-112.04	< .001	Low (limited-not conclusive)
Unnatural causes, ASD female	4	22712	58	3.61 (1.71-7.60)	2.63 (1.46-4.76)	.001	79 (45-92)	0.15-88.22	.002	Low (limited-suggestive)
Unnatural causes, ADHD both	10	432900	847	2.81 (1.73-4.55)	1.51 (1.51-1.72)	<.001	92 (88-95)	0.61-12.91	< .001	Low (limited-suggestive)
Unnatural causes, ADHD male	5	316851	NA	2.49 (1.36-4.56)	1.45 (1.25-1.67)	.003	97 (96-98)	0.23-26.59	< .001	Low (limited-suggestive)
Unnatural causes, ADHD female	5	114816	NA	2.98 (1.46-6.10)	1.82 (1.32-2.51)	.003	92 (85-96)	0.20-44.17	< .001	Low (limited-suggestive)
Infections, ASD both	1	27122	5	NA	1.83 (0.75-4.30)	.175	NA	NA	NA	Very low (very uncertain)
Neoplasms, ASD both	2	40233	109	2.05 (1.46-2.87)	1.80 (1.46-2.23)	< .001	53 (NA)	NA	.143	Low (limited-suggestive)

© 2022 Catalá-López F et al. *JAMA Pediatrics*

Outcomes of interest	No of studies	Participants with ASD or ADHD, No.	Death cases, No.	Pooled RR (95% CI)	RR largest study (95% CI)	P value for Effect Estimate	I^2 (95% CI)	95% prediction interval	P value for heterogeneity	Confidence
Neoplasms, ASD male	1	18693	48	NA	1.79 (1.34-2.39)	< .001	NA	NA	NA	Very low (very uncertain)
Neoplasms, ASD female	1	8429	40	NA	1.83 (1.33-2.51)	< .001	NA	NA	NA	Very low (very uncertain)
Neoplasms, ASD first-degree relative (mother)	1	2041	10	NA	1.54 (0.80-2.90)	.189	NA	NA	NA	Very low (very uncertain)
Endocrine, ASD both	1	27122	19	NA	3.70 (2.34-5.87)	< .001	NA	NA	NA	Very low (very uncertain)
Endocrine, ASD male	1	18693	6	NA	2.11 (0.94-4.73)	.070	NA	NA	NA	Very low (very uncertain)
Endocrine, ASD female	1	8429	3	NA	5.70 (3.25-9.99)	< .001	NA	NA	NA	Very low (very uncertain)
Mental and behavioural disorders, ASD both	1	27122	30	NA	2.80 (1.94-4.03)	< .001	NA	NA	NA	Very low (very uncertain)
Mental and behavioural disorders, ASD male	1	18693	12	NA	3.31 (1.85-5.92)	< .001	NA	NA	NA	Very low (very uncertain)
Mental and behavioural disorders, ASD female	1	8429	18	NA	2.53 (1.58-4.05)	< .001	NA	NA	NA	Very low (very uncertain)
Nervous system, ASD both	4	61066	102	10.79 (5.42-21.10)	7.49 (5.78-9.72)	< .001	85 (64-94)	0.52-219.57	< .001	Low (limited-suggestive)
Nervous system, ASD male	1	18693	38	NA	10.19 (7.27-14.29)	< .001	NA	NA	NA	Very low (very uncertain)
Nervous system, ASD female	1	8429	24	NA	5.29 (3.50-7.99)	< .001	NA	NA	NA	Very low (very uncertain)
Circulatory system, ASD both	1	27122	157	NA	1.49 (1.27-1.75)	<.001	NA	NA	NA	Very low (very uncertain)
Circulatory system, ASD male	1	18693	91	NA	2.02 (1.64-2.49)	<.001	NA	NA	NA	Very low (very uncertain)
Circulatory system, ASD female	1	8429	66	NA	1.10 (0.86-1.40)	.443	NA	NA	NA	Very low (very uncertain)
Respiratory system, ASD both	2	40233	62	3.87 (1.80-8.32)	2.68 (1.99-3.62)	.001	86 (NA)	NA	.007	Low (limited-suggestive)
Respiratory system, ASD male	1	18693	19	NA	2.16 (1.36-3.43)	.001	NA	NA	NA	Very low (very uncertain)
Respiratory system, ASD female	1	8429	26	NA	3.24 (2.19-4.80)	<.001	NA	NA	NA	Very low (very uncertain)
Digestive system, ASD both	2	40233	40	4.59 (2.29-9.19)	3.31 (2.25-4.87)	<.001	76 (NA)	NA	.039	Low (limited-suggestive)
Digestive system, ASD male	1	18693	16	NA	3.81 (2.31-6.29)	<.001	NA	NA	NA	Very low (very uncertain)
Digestive system, ASD female	1	8429	11	NA	2.78 (1.52-5.07)	.001	NA	NA	NA	Very low (very uncertain)

© 2022 Catalá-López F et al. *JAMA Pediatrics*

Outcomes of interest	No of studies	Participants with ASD or ADHD, No.	Death cases, No.	Pooled RR (95% CI)	RR largest study (95% CI)	P value for Effect Estimate	I^2 (95% CI)	95% prediction interval	P value for heterogeneity	Confidence
Genitourinary system, ASD both	1	27122	12	NA	3.82 (2.13-6.84)	<.001	NA	NA	NA	Very low (very uncertain)
Congenital malformations, ASD both	2	40233	37	11.74 (4.49-30.74)	19.10 (11.94-30.55)	< .001	87 (NA)	NA	.005	Low (limited-suggestive)
Congenital malformations, ASD male	1	18693	8	NA	11.07 (5.30-23.13)	< .001	NA	NA	NA	Very low (very uncertain)
Congenital malformations, ASD female	1	8429	13	NA	33.86 (18.04-63.56)	< .001	NA	NA	NA	Very low (very uncertain)
External causes, ASD first-degree relative (mother)	1	158	1	NA	1.54 (0.06-37.32)	.792	NA	NA	NA	Very low (very uncertain)
External causes, ASD first-degree relative (father)	1	158	2	NA	2.56 (0.12-52.75)	.545	NA	NA	NA	Very low (very uncertain)

Note: Predictive intervals were inestimable with less than 3 studies.

eFigure 1. Secondary outcome. Unnatural causes of death among ASD/ADHD people.

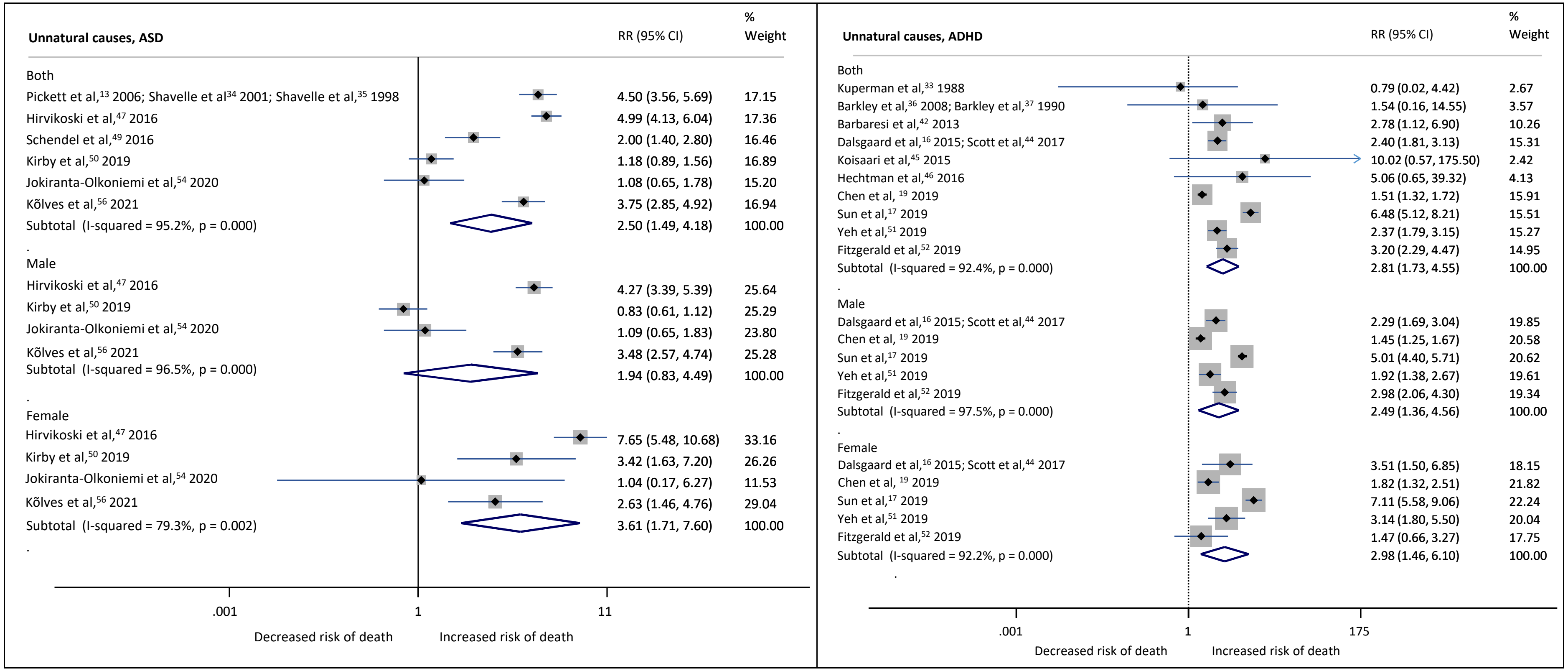

© 2022 Catalá-López F et al. *JAMA Pediatrics*

eFigure 2. Secondary outcome. Natural causes of death among ASD/ADHD people.

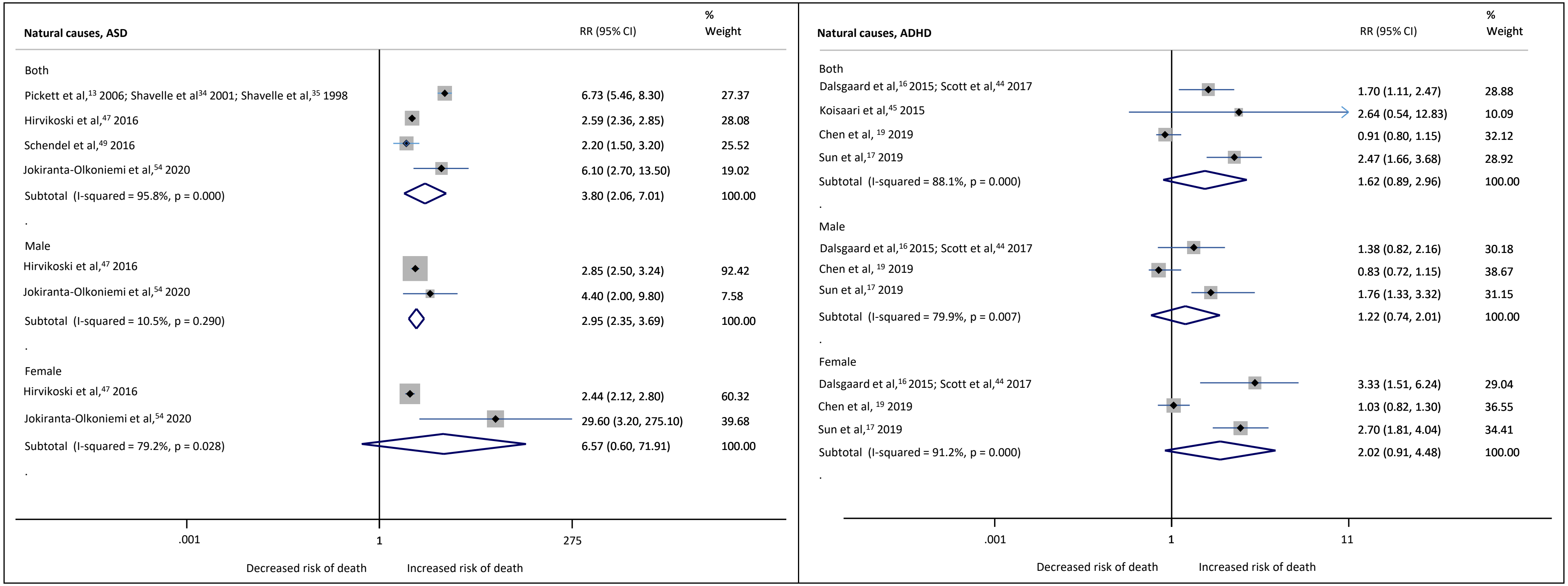

© 2022 Catalá-López F et al. *JAMA Pediatrics*

eFigure 3. Secondary outcome. Deaths caused by nervous system disorders among ASD people.

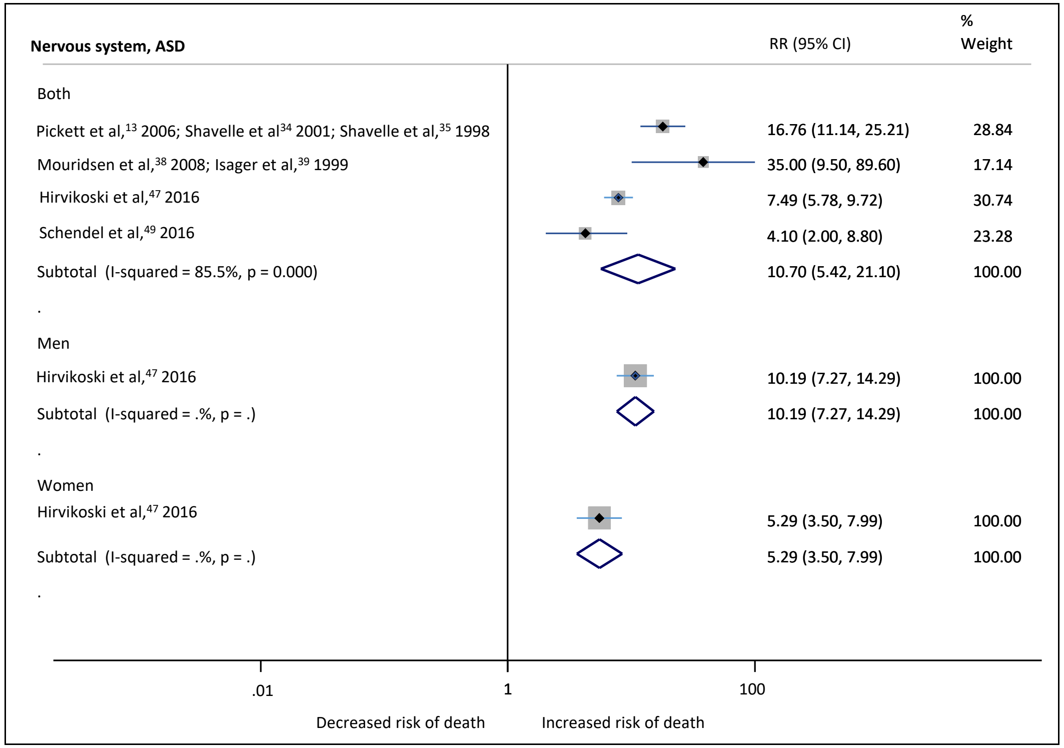

© 2022 Catalá-López F et al. *JAMA Pediatrics*

eFigure 4. Secondary outcome. Deaths caused by neoplasms among ASD people.

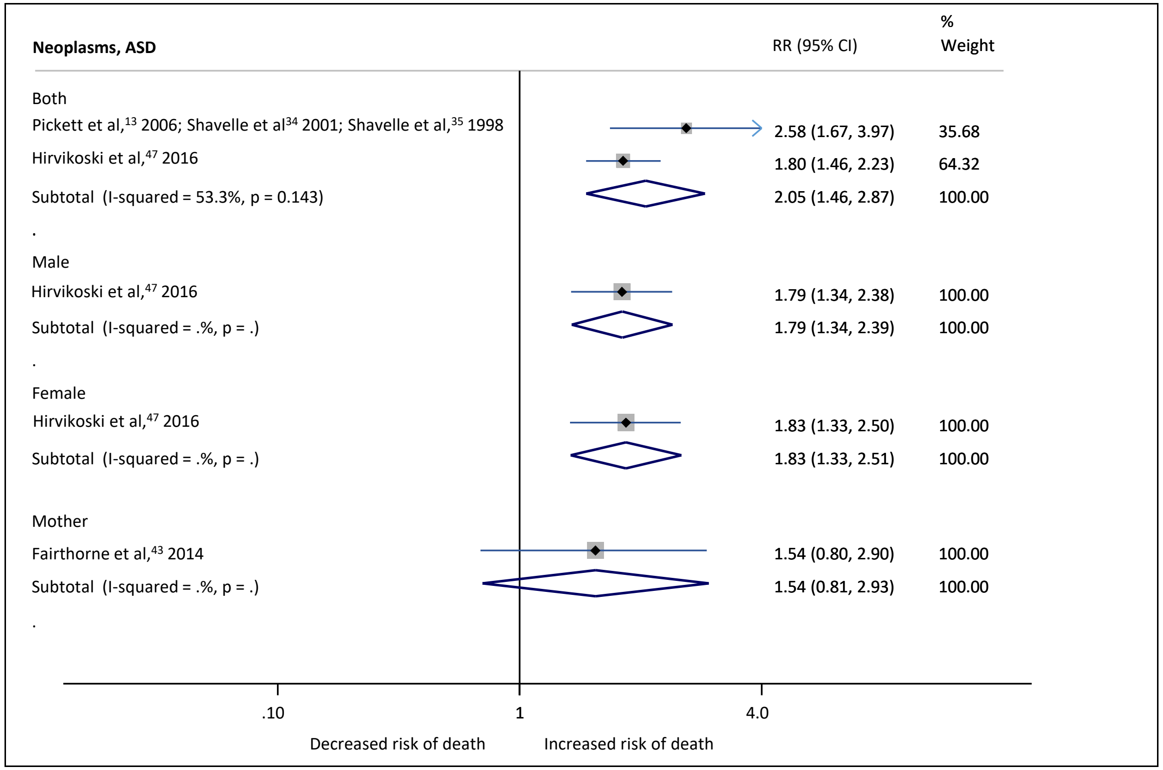

© 2022 Catalá-López F et al. *JAMA Pediatrics*

eFigure 5. Secondary outcome. Deaths caused by respiratory system diseases among ASD people.

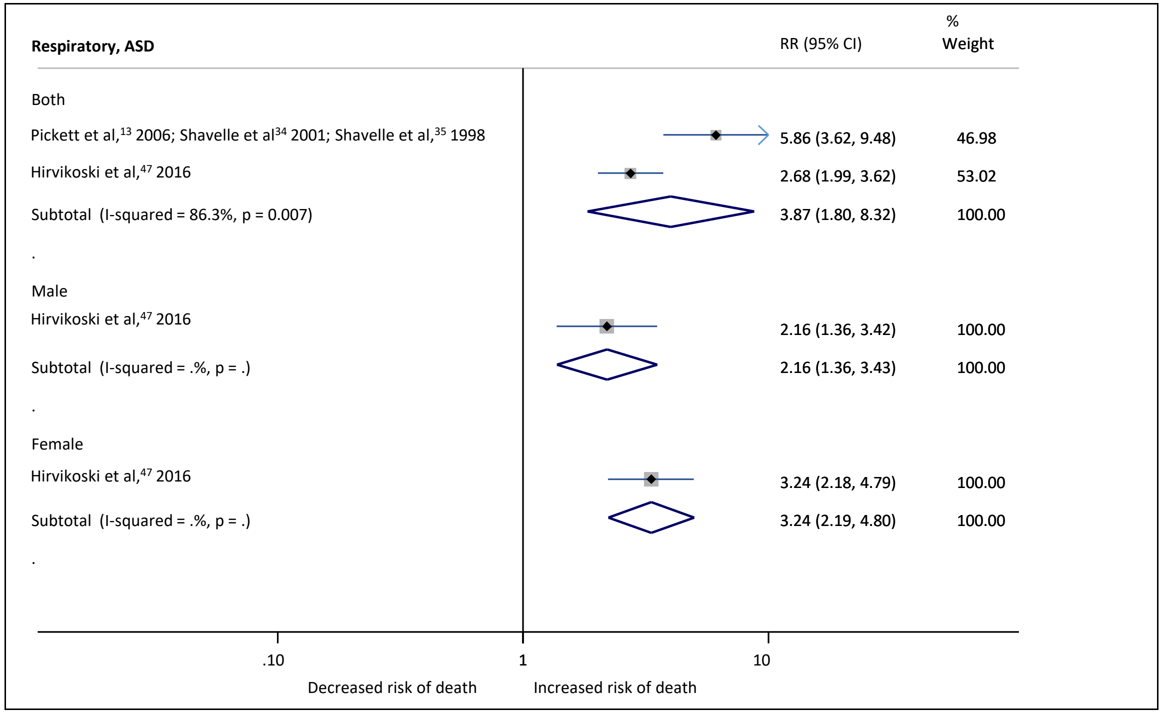

© 2022 Catalá-López F et al. *JAMA Pediatrics*

eFigure 6. Secondary outcome. Deaths caused by digestive system diseases among ASD people.

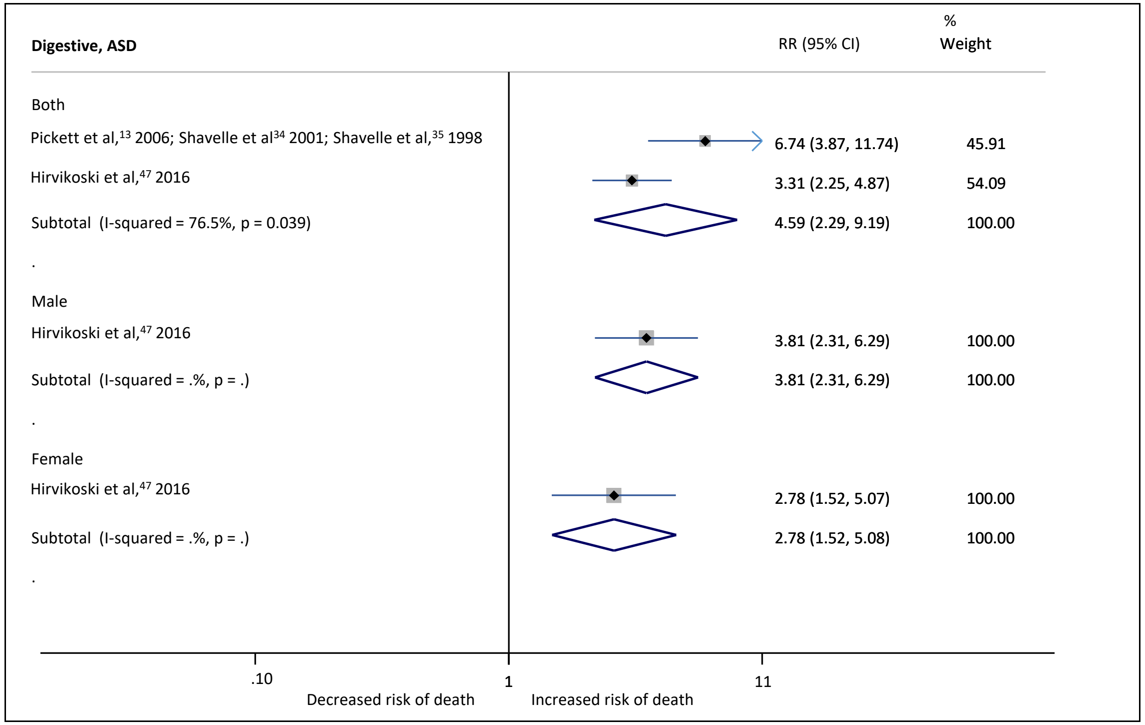

© 2022 Catalá-López F et al. *JAMA Pediatrics*

eFigure 7. Secondary outcome. Deaths caused by congenital malformations among ASD people.

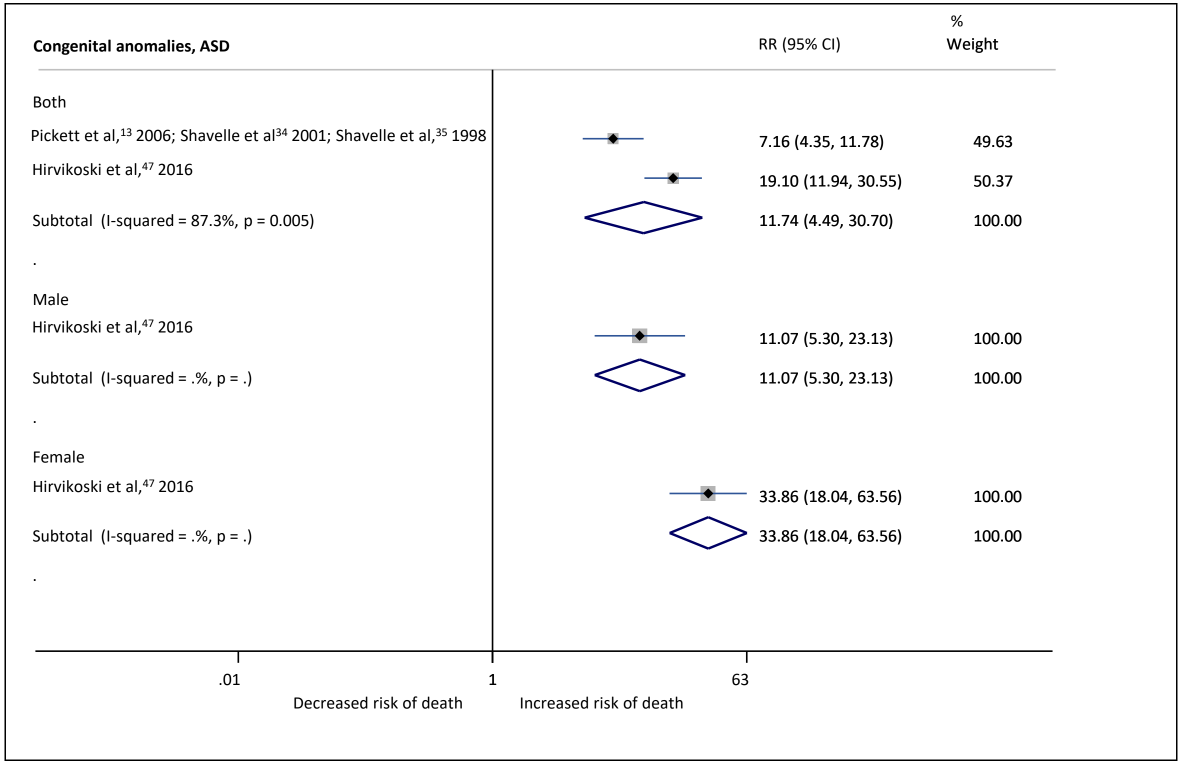

© 2022 Catalá-López F et al. *JAMA Pediatrics*

eFigure 8. Subgroup analyses. ASD comorbidity and all-cause mortality among ASD people.

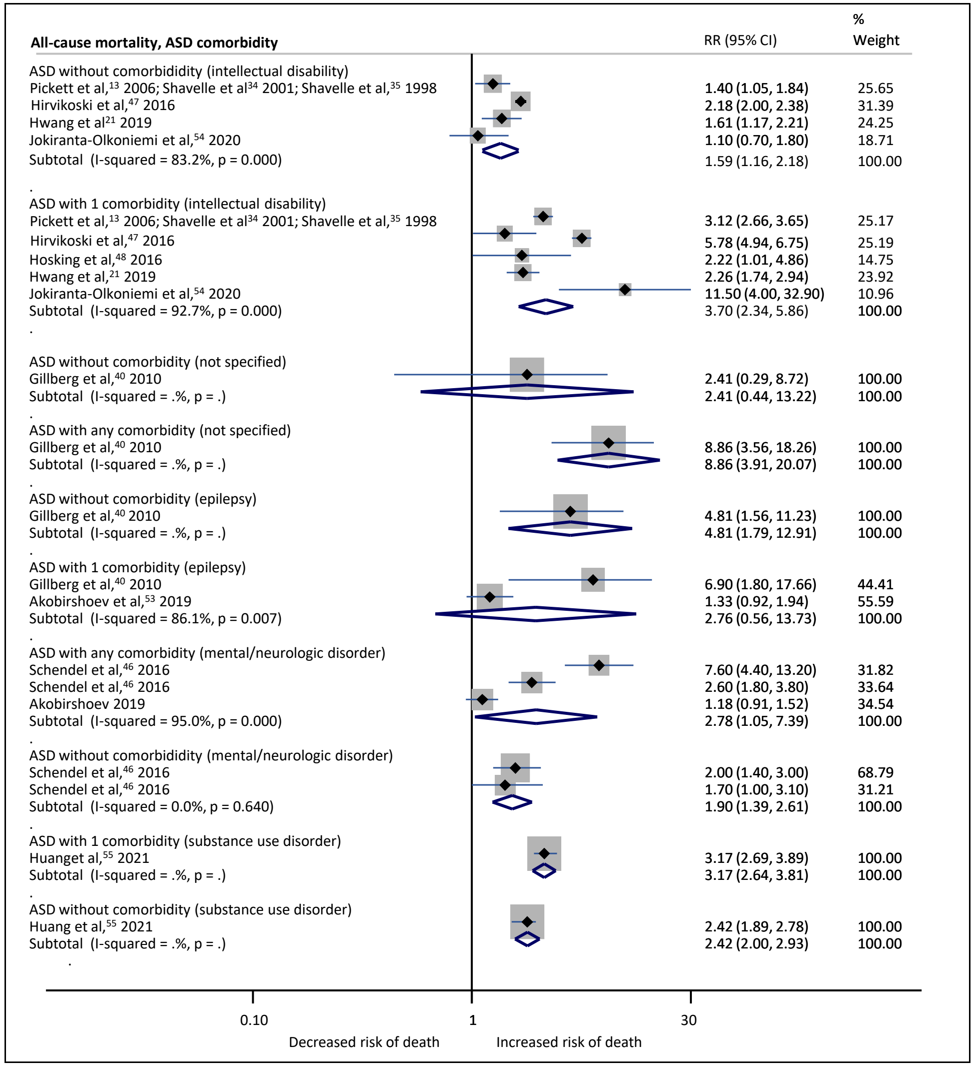

© 2022 Catalá-López F et al. *JAMA Pediatrics*

eFigure 9. Subgroup analyses. ADHD comorbidity and all-cause mortality among ADHD people.

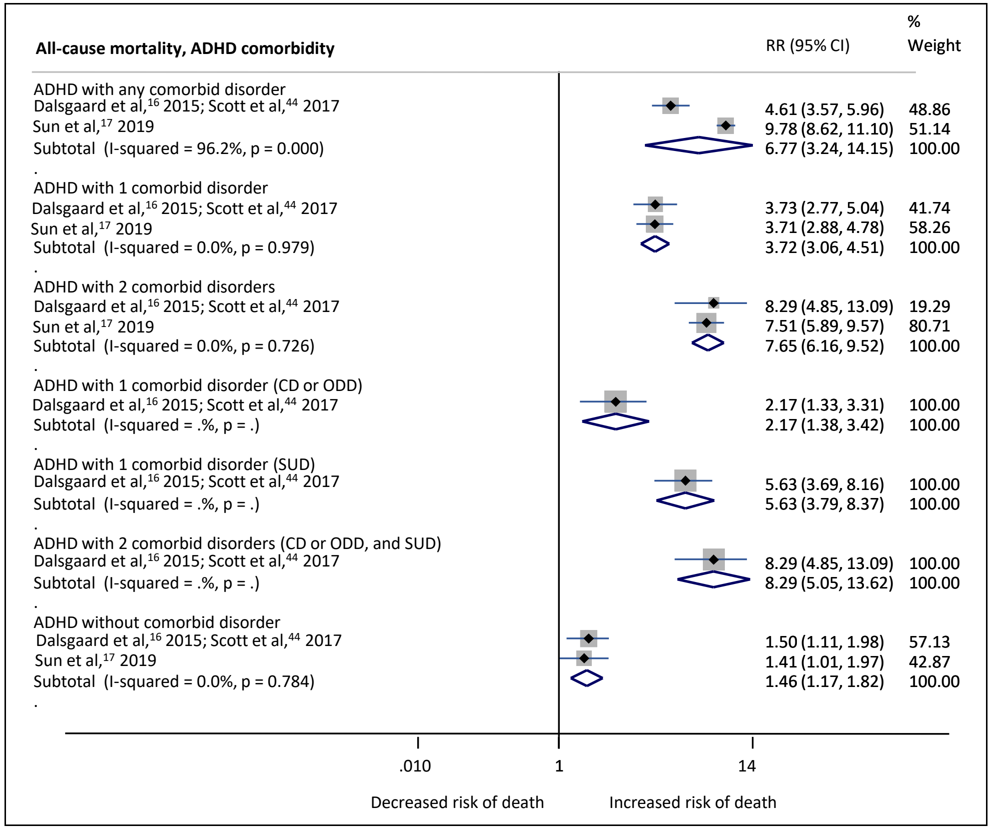

© 2022 Catalá-López F et al. *JAMA Pediatrics*

eFigure 10. Subgroup analyses. Age group at first diagnosis and all-cause mortality among ASD people.

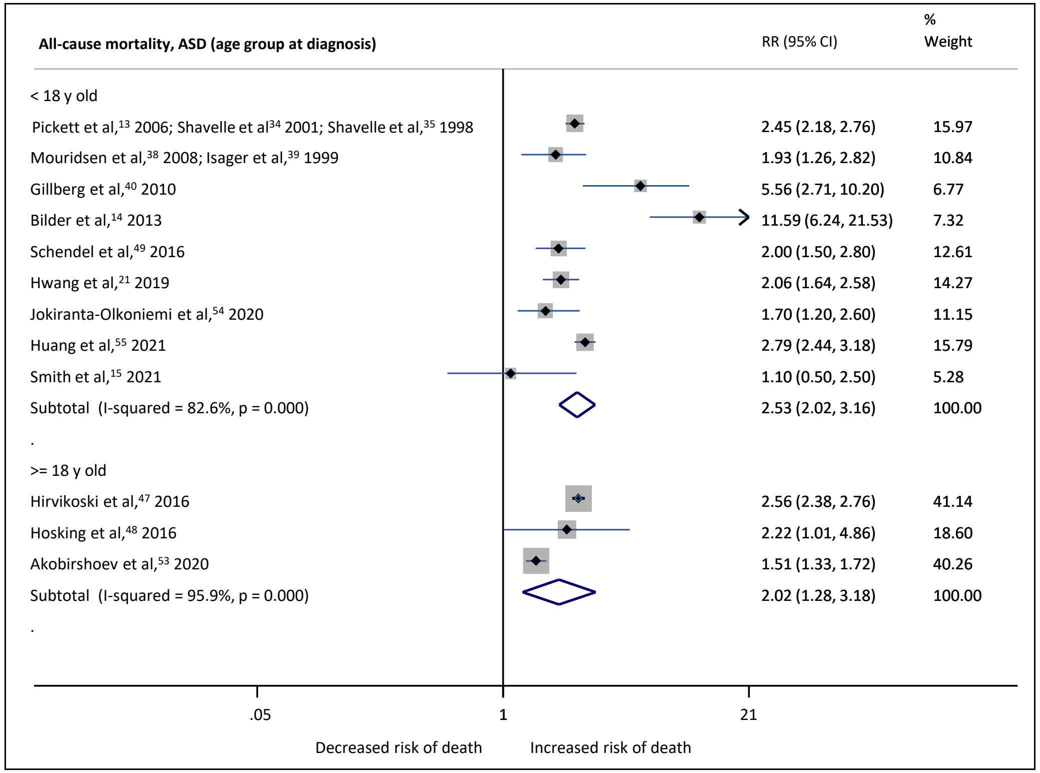

© 2022 Catalá-López F et al. *JAMA Pediatrics*

eFigure 11. Subgroup analyses. Age group at first diagnosis and all-cause mortality among ADHD people.

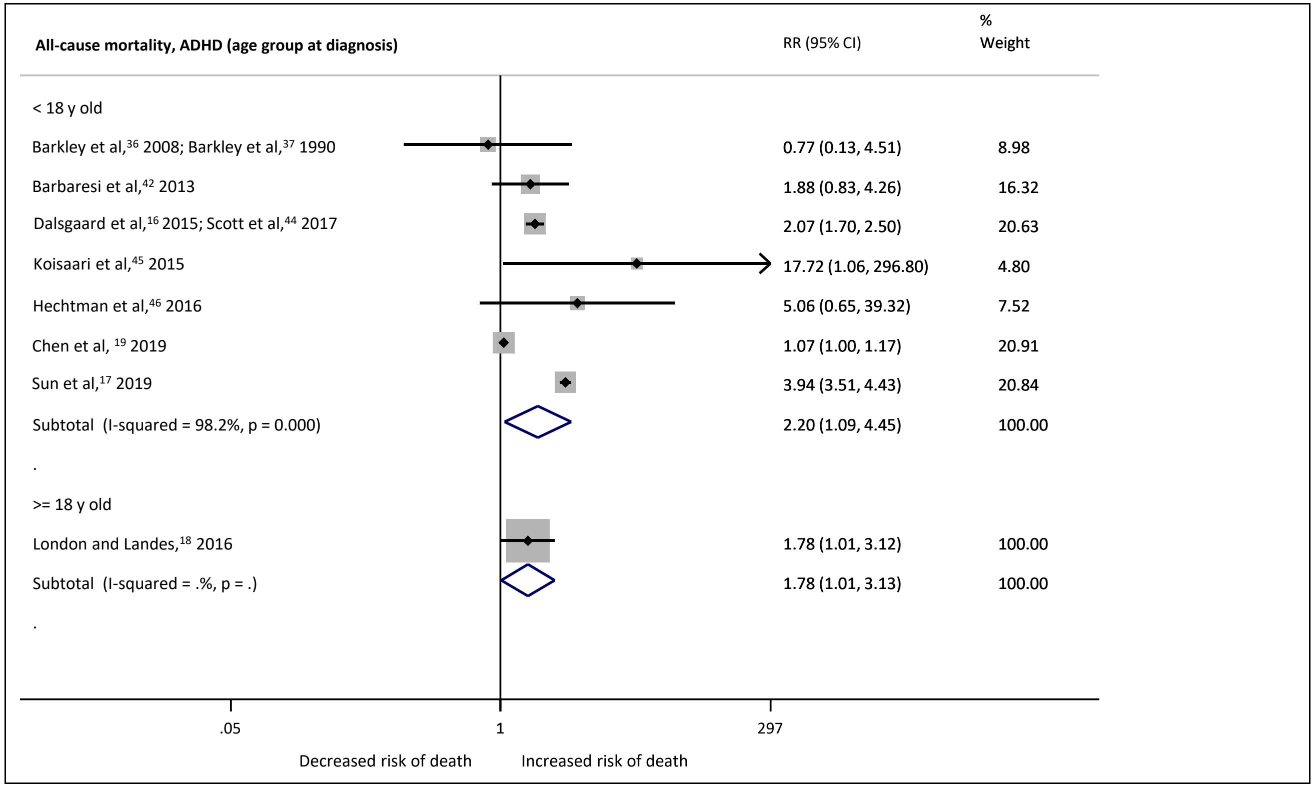

© 2022 Catalá-López F et al. *JAMA Pediatrics*

eFigure 12. Subgroup analyses. Cohort/sample size and and all-cause mortality among ASD people.

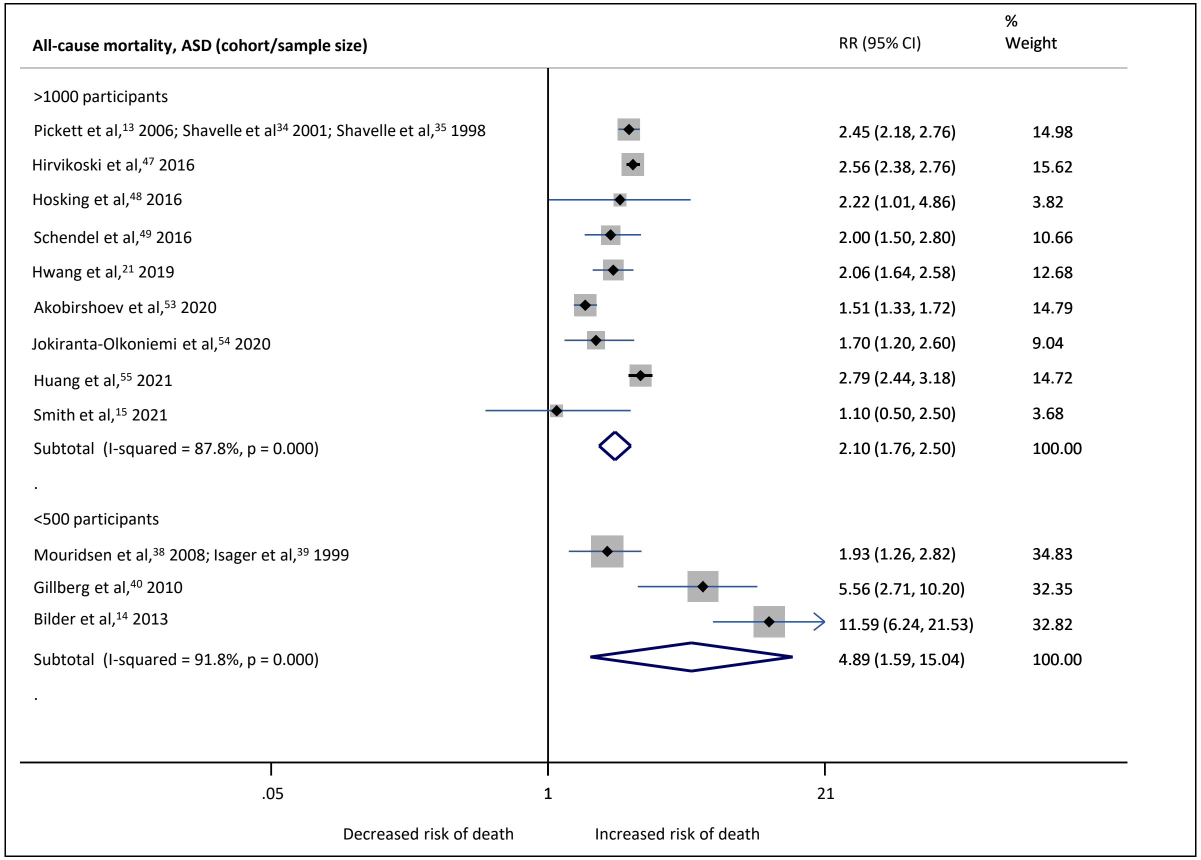

© 2022 Catalá-López F et al. *JAMA Pediatrics*

eFigure 13. Subgroup analyses. Cohort/sample size and and all-cause mortality among ADHD people.

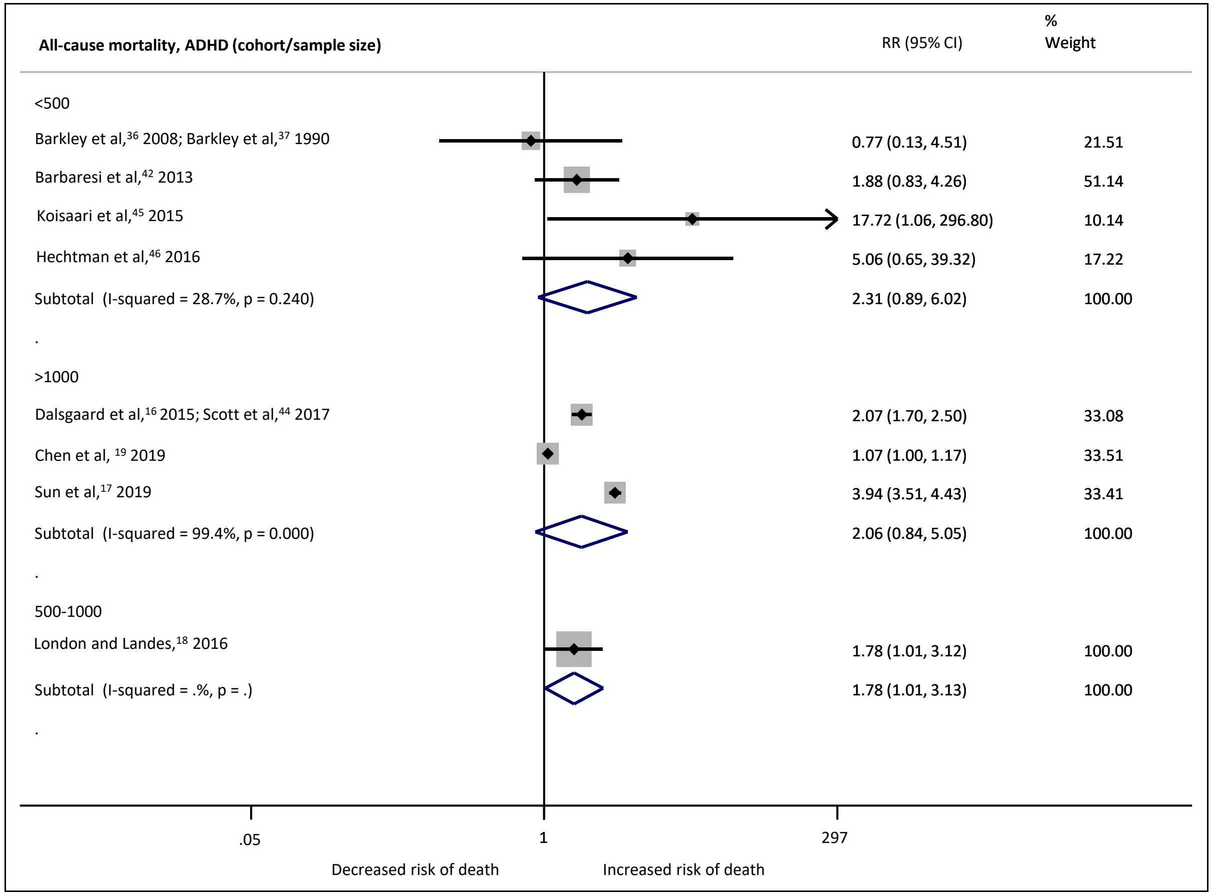

© 2022 Catalá-López F et al. *JAMA Pediatrics*

eFigure 14. Subgroup analyses. Setting and all-cause mortality among ASD people.

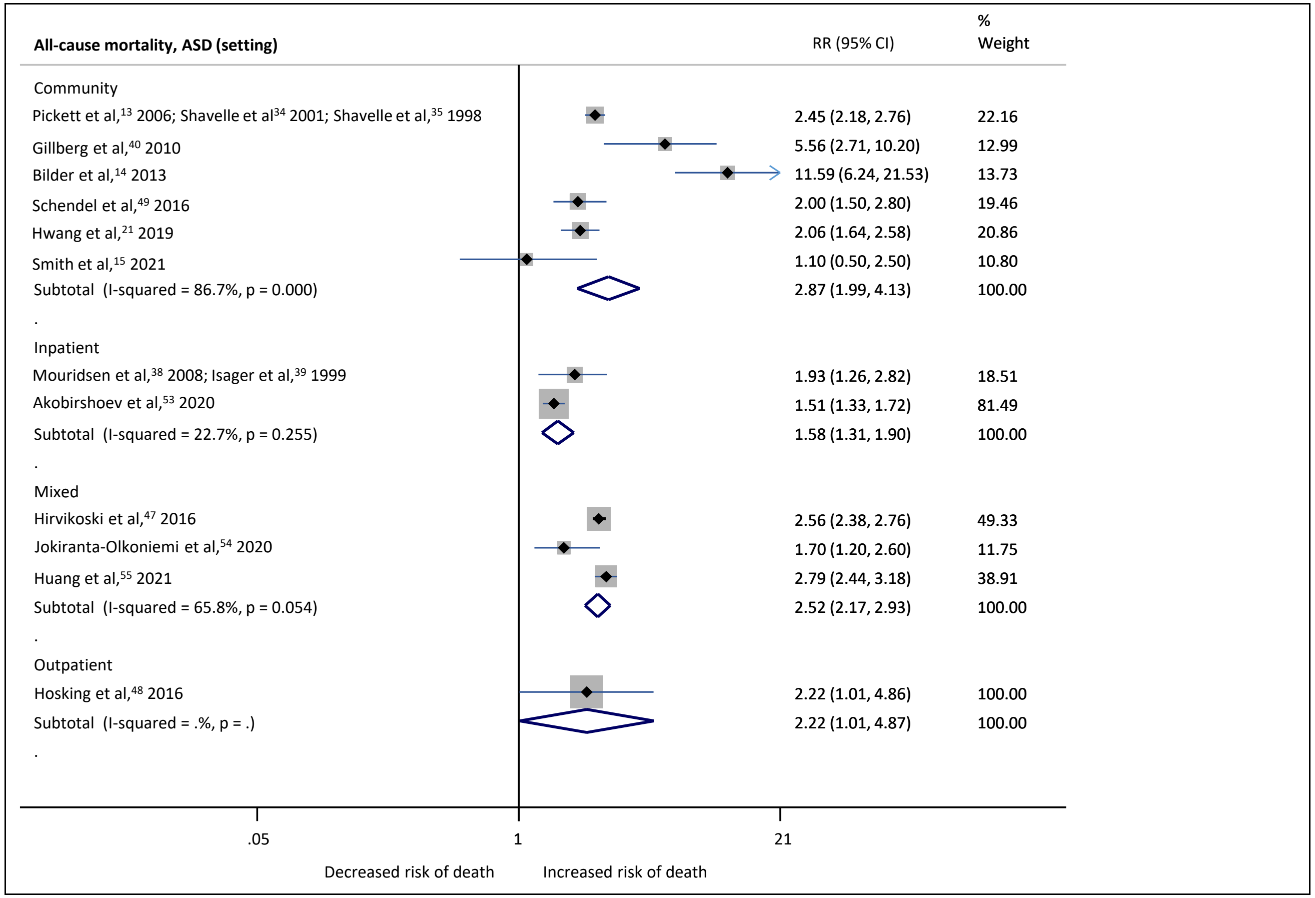

© 2022 Catalá-López F et al. *JAMA Pediatrics*

eFigure 15. Subgroup analyses. Setting and all-cause mortality among ADHD people.

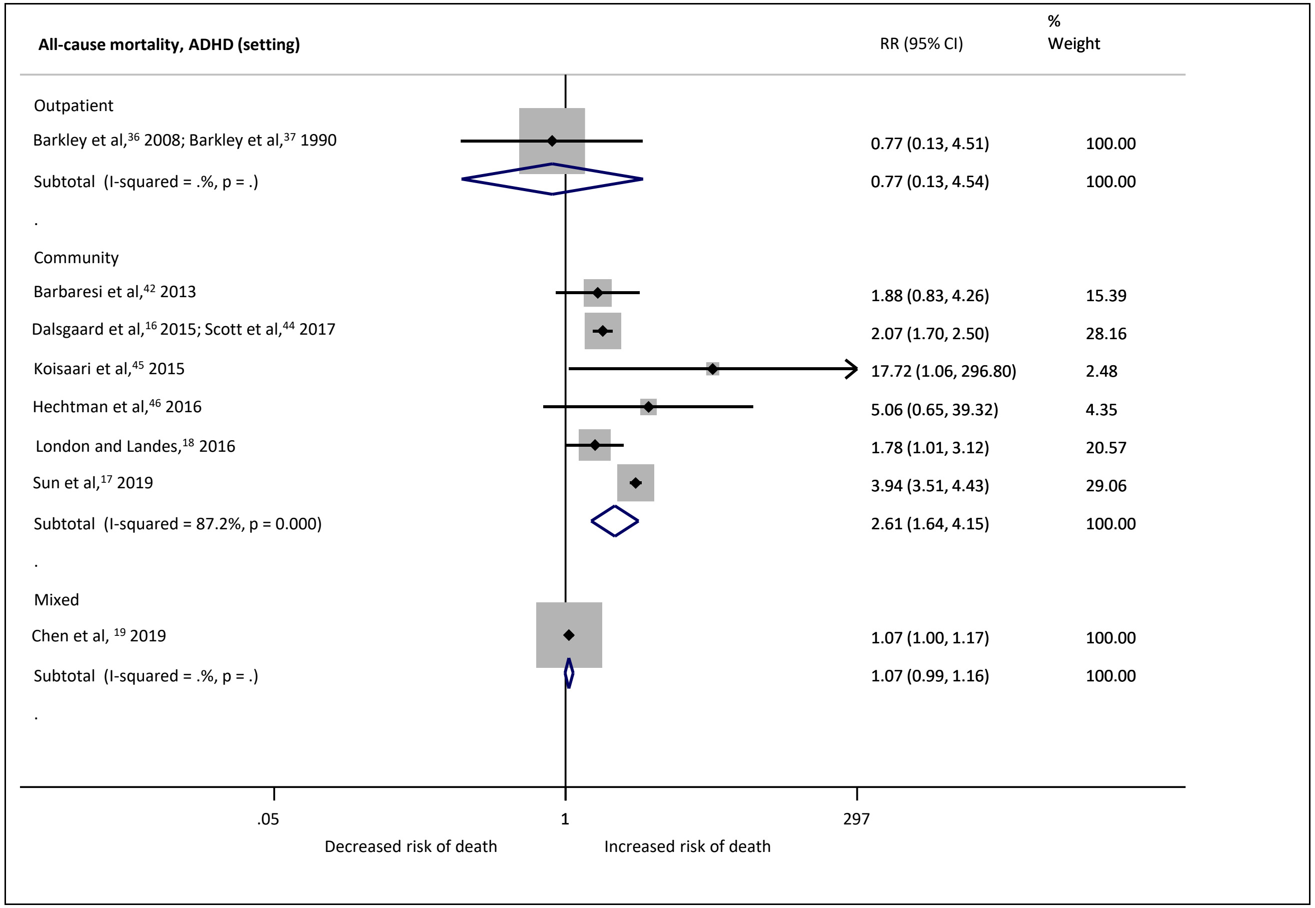

© 2022 Catalá-López F et al. *JAMA Pediatrics*

eFigure 16. Subgroup analyses. Follow-up (in years) and all-cause mortality among ASD people.

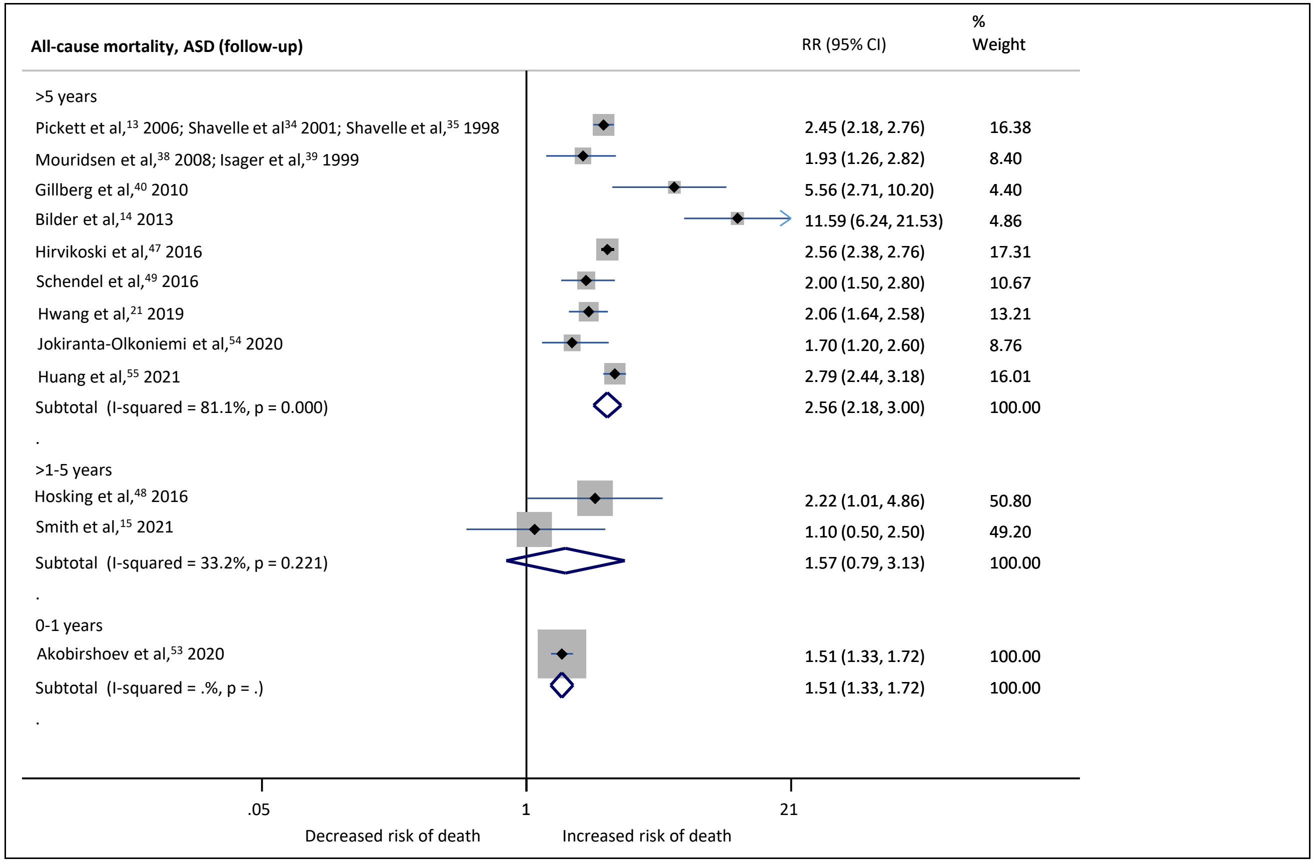

© 2022 Catalá-López F et al. *JAMA Pediatrics*

eFigure 17. Subgroup analyses. Follow-up (in years) and all-cause mortality among ADHD people.

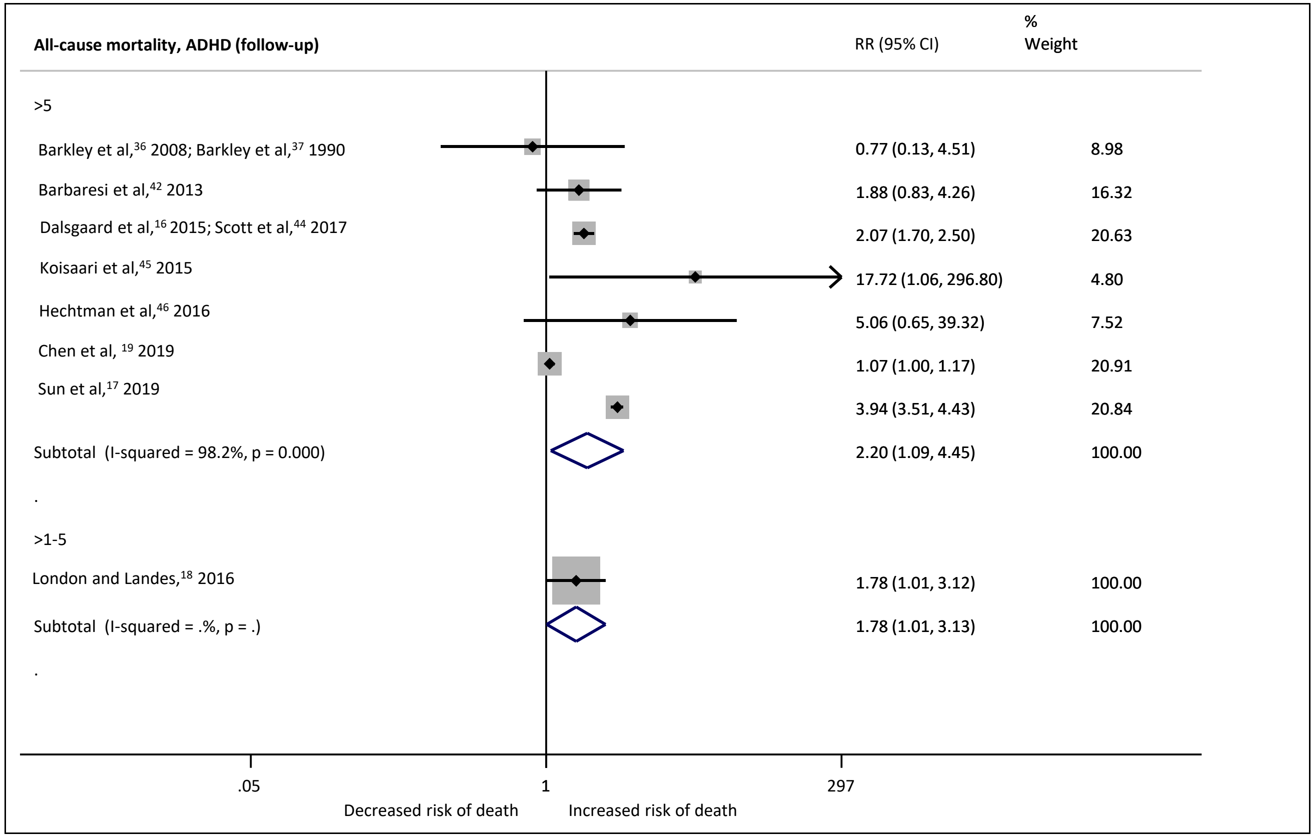

© 2022 Catalá-López F et al. *JAMA Pediatrics*

eFigure 18. Subgroup analyses. Study-quality/risk of bias and all-cause mortality among ASD people.

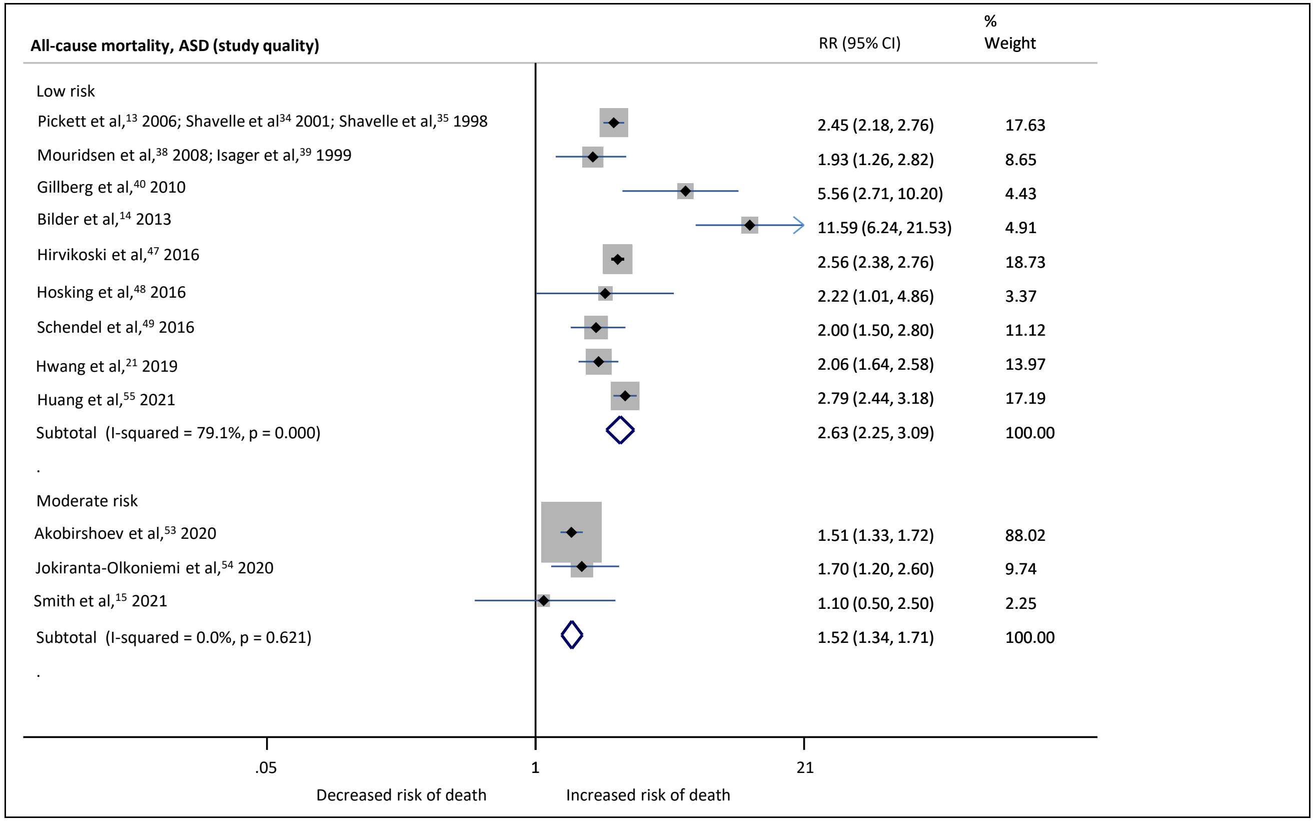

© 2022 Catalá-López F et al. *JAMA Pediatrics*

eFigure 19. Subgroup analyses. Study-quality/risk of bias and all-cause mortality among ADHD people.

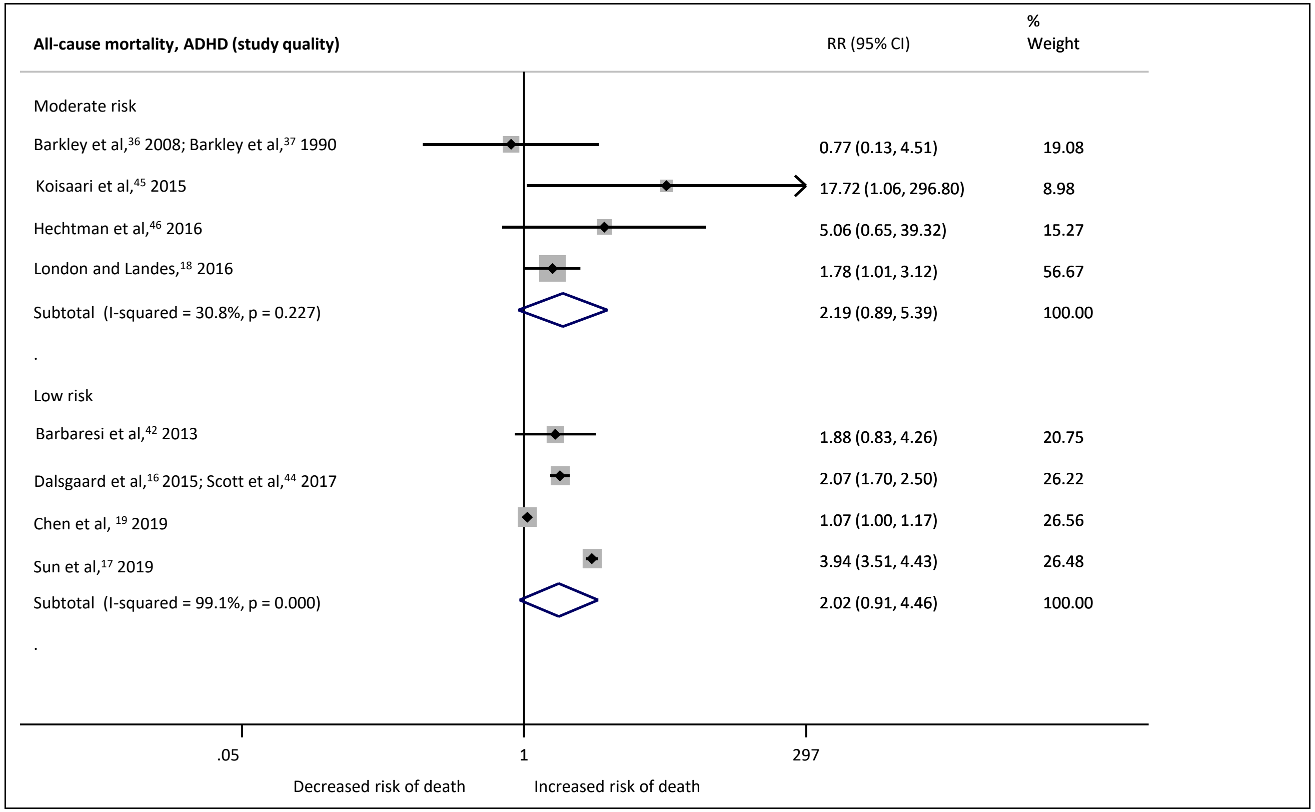

© 2022 Catalá-López F et al. *JAMA Pediatrics*

eFigure 20. Subgroup analyses. Adjustment for potential confounders and all-cause mortality among ASD people.

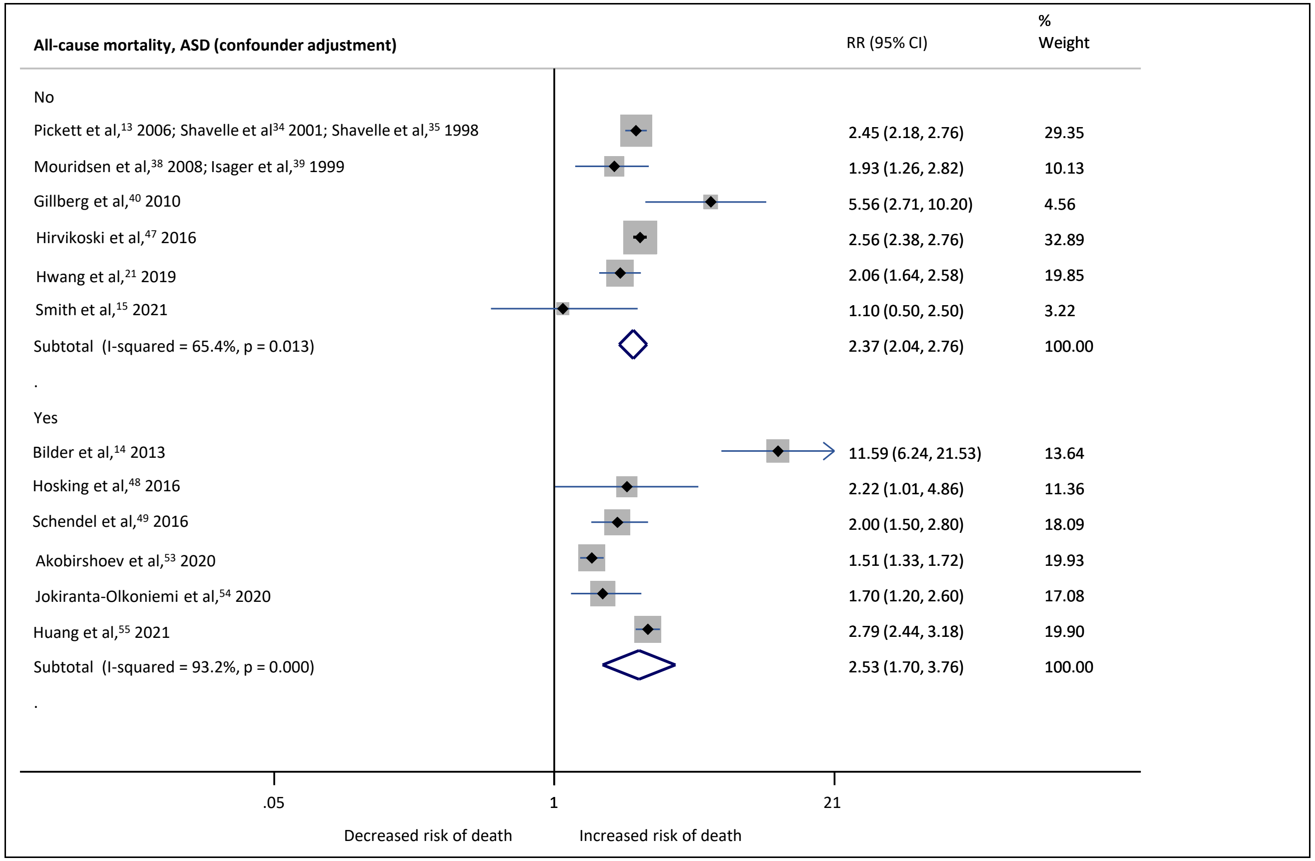

© 2022 Catalá-López F et al. *JAMA Pediatrics*

eFigure 21. Subgroup analyses. Adjustment for potential confounders and all-cause mortality among ADHD people.

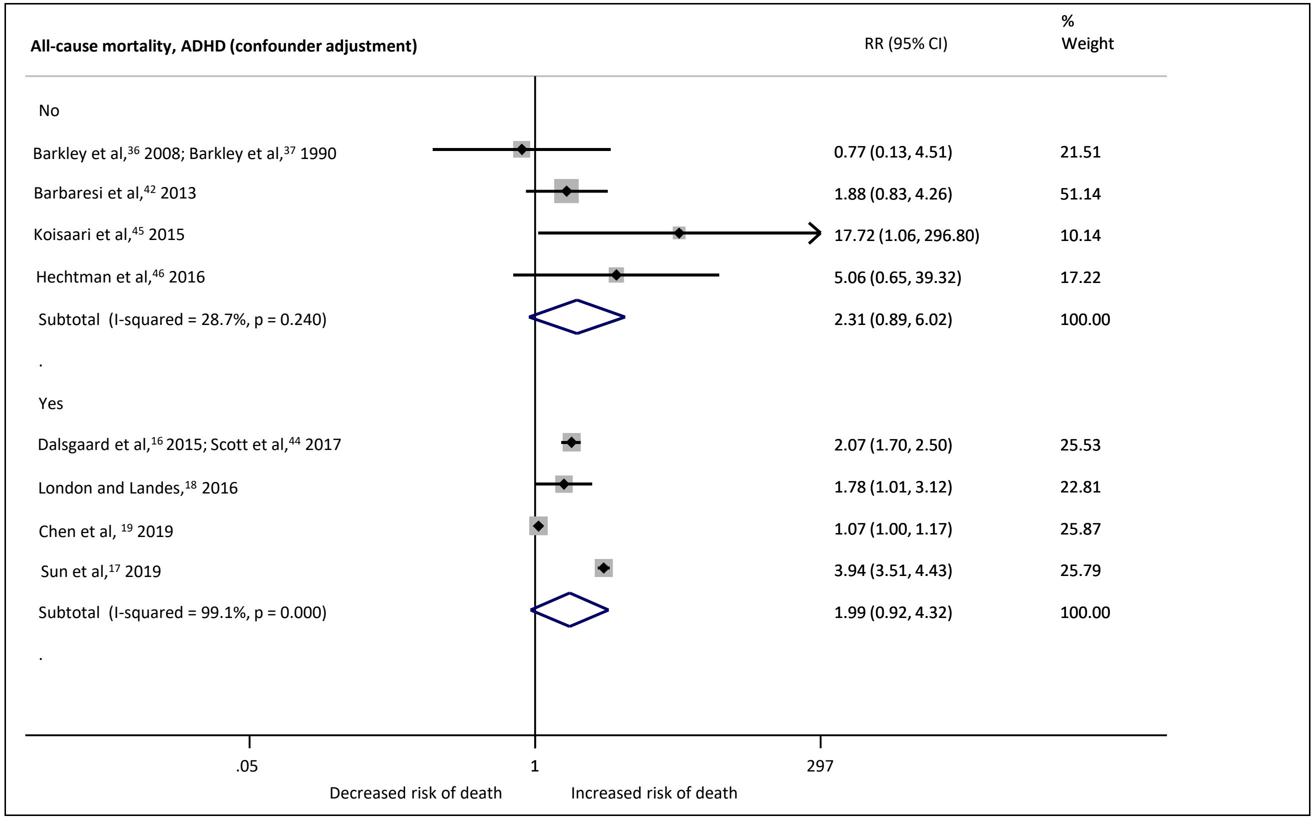

© 2022 Catalá-López F et al. *JAMA Pediatrics*

eFigure 22. Funnel plot for primary outcome of all-cause mortality in people with ASD.

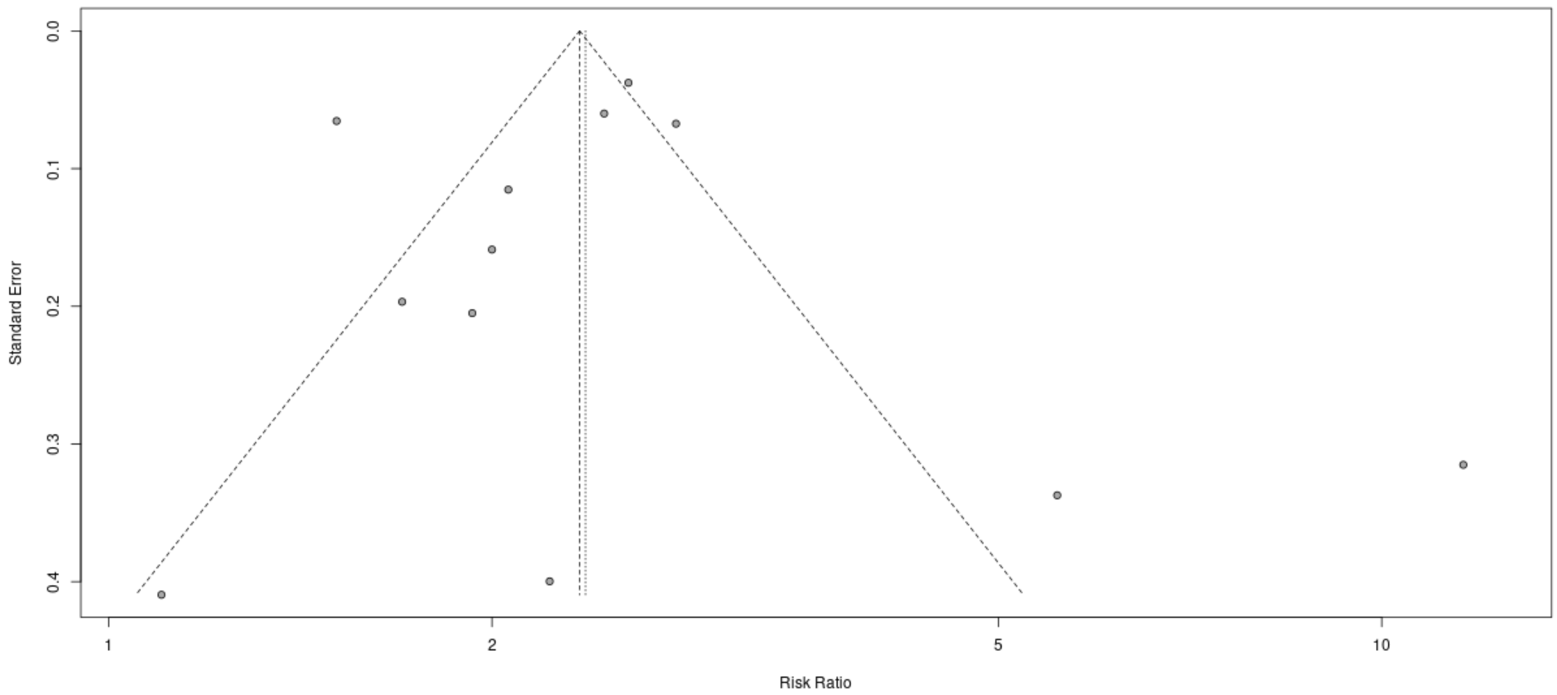

Begg test (*P* = 0.681), and Egger test (*P* = 0.248).
12 studies

© 2022 Catalá-López F et al. *JAMA Pediatrics*

eFigure 23. Treemap summarizing the amount of data according to specific causes of death.

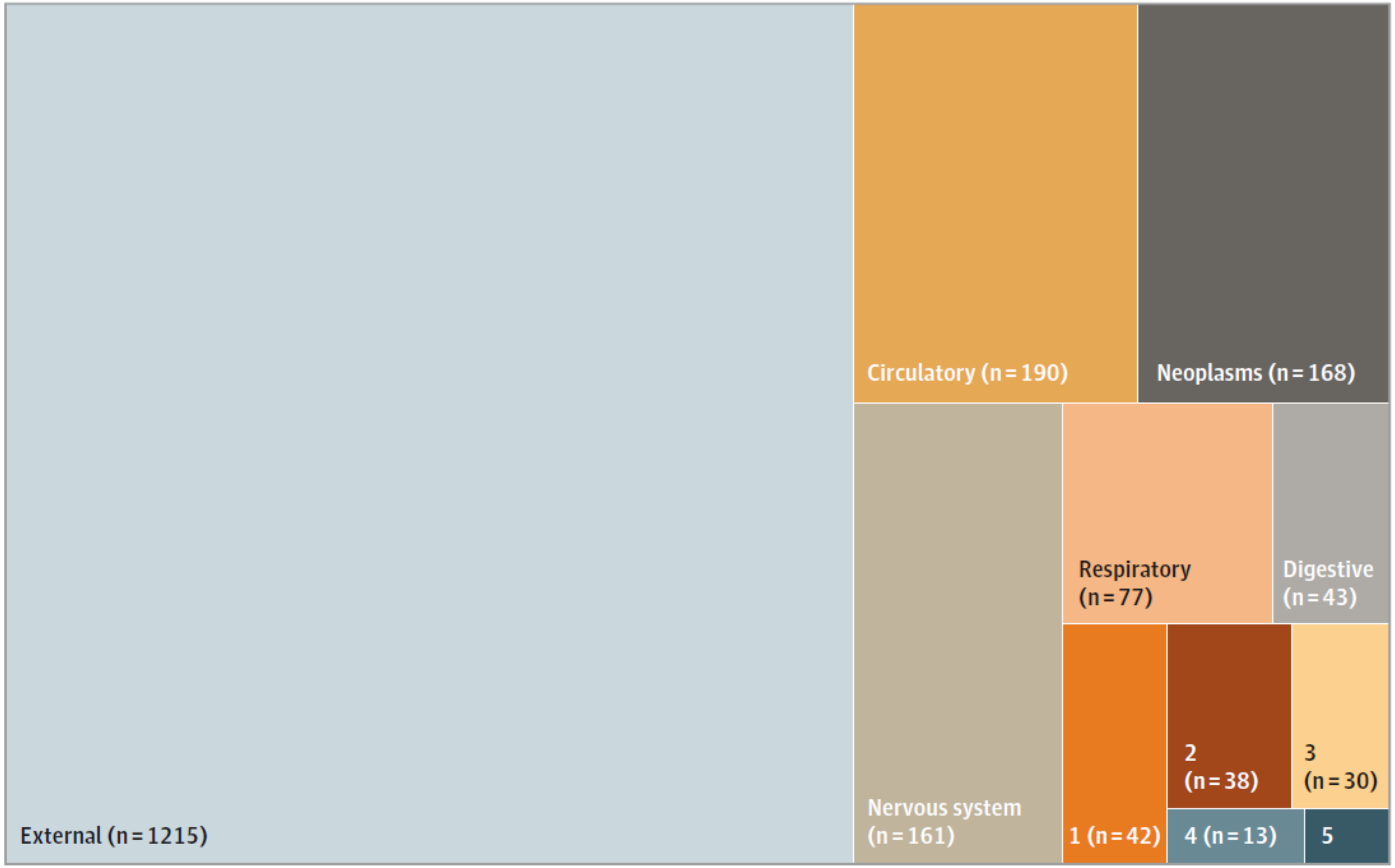

1 Indicates congenital anomalies (42 cases); 2, mental and behavioral disorders (38 cases); 3, endocrine, nutritional, and metabolic diseases (30 cases); 4, genitourinary system diseases (13 cases); and 5, infectious diseases (8 cases).

© 2022 Catalá-López F et al. *JAMA Pediatrics*
